# C−H Activation of Inert Arenes using a Photochemically Activated Guanidinato‐Magnesium(I) Compound

**DOI:** 10.1002/chem.202202103

**Published:** 2022-09-26

**Authors:** Jeremy C. Mullins, K. Yuvaraj, Yixiao Jiang, Gerard P. Van Trieste, Asim Maity, David C. Powers, Cameron Jones

**Affiliations:** ^1^ School of Chemistry PO Box 23 Monash University Melbourne, <countryPartVIC 3800 Australia; ^2^ Department of Chemistry Texas A&M University College Station, TX 77843 USA

**Keywords:** C−H activation, low oxidation state, magnesium(I), photochemical, radical

## Abstract

UV irradiation of solutions of a guanidinate coordinated dimagnesium(I) compound, [{(Priso)Mg}_2_] **3** (Priso=[(DipN)_2_CNPr^*i*^_2_]^−^, Dip=2,6‐diisopropylphenyl), in either benzene, toluene, the three isomers of xylene, or mesitylene, leads to facile activation of an aromatic C−H bond of the solvent in all cases, and formation of aryl/hydride bridged magnesium(II) products, [{(Priso)Mg}_2_(μ‐H)(μ‐Ar)] **4**–**9**. In contrast to similar reactions reported for β‐diketiminate coordinated counterparts of **3**, these C−H activations proceed with little regioselectivity, though they are considerably faster. Reaction of **3** with an excess of the pyridine, *p*‐NC_5_H_4_Bu^*t*^ (py^Bu*t*^), gave [(Priso)Mg(py^Bu*t*^H)(py^Bu*t*^)_2_] **10**, presumably via reduction of the pyridine to yield a radical intermediate, [(Priso)Mg(py^Bu*t*^⋅)(py^Bu*t*^)_2_] **11**, which then abstracts a proton from the reaction solvent or a reactant. DFT calculations suggest two possible pathways to the observed arene C−H activations. One of these involves photochemical cleavage of the Mg−Mg bond of **3**, generating magnesium(I) doublet radicals, (Priso)Mg⋅. These then doubly reduce the arene substrate to give “Birch‐like” products, which subsequently rearrange via C−H activation of the arene. Circumstantial evidence for the photochemical generation of transient magnesium radical species includes the fact that irradiation of a cyclohexane solution of **3** leads to an intramolecular aliphatic C−H activation process and formation of an alkyl‐bridged magnesium(II) species, [{Mg(μ‐Priso^−H^)}_2_] **12**. Furthermore, irradiation of a 1 : 1 mixture of **3** and the β‐diketiminato dimagnesium(I) compound, [{(^Dip^Nacnac)Mg}_2_] (^Dip^Nacnac=[HC(MeCNDip)_2_]^−^), effects a “scrambling” reaction, and the near quantitative formation of an unsymmetrical dimagnesium(I) compound, [(Priso)Mg−Mg(^Dip^Nacnac)] **13**. Finally, the EPR spectrum (77 K) of a glassed solution of UV irradiated **3** is dominated by a broad featureless signal, indicating the presence of a doublet radical species.

## Graphical abstract

**A radical approach**. Activations of arene C−H bonds by a photochemically excited dimagnesium(I) compound are reported (see picture, Dip=2,6‐diisopropylphenyl). Experimental and theoretical evidence for the intermediacy of magnesium radicals in these reactions is presented. Such transition‐metal‐free C−H bond activations hold considerable potential for organic synthesis.

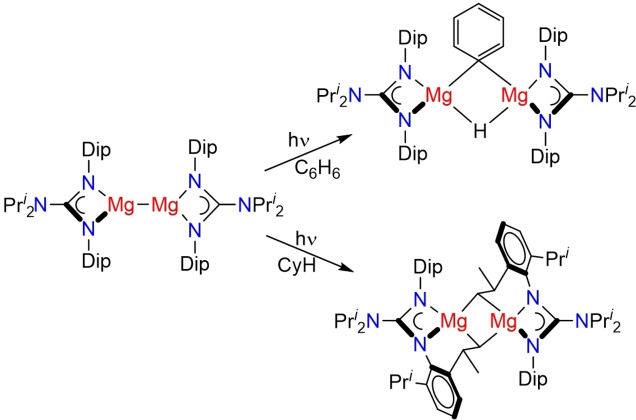

## Introduction

Chemical processes involving the activation of strong, weakly polar C−H bonds in simple arenes and alkanes are integral to numerous value‐adding syntheses of larger, functionalised molecules; for example, natural products, agrochemicals and pharmaceuticals.^[1]^ Such synthetic transformations have relied heavily on second and third row d‐block metal complexes as catalysts or stoichiometric reagents for the facile and selective C−H activation of organic substrates.^[1]^ Despite their effectiveness in this arena, the 4d‐ and 5d‐metals used for C−H activations are often expensive, toxic, and sources of their ores are becoming increasingly scarce. To counter these problems, a great deal of research has gone into the development of cheaper, more environmentally benign alternatives, especially those incorporating earth abundant metals from the first row of the d‐block of the periodic table. While that work initially faced significant obstacles, the use of 3d‐metal complexes for C−H activations is now commonplace.^[2]^

More recently, the use of reactive main group metal compounds for the C−H functionalization of organic molecules has been explored.^[3]^ Leaders in this emerging field include Mulvey, Knochel, Hevia and others,^[4]^ who have developed numerous main group heterobimetallic systems, for example turbo‐Grignard reagents and turbo‐Hauser bases, for the synergistic and selective deprotonation(s) of a multitude of unsaturated substrates. In all these reactive systems, the main group metal(s) exist in their “normal” oxidation state. Considering the rapid expansion of knowledge surrounding the stabilization of low oxidation state main group compounds, it is not surprizing that these potentially redox active species are also finding use in C−H activation reactivity. Of relevance to the current study, and from the p‐block, is work from a number of groups (e. g., those of Harder,^[5]^ Crimmin,^[6]^ Yamashita,^[7]^ Coles, Mulvey,^[8]^ Hill,^[9]^ Aldridge and Goicoechea^[10]^), who have used both neutral and anionic nucleophilic aluminum(I) reagents for the activation of C−H bonds in normally inert arenes (e. g., benzene, toluene and xylenes), both in the presence or absence of transition metal catalysts. These reactions deliver varying degrees of product selectivity, and typically proceed via the formal oxidative addition of the C−H bond to an Al^I^ center.

Arene C−H activation reactions are much less known for low oxidation state s‐block compounds, and well‐defined examples can be confined to those carried out by magnesium(I) compounds. Crimmin and co‐workers have shown that β‐diketiminate coordinated Mg−Mg bonded systems can functionalize a C−H bond of benzene in the presence of a Pd^0^ catalyst.^[11]^ Similarly, Harder and co‐workers reported that in situ or ball‐mill generated “magnesium(I) radicals” can doubly reduce benzene to give a “Birch‐like” product, which upon heating yields magnesium hydride and magnesium phenyl products, via the formal activation of a benzene C−H bond.^[12]^

**Scheme 1:**
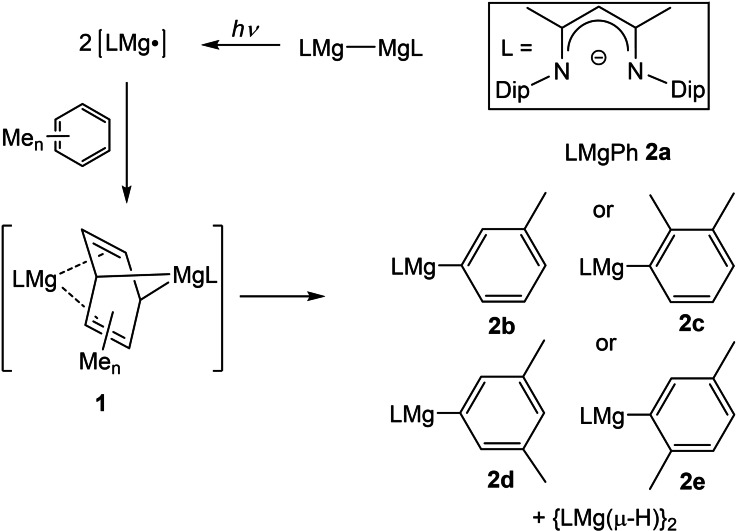
C−H activation of arenes by a photochemically activated β‐diketiminate ligated dimagnesium(I) compound (Dip=2,6‐diisopropylphenyl).

As an extension of those studies, and as part of our systematic efforts to develop the reduction chemistry of magnesium(I) compounds,^[13]^ we have explored the photochemical activation of β‐diketiminate substituted Mg−Mg bonded compounds, for example [{(^Dip^Nacnac)Mg}_2_] (^Dip^Nacnac=[HC(MeCNDip)_2_]^−^, Dip=2,6‐diisopropylphenyl), in arene solutions via blue or UV light irradiation.^[14]^ Our impetus for this study came from the fact that while photochemical activations of d‐block metal–metal bonds are common,^[15]^ and often lead to the generation of synthetically useful metal radical species, similar activations of stable s‐block metal‐metal bonded systems were previously unknown. We found that irradiation of dimagnesium(I) compounds in the presence of arenes does, indeed, lead to activation of the C−H bonds of benzene, toluene and xylenes, with complete regioselectivity at room temperature. These reactions gave, for example, **2 a**–**e** and the dimeric magnesium hydride complex, [{(^Dip^Nacnac)Mg(μ‐H)}_2_] (Scheme 1).^[14]^ Experimental and computational data pointed to the reactions proceeding via “Birch‐like” reduction intermediates, **1**. Here, we explore similar chemistry involving a bulky guanidinate coordinated dimagnesium(I) compound, which appears to be more reactive than its β‐diketiminato counterparts, though less regioselective in its arene C−H activation reactivity. We also provide evidence that its irradiation does lead to cleavage of the Mg−Mg bond, and generation of transient magnesium radicals.

## Results and Discussion

### Photochemically induced C−H activation of arenes

Since we reported the guanidinato‐magnesium(I) complex, [{(Priso)Mg}_2_] **3** (Priso=[Pr^*i*^_2_NC(NDip)_2_]^−^) in 2007,^[16]^ its reactivity has been poorly studied relative to that of its β‐diketiminate coordinated counterparts.^[17]^ In the initial stages of the current study we investigated its photochemical activation in neat solutions of benzene, toluene, the three isomers of xylene, and mesitylene. Like [{(^Dip^Nacnac)Mg}_2_], compound **3** is unreactive towards any of the arenes in the absence of artificial light. However, unlike [{(^Dip^Nacnac)Mg}_2_], it is also stable towards reaction with the arenes when irradiated with a blue light LED lamp (*λ*=456 nm, 50 W) at room temperature for hours. This difference likely results from the fact that **3** is colorless, whereas [{(^Dip^Nacnac)Mg}_2_] is yellow, so the former would be expected to absorb visible light more poorly.^[16]^ It should also be noted that heating solutions of **3** in the studied arenes at 60 °C overnight, did not lead to formation of any C−H activated products.

**Scheme 2:**
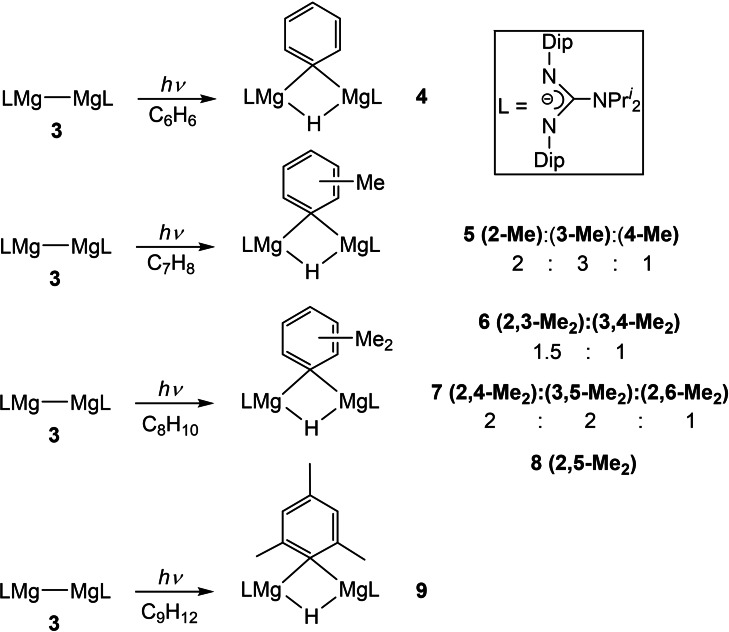
Arene C−H activation products derived from UV irradiation of solutions of **3** in toluene, xylenes, or mesitylene. The ratios of the isomers obtained are shown, and were derived from I_2_/THF quenching of the total reaction mixtures.

In contrast to the above experiments, irradiation of a benzene solution of **3** with UV light (*λ*=370 nm, 43 W lamp) in a 5 mm J. Young's NMR tube led to the near quantitative formation of the C−H activation product **4** after only 2 h (Scheme 2). When the reaction was repeated on a larger scale, compound **4** was obtained as a colorless crystalline solid in high isolated yield (80 %) after work‐up. This can be compared to the irradiation of a solution of [{(^Dip^Nacnac)Mg}_2_] in benzene by UV light, which took 48 h to generate the doubly reduced “Birch product” (**1**, *n*=0).^[14]^ Subsequent to photolysis, heating **1** (*n*=0) at 60 °C for 12 h gave **2 a** and [{(^Dip^Nacnac)Mg(μ‐H)}_2_]. This strongly suggests that **3** is significantly more reactive towards arene C−H activation than [{(^Dip^Nacnac)Mg}_2_]. Indeed, if a “Birch‐like” intermediate (cf. **1**) was generated en route to **4**, it is short‐lived, and was not observed when the reaction was followed by ^1^H NMR spectroscopy. It cannot be sure why the C−H activation of benzene by **3** gives a hydride/phenyl bridged product, while the equivalent reaction involving [{(^Dip^Nacnac)Mg}_2_] gave separate magnesium phenyl and magnesium hydride products. It is possible that in the latter case a hydride/phenyl bridged product is not sterically accessible, though a less hindered example, [{(^Mes^Nacnac)Mg}_2_(μ‐H)(μ‐Ph)] (^Mes^Nacnac=[HC(MeCNMes)_2_]^−^, Mes=mesityl), has been reported by Crimmin and co‐workers.^[11]^ It is of note that **4** is stable in benzene solutions at 60 °C for hours.

Next, UV irradiation of **3** in neat solutions of toluene, the three isomers of xylene, or mesitylene were carried out at room temperature. The reactions with toluene and xylenes were complete within 2 h and 8 h respectively, yielding the isomers of compounds **5**–**8** shown in Scheme 2. This is in contrast to the corresponding reactions with [{(^Dip^Nacnac)Mg}_2_], which took 24–96 h to reach completion, and were essentially regioselective. Another indication of the greater reactivity of **3**, is the fact that the C−H activation of mesitylene is complete after 10 h (yielding **9**), whereas [{(^Dip^Nacnac)Mg}_2_] is unreactive towards that arene under UV irradiation. Seemingly, the cost of the observed higher reactivity of **3** towards arene activation, is the loss of regioselectivity of the formed C−H activation isomeric product mixtures **5**–**7**. It is of note that work‐up of all C−H activation products consistently led to partial decomposition, and formation of the guanidine PrisoH, even when moisture was rigorously excluded. Despite this, quenching of the reaction mixtures with solutions of I_2_ in THF allowed us to estimate the relative proportions of the generated regio‐isomers, by ^1^H NMR spectroscopic and GC/MS analyses of the known aryl iodide quenched products. The approximate ratios of the products are detailed in Scheme 2 (see Supporting Information for further details).

In order to examine the chemo‐selectivity and functional group tolerance of arene C−H activations involving **3**, and for sake of comparison with similar reactivity involving [{(^Dip^Nacnac)Mg}_2_], solutions of **3** in neat fluorobenzene, anisole or *N*,*N*‐dimethylaniline were irradiated with UV light. In all cases, the magnesium(I) compound was completely consumed within two hours, but complex mixtures of inseparable products resulted. This is similar to the irradiation of [{(^Dip^Nacnac)Mg}_2_] in solutions of these arenes, except for the reaction with fluorobenzene. That led, selectively, to C−F activation of the substrate, and the near quantitative formation of biphenyl and [{(^Dip^Nacnac)Mg(μ‐F)}_2_].^[14]^

**Scheme 3:**
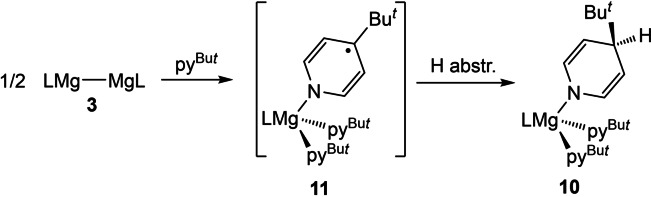
Synthesis of compound **10**.

Further to this work, the reaction of **3** with a large excess of 4‐*tert*‐butylpyridine in toluene was carried out. Our reasoning here was that, like [{(^Dip^Nacnac)Mg}_2_],^[18]^ dimagnesium(I) compound **3** could form a 2 : 1 adduct with the pyridine, and upon UV irradiation C−H activation of the coordinated pyridine could occur. However, in contrast to the formation of a stable pyridine adduct, the reaction between **3** and 4‐*tert*‐butylpyridine afforded an orange crystalline solid, which upon visual inspection appeared to contain crystals of at least two different morphologies. These could not be separated manually, or by fractional recrystallization. Only one of the crystalline materials could be characterized by X‐ray crystallography (see below), which showed it to be the magnesium(II) amide complex **10** (Scheme 3). The reaction that gave **10** proceeded rapidly in the absence of light, and at below room temperature. It is plausible that it proceeds via initial coordination of two or more pyridines to the magnesium centers of **3**, which leads to reduction of the pyridine, cleavage of its Mg−Mg bond, and formation of a transient radical species **11**. This then abstracts a hydrogen atom from the toluene solvent or excess 4‐*tert*‐butylpyridine to give **10**. This seems plausible, as we have previously shown that Mg−Mg bonds can be weakened, or cleaved, in the presence of strong Lewis bases.[^18^, ^19^] Moreover, the proposed intermediate **11**, is related to an isolated cyclic alkyl amino carbene (CAAC) complex of an amidinato magnesium fragment, [(Amid)Mg(CAAC⋅)] (Amid=[(DipN)_2_CBu^*t*^]^−^), recently reported by Harder and co‐workers.^[20]^ In that complex, the CAAC ligand is reduced to a radical anion, as is likely the case for the pyridine ligand in **11**.

Products of all the arene C−H activation reactions were characterized by NMR spectroscopy. In the case of the activated arene products where only one regio‐isomer is possible (**4**, **8** and **9**), their NMR spectra are consistent with their proposed structures. Most notably, hydride resonances were observed in the chemical shift region of ca. *δ* 4.7–5.0 ppm. Although it was not possible to assign resonances to specific regio‐isomers in the NMR spectra of the inseparable isomeric mixtures of **5**–**7**, strong evidence for the presence of all possible regio‐isomers, and their relative proportions, came from NMR spectroscopic and GC/MS analyses of the iodine quenched product mixtures, mentioned above. An analysis of the NMR spectra of the inseparable crystalline mixture that contained **10** did suggest the presence of at least two products, though the signals resulting from **10** could not be confidently assigned.

**Figure 1:**
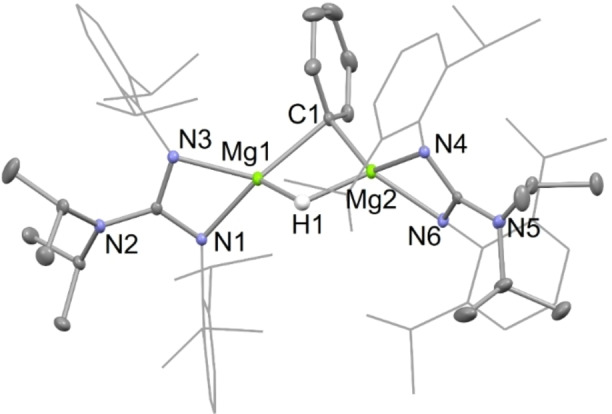
Molecular structure of **4** (20 % thermal ellipsoids are shown; hydrogen atoms, except the hydride, omitted; Dip groups shown as wireframe for clarity). Selected bond lengths (Å) and angles (°): Mg(1)−N(3) 2.0428(10), Mg(1)−N(1) 2.0593(10), Mg(1)−C(1) 2.2830(12), Mg(1)−H(1) 1.779(16), C(1)−Mg(2) 2.2335(12), Mg(2)−N(6) 2.0214(10), Mg(2)−N(4) 2.0616(10), Mg(2)−H(1) 1.830(16), N(3)−Mg(1)−N(1) 66.02(4), C(1)−Mg(1)‐H(1) 89.8(5), N(6)−Mg(2)−N(4) 66.18(4), C(1)−Mg(2)−H(1) 90.1(5).

**Figure 2:**
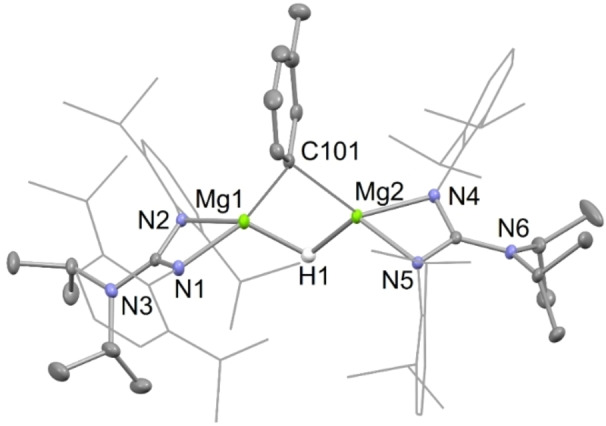
Molecular structure of **5 (3‐Me)** (20 % thermal ellipsoids are shown; hydrogen atoms, except the hydride, omitted; Dip groups shown as wireframe for clarity). Selected bond lengths (Å) and angles (°): Mg(1)−N(2) 2.0220(15), Mg(1)−N(1) 2.0625(14), Mg(1)−C(101) 2.212(4), Mg(1)−H 1.85(2), Mg(2)−N(4) 2.0419(14), Mg(2)−N(5) 2.0543(14), Mg(2)−C(101) 2.266(4), Mg(2)−H 1.83(2), N(2)−Mg(1)−N(1) 66.18(6), C(101)−Mg(1)−H 92.3(7), N(4)−Mg(2)−N(5) 66.02(6), C(101)−Mg(2)−H 91.3(7).

**Figure 3:**
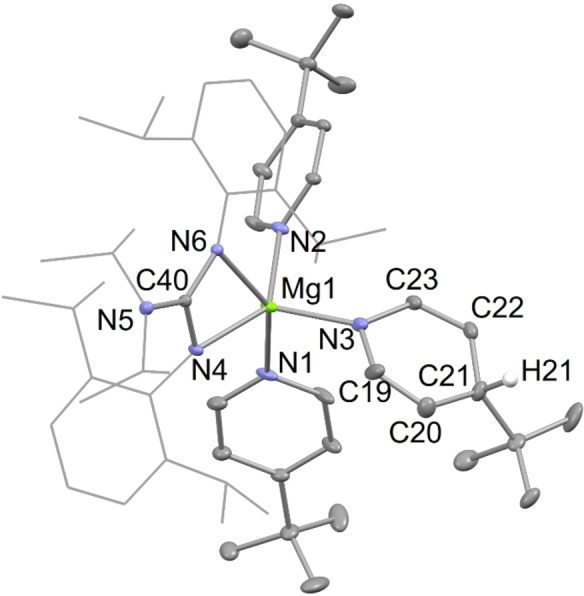
Molecular structure of **10** (20 % thermal ellipsoids are shown; hydrogen atoms, except H(21), omitted; Dip groups shown as wireframe for clarity). Selected bond lengths (Å) and angles (°): Mg(1)−N(3) 2.046(3), Mg(1)−N(4) 2.0741(18), Mg(1)−N(2) 2.165(2), Mg(1)−N(6) 2.2234(16), Mg(1)−N(1) 2.268(2), C(20)−C(21) 1.424(4), C(21)−C(22) 1.444(4), N(3)−Mg(1)−N(2) 102.12(9), N(4)−Mg(1)−N(6) 62.27(6), N(3)−Mg(1)−N(1) 96.14(10).

X‐ray diffraction studies of the activated arene/pyridine complexes, **4** and **10**, and of an approximately 1 : 2 : 1 co‐crystallized mixture of the activated toluene products, **5**, were carried out. The molecular structures of **4** and **10** are depicted in Figures 1 and 3, respectively, whereas that of the dominant *meta*‐activated toluene co‐crystallized isomer **5 (3‐Me)**, is depicted in Figure 2. It is noteworthy that the proportions of the three regio‐isomers of co‐crystallized toluene activated products in the crystal used for the diffraction experiment does not fully mirror that suggested by the iodine quenched reaction mentioned above. This situation is not unreasonable, and could conceivably result from the influence of crystal packing forces in the solid state. The hydride ligands of **4** and **5** were located from difference maps, and their positional and isotropic atomic displacement parameters freely refined. Their hydride/aryl bridged structures are very similar to each other, and to Crimmin's transition metal catalyzed benzene C−H activation product, [{(^Mes^Nacnac)Mg}_2_(μ‐H)(μ‐Ph)].^[11]^ In the solid state, complex **10** is monomeric, with its magnesium center exhibiting a distorted trigonal bipyramidal coordination geometry, including the protonated pyridine ligand in the equatorial plane. The Mg−N distance involving that ligand is considerably shorter than those associated with the datively bonded pyridines, as would be expected for what is now formally an amide substituent. Consistent with this is a loss of planarity for the reduced pyridine, and intra‐cyclic bond lengths which imply a more electronically localized structure than for the unreduced pyridines. The structure of **10** is comparable to those for compounds arising from the reductive de‐aromatization of pyridines, through reaction with β‐diketiminato magnesium hydride complexes, for example [(^Dip^Nacnac)Mg(NC_6_H_3_Me_2_‐3,5)(NC_6_H_4_Me_2_‐3,5)].^[21]^

**Figure 4:**
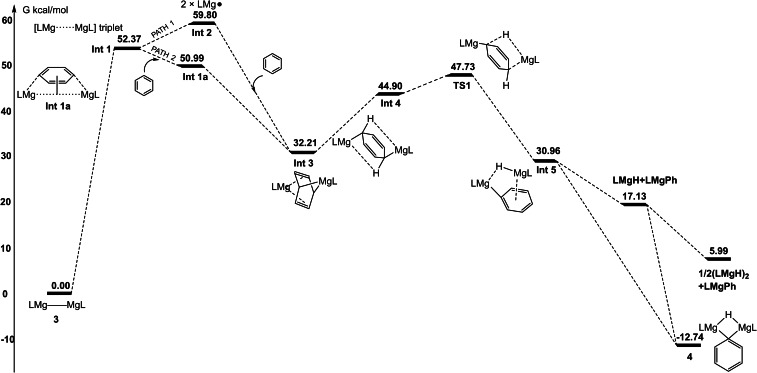
Computed Gibbs free energy profile for the reaction of [{(Priso)Mg}_2_] **3** with benzene. Energies are presented as kcal/mol.

So as to shed light on the mechanism of formation of the magnesium aryl complexes described above, and for sake of comparison with the reaction that gave **2 a**,^[14]^ the pathway of the gas phase reaction between **3** and benzene was calculated (DFT, B3PW91). Two possible radical pathways were identified, which are similar to those calculated for the formation of **2 a** (Figure 4).^[22]^ Firstly, irradiation leads to a high energy triplet state species, **Int1**, which possesses a long and weak Mg⋅⋅⋅Mg interaction. This then breaks to give two guanidinato‐magnesium(I) radicals, **Int2**, which doubly reduce benzene to give a more stable “Birch‐like” intermediate, **Int3**. A competing, but lower energy (by ca. 7 kcal/mol) pathway to **Int3** involves coordination of benzene to the photochemically activated, and elongated, Mg−Mg bond of **Int1**, to give the triplet species **Int1a**. This rearranges to give **Int3**. Although the overall energy barriers to **Int3** are very high (>50 kcal/mol) for each pathway, they are both well below the energy of the incident UV light (*λ*=370 nm≈77.3 kcal/mol). Once formed, “Birch‐like” intermediate, **Int3**, undergoes an intramolecular C−H activation process (via **Int4** and **TS1**) to give the magnesium hydride/magnesium phenyl couple **Int5**. Finally, this rearranges to give **4** as a thermodynamically stable species. It is of note that the overall energy barrier from **Int3** to **4** is 15.5 kcal/mol, which is significantly less than the highest barrier calculated from **1** (*n*=0) to **2 a** (34.2 kcal/mol).^[14]^ This may well explain why **1** (*n*=0) is an isolable species, whereas **Int3** is not. Furthermore, the lower energy of **4** compared to that of a mixture of [(Priso)MgPh] and [{(Priso)Mg(μ‐H)}_2_], discounts the latter as the final products.

### Experimental evidence for photochemically generated magnesium radical species

**Scheme 4:**
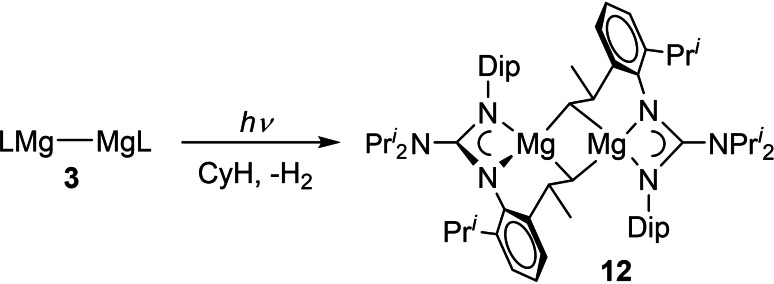
Synthesis of compound **12**.

The possibility that UV irradiation of **3** generates transient magnesium(I) radicals, which are responsible for the C−H activation of arenes described above, has been explored using synthetic and spectroscopic techniques. Firstly, cyclohexane solutions of compound **3** were irradiated with UV light over 8 h. This did not lead to C−H activation of the alkane solvent, but instead to an intramolecular C−H activation of one methyl group of the Priso ligand, and formation of an alkyl bridged dimagnesium(II) complex **12** as a colorless crystalline solid, in good isolated yield (63 %, Scheme 4). It is believed that hydrogen is also produced during the synthesis of **12**, tough its evolution was not observed during the irradiation experiment. In support of a radical pathway for the reaction are the results of DFT calculations (B3PW91) which revealed that the most thermodynamically viable pathway involves initial generation of monomeric (Priso)Mg⋅ radicals (**Int2** in Figure 4). In the absence of arenes the radicals effect the intramolecular elimination of hydrogen to give diamagnetic (Priso^−H^)Mg, which dimerizes to give **12** (see Figure S38). To investigate if compound **12** is an intermediate in the formation of **4**, the former was dissolved in benzene, and the solution irradiated with UV light. However, no reaction occurred, which essentially rules out that possibility.^[22]^

The NMR spectra for compound **12** suggest a much more symmetrical structure for the compound in solution than is seen in the solid state. That is, three apparent isopropyl methyl and two isopropyl methine signals were observed, with relative integrations of 1 : 1 : 1 and 2 : 1 respectively. No MgCH_2_ signal could be assigned in either the ^1^H or ^13^C{^1^H} NMR spectra of **12**. This observation implies that the compound is undergoing a fluxional process in solution, which could involve the rapid and reversible transfer of a proton from one methyl group on an intact Dip substituent to the methylene fragment of the deprotonated Dip^−H^ group on each ligand. Whatever the case, cooling d_8_‐toluene solutions of **12** to −60 °C led only to broadening of the resonances, implying that the fluxional process is rapid with respect to the NMR timescale, even at that temperature.

**Figure 5:**
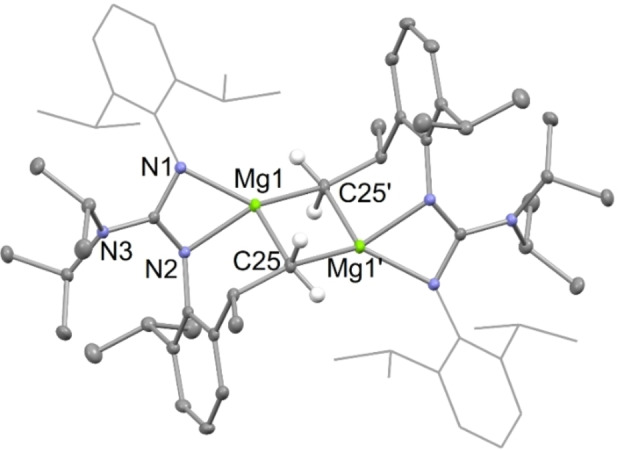
Molecular structure of **12** (20 % thermal ellipsoids are shown; hydrogen atoms, except methylene protons, omitted; Dip groups shown as wireframe for clarity). Selected bond lengths (Å) and angles (°): Mg(1)−N(1) 2.0326(16), Mg(1)−N(2) 2.0575(16), Mg(1)−C(25)’ 2.2265(19), Mg(1)−C(25) 2.304(2), N(1)−Mg(1)−N(2) 66.14(6), C(25)’−Mg(1)−C(25) 108.10(6).

The molecular structure of **12** is depicted in Figure 5. This shows the compound to exist as a centrosymmetric dimer in the solid state. Both magnesium centers have distorted tetrahedral coordination geometries, and are chelated by a deprotonated Priso ligand, the NCN backbone of which is apparently electronically delocalized. In addition, each magnesium is bridged to the other magnesium atom by two methylene fragments. The Mg−C distances in the compound [Mg(1)−C(25)=2.304(2) Å; Mg(1)−C(25)’=2.226(2) Å] highlight some asymmetry to the bridging of the alkyl fragments, which is similar to the situation for Mg−C separations in related alkyl bridged organo‐magnesium compounds, for example 2.300(3) Å and 2.257(3) Å in [(^Dip^Nacnac)Mg(μ‐Bu^*n*^)_2_].^[23]^ It is noteworthy that the C−H activation of Dip methyl groups in group 2 amide complexes has precedent with [{Ca(μ‐*κ* ^3^‐*N,N,C*‐^Dip^Nacnac^−H^)}_2_], in which both Ca centers are chelated by a deprotonated ^Dip^Nacnac ligand, the methylene group of which bridges both metal atoms.^[24]^

**Scheme 5:**
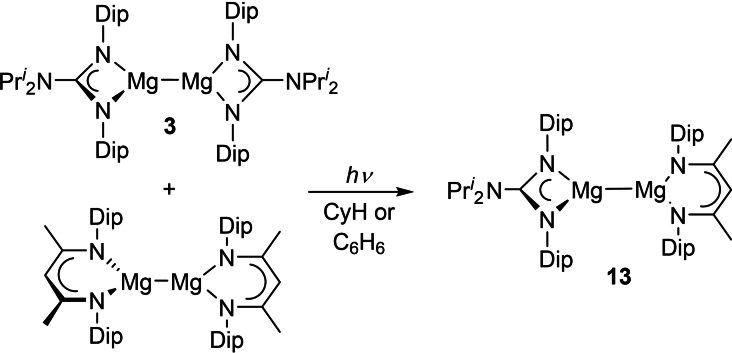
Synthesis of compound **13**.

**Figure 6:**
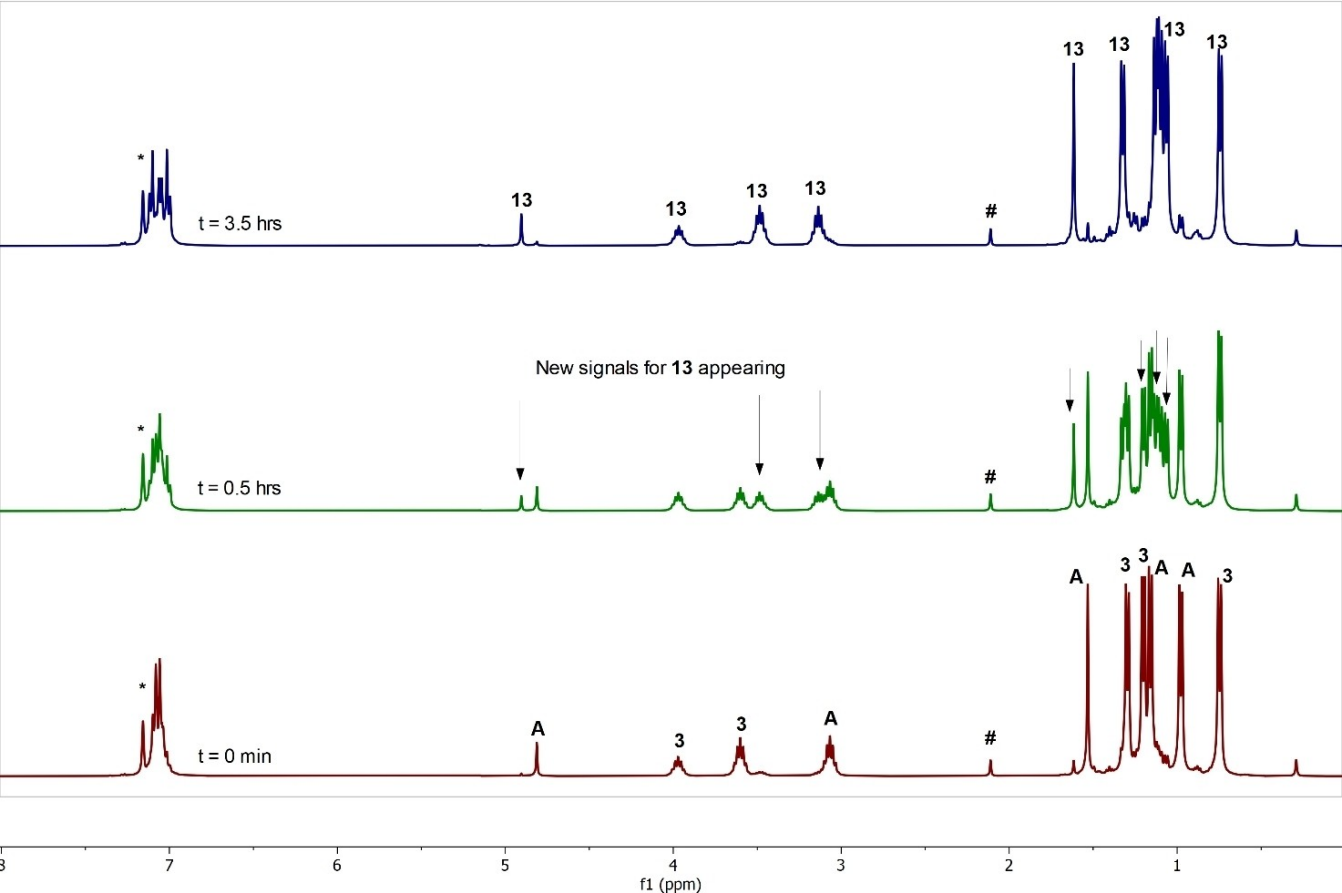
Stack plot of ^1^H NMR spectra of a 1 : 1 mixture of **3** and [{(^Dip^Nacnac)Mg}_2_] (**A**) in C_6_D_6_ which has been irradiated by blue light (*λ*=456 nm) for the time shown on each spectrum (*=signal for C_6_D_5_H; #=residual toluene).

If the irradiation of **3** does lead to homolytic cleavage of its Mg−Mg bond, it might be expected that when a 1 : 1 mixture of **3** and another dimagnesium(I) compound was irradiated, a scrambling reaction would occur, and an unsymmetrical dimagnesium(I) compound would result. To test this proposal, a number of experiments involving the irradiation of equimolar amounts of **3** and [{(^Dip^Nacnac)Mg}_2_] were carried out. The most successful experiments entailed irradiation of benzene, toluene or cyclohexane solutions of the magnesium(I) mixture with blue light. In all solvents, the scrambling reaction was very clean, and led to the near quantitative formation of the unsymmetrical dimagnesium(I) compound **13** after ca. 3 h (Scheme 5). No reaction occurred when the reaction mixture was not irradiated. When the reaction in C_6_D_6_, under blue light irradiation, was followed by ^1^H NMR spectroscopy (Figure 6), it was found that 50 % conversion to **13** occurred after approximately 1 h. Given the near quantitative formation of **13**, it is feasible that the Mg−Mg bond of the compound is significantly stronger than those in either of the reactants, and/or once formed, that Mg−Mg bond of **13** is resistant to cleavage upon irradiation. Our reasoning for this is that, if the propensity for photocleavage of the Mg−Mg bonds of **13**, **3** and [{(^Dip^Nacnac)Mg}_2_] were similar, a statistical equilibrium mixture of those compounds (1 : 1 : 2) would be expected to be reached under the photolysis conditions employed. However, that equilibrium clearly lies heavily to the right.

The outcome of the irradiation experiment is surprizing for a number of reasons. Firstly, blue light irradiations of benzene solutions of [{(^Dip^Nacnac)Mg}_2_] (in the absence of **3**) lead to the double reduction of benzene (and formation of **1**, *n*=0), while **3** is unchanged after being irradiated by blue light in benzene. Therefore, it can be tentatively concluded that the dimagnesium(I) scrambling reaction is more favorable than the reduction of benzene by [{(^Dip^Nacnac)Mg}_2_], and that cleavage and re‐formation of the Mg−Mg bond of **3** occurs when it is irradiated by blue light in benzene (in the absence of [{(^Dip^Nacnac)Mg}_2_]). With that said, compound **13** could alternatively result from reaction of {(^Dip^Nacnac)Mg}⋅ radicals with intact **3**, though computational studies indicate that such a pathway is not kinetically viable. Efforts to distinguish between these two possibilities by carrying out the irradiations in the presence of radical traps (e. g., TEMPO) were not successful, and led to intractable mixtures of products.

When cyclohexane solutions of a 1 : 1 mixture of **3** and [{(^Dip^Nacnac)Mg}_2_] are instead irradiated with UV light, a mixture of the scrambled product **13** and the C−H activation product **12** is formed. Moreover, UV irradiation of **13** in benzene leads to a mixture of benzene C−H activated product **4**, [(^Dip^Nacnac)MgPh] **2 a** and [{(^Dip^Nacnac)Mg(μ‐H)}_2_]. This reaction takes 5 days to reach completion, as opposed to the aforementioned faster formations of **4** from **3** (2 h), and of **2 a** and [{(^Dip^Nacnac)Mg(μ‐H)}_2_] from [{(^Dip^Nacnac)Mg}_2_] (48 h),^[14]^ under otherwise identical conditions. Moreover, blue light irradiation of cyclohexane solutions of **13** leads to no reaction, even after 5 days. The differences here are circumstantial evidence for the more resilient and more photo‐stable Mg−Mg bond in **13**, relative to those in **3** and [{(^Dip^Nacnac)Mg}_2_], as proposed above.

Although the formation of the unsymmetrical dimagnesium(I) compound **13** (and the other chemistry mentioned above) provides circumstantial evidence for the intermediacy of photochemically generated magnesium radical species in its formation, it should be emphasized that there are other possible routes to **13**. Indeed, previous reports have described similar scrambling reactions that occur under heating, or even rapidly at room temperature in the absence of irradiation when smaller magnesium(I) substituents are involved.[^25^, ^26^] Stasch and co‐workers have provided evidence that dimagnesium(I) scrambling reactions can occur via an associative mechanism, which involves exchange of anionic β‐diketiminate or diiminophosphinate ligands between magnesium centres.^[26]^ Such a mechanism cannot be completely ruled out for the formation of **13**.

**Figure 7:**
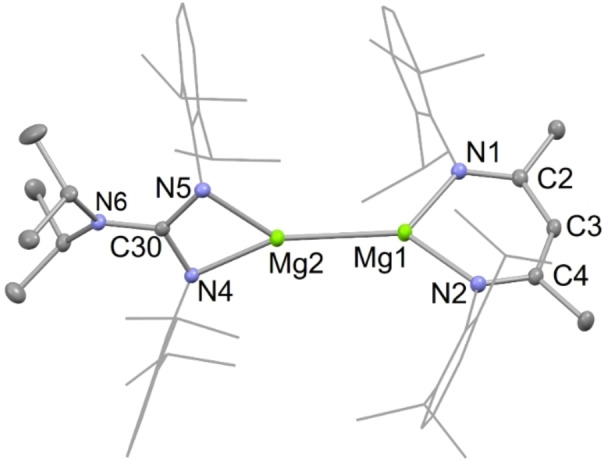
Molecular structure of **13** (20 % thermal ellipsoids are shown; hydrogen atoms omitted; Dip groups shown as wireframe for clarity). Selected bond lengths (Å) and angles (°): Mg(1)−N(2) 2.0365(11), Mg(1)−N(1) 2.0436(12), Mg(1)−Mg(2) 2.7935(6), Mg(2)−N(4) 2.0586(11), Mg(2)−N(5) 2.0692(11), N(2)−Mg(1)−N(1) 93.49(5), N(4)−Mg(2)−N(5) 65.46(4).

The solution state NMR spectra for **13** are consistent with the solid‐state structure of the compound, which is depicted in Figure 7. In the solid state, the coordination geometry of both magnesium centers in **13** is distorted trigonal planar, with the dihedral angle between the two least squares heterocyclic planes being 69.8°. The Mg−Mg bond length is 2.7935(6) Å, which is shorter than those in all of the approximately 40 previously reported dimagnesium(I) compounds.^[27]^ This is, perhaps, indicative of the strength of the bond, which was also apparent from the results of the scrambling reaction that generated it, and its slow reaction with benzene, as discussed above.

DFT calculations on **13** in the gas phase (B3PW91) showed it to optimize with a geometry similar to that in the solid state. As has been calculated for other neutral dimagnesium(I) compounds,[^13^, ^16^] its HOMO has a high degree of Mg−Mg σ‐bond character, which is predominantly derived from overlap of Mg s‐orbitals. The lowest unoccupied metal centered molecular orbital is the LUMO+1, which resembles a “twisted” Mg−Mg π‐bonding orbital (see Figure S37; HOMO‐LUMO+1 energy difference=4.19 eV). Although the Mg_2_^2+^ core of **13** is unsymmetrically substituted, the Mg−Mg bond is only slightly polarized, with ca. 52.4 % of electron density associated with the β‐diketiminate coordinated magnesium center. This situation is comparable to previously reported calculations on unsymmetrical dimagnesium(I) compounds.[^19^, ^28^] In line with experimental observations, the Mg−Mg bond dissociation energy for **13** (BDE=72.65 kcal/mol) is greater than for both **3** (BDE=70.14 kcal/mol) and [{(^Dip^Nacnac)Mg}_2_](BDE=66.02 kcal/mol).

**Figure 8:**
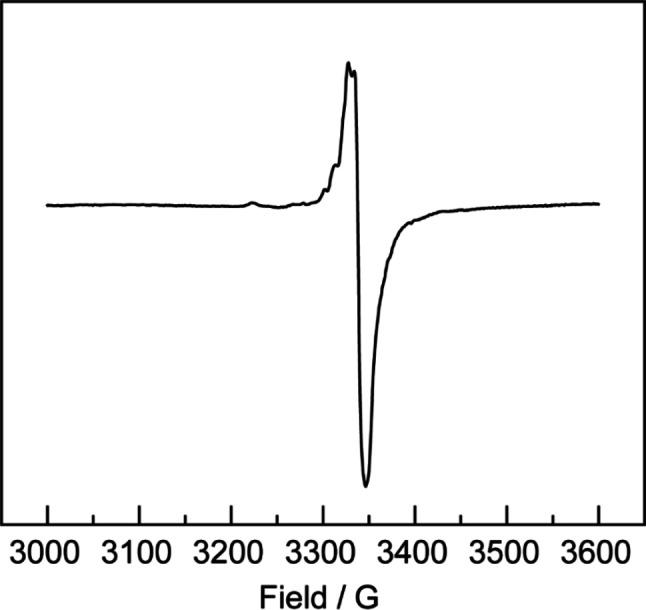
EPR spectrum of a solution of **3** in a 3/1 mixture of 2,2‐dimethylbutane and *tert*‐butylbenzene, which has been irradiated for 1 h with UV light (*λ*=335 nm) at 77 K.

In order to gain further evidence for the photochemical generation of radical species from **3**, a solution of the compound (1.0 mg) in a 3 : 1 mixture of 2,2‐dimethylbutane/tert‐butylbenzene (0.15 mL) was cooled to 77 K, and the resulting glass^[29]^ irradiated for 1 h with UV light (*λ*_max_=335 nm, 100 W Hg lamp). The X‐band EPR spectrum of the sample was then recorded at 77 K, and is depicted in Figure 8. The spectrum exhibits a broad signal centered at 3335 G, which has poorly defined hyperfine structure. This is comparable to the broad, largely featureless signal (centered at 3362 G) observed by Schnöckel and co‐workers in the EPR spectrum of a toluene solution of Mg^I^Br radicals at 77 K.^[30]^ So as to rule out the possibility that the EPR signal seen in the spectrum of irradiated **3** originates from traces of its known decomposition or disproportionation products, PrisoH and [Mg(Priso)_2_], solutions of those compounds were similarly irradiated, and their EPR spectra obtained. In neither case was any significant signal observed. The absence of a half‐field signal in the spectrum implies that the radical that gave rise to the dominant resonance in the spectrum is not a triplet species.

Considering the broad nature of the EPR signal for irradiated **3**, little success was had obtaining a suitable simulation. What is clear, is that the observed signal is a composite of a broad dominant signal, and a much less intense signal (ca. 40 : 1 ratio), the latter of which has hyperfine structure, and has been simulated. This signal potentially arises from the presence of a small amount of ligand radical, Priso**⋅** (see Supporting Information for full details). With respect to the EPR spectrum, it is worth noting that DFT calculations on the monomeric (Priso)Mg⋅ radical (**Int2** in Figure 4) showed it to optimize with the ligand *N,N*‐chelating the magnesium centre. The calculations also show the SOMO to be of high s‐character, and largely associated with the Mg‐center (Figure S36). This might suggest that a significant hyperfine coupling of the unpaired electron to the ^25^Mg nucleus (I=5/2, 10 % natural abundance) would be observed in the experimental EPR spectrum of irradiated **3**. However, simulations of the spectrum using a broad range of ^25^Mg hfc values (A(^25^Mg) 1–100 G) all gave broad central signals with little fine structure.^[31]^ As a result, and as was the case with the spectrum of Schnöckel's Mg^I^Br radical, little can be concluded about the electronic structure of the radical from the EPR spectrum. Despite this, it is worthy of mention that the EPR spectra of two related amido‐magnesium radical complexes, in which the unpaired electron is ligand based, have been recently reported, *viz*. Harder's [(Amid)Mg(CAAC⋅)]^[20]^ and Tan's [Mg(THF)_2_(Carbazol⋅)] (Carbozol=3,6‐di‐*tert*‐butyl‐1,8‐bis(10‐phenylanthracen‐9‐yl)carbazolyl).^[32]^ A very small ^25^Mg hfc (1.6 G) was observed for the former, whereas none was recorded for the latter.

## Conclusion

In summary, the irradiation of solutions of a guanidinate coordinated dimagnesium(I) compound **3** in various arene solvents has been explored, and compared with similar reactions reported for β‐diketiminate coordinated counterparts of **3**. In no case does a reaction occur under blue light irradiation. In contrast, UV irradiation of solutions of **3** in benzene, toluene, the three isomers of xylene, and mesitylene, all lead to C−H activation of the arene and good yields of magnesium aryl products. These reactions are considerably faster than those involving β‐diketiminato dimagnesium(I) compounds, but they show little regioselectivity. DFT calculations suggest they proceed via photochemical cleavage or elongation of the Mg−Mg bond of the magnesium reactant, and reduction of the arene substrate by generated magnesium(I) radicals, prior to C−H activation processes occurring. A number of experiments have provided circumstantial evidence that the UV light irradiation of solutions of **3** leads to transient magnesium radical species. Firstly, irradiation of cyclohexane solutions of **3** results in an intramolecular aliphatic C−H activation process, and formation of a magnesium alkyl species, **12**. Secondly, irradiation of a 1 : 1 mixture of **3** and a β‐diketiminato dimagnesium(I) compound leads to a “scrambling” reaction, and the near quantitative formation of an unsymmetrical dimagnesium(I) compound, **13**. Finally, the EPR spectrum of a glassed solution of irradiated **3** at 77 K shows the presence of a radical species, though it cannot definitively be concluded that this arises from the magnesium(I) radical, (Priso)Mg⋅. We continue to explore the use of photochemically activated dimagnesium(I) compounds as reagents for organic transformations that normally require late transition metal complexes to proceed.

## Experimental Section

Experimental procedures and characterization data for all new compounds, full details of the computational studies, crystal data, and details of data collections and refinements are available in the Supporting Information.

Deposition Number(s) 2182705 [for **4**⋅(benzene)_0.5_], 2182703 [for **5**⋅(toluene)_0.5_], 2182702 [for **10**⋅(benzene)_0.25_], 2182701 [for **12**⋅(hexane)], 2182704 [for **13**⋅(hexane)_0.5_] contain(s) the supplementary crystallographic data for this paper. These data are provided free of charge by the joint Cambridge Crystallographic Data Centre and Fachinformationszentrum Karlsruhe Access Structures service.

## Conflict of interest

The authors declare no conflict of interest.

1

## Supporting information

As a service to our authors and readers, this journal provides supporting information supplied by the authors. Such materials are peer reviewed and may be re‐organized for online delivery, but are not copy‐edited or typeset. Technical support issues arising from supporting information (other than missing files) should be addressed to the authors.

Supporting Information

## Data Availability

The data that support the findings of this study are available in the supplementary material of this article.
