# Supplementary material for: C−H Activation of Inert Arenes using a Photochemically Activated Guanidinato‐Magnesium(I) Compound
Source: Chemistry. 2022 Sep 26;28(65):e202202103. doi: 10.1002/chem.202202103 (PMC9828268; doi:10.1002/chem.202202103)
Supplement: Supplementary file 1 — Supporting Information [file CHEM-28-0-s001.pdf]

Chemistry–A European Journal

Supporting Information

C–H Activation of Inert Arenes using a Photochemically Activated Guanidinato-Magnesium(I) Compound

Jeremy C. Mullins, K. Yuvaraj, Yixiao Jiang, Gerard P. Van Trieste, III, Asim Maity, David C. Powers,* and Cameron Jones*

Contents

**1. Syntheses and spectra**	**S2**
**2. X-Ray crystallography**	**S27**
**3. EPR spectroscopic studies**	**S30**
**4. Computational studies**	**S32**
**5. References**	**S75**

1. Syntheses and spectra

General considerations

All manipulations were carried out using standard Schlenk and glove box techniques under an atmosphere of high purity dinitrogen. Benzene, toluene and hexane were distilled over molten potassium, whilst pentane was distilled over Na/K alloy (50:50). Xylenes and mesitylene were died over 3Å molecular sieves. ^{1}H and ^{13}C{^{1}H} NMR spectra were recorded on Bruker Avance III 400 or Bruker Avance III 600 spectrometers and were referenced to the resonances of the solvent used. FTIR spectra were collected for solid samples or Nujol mulls on an Agilent Cary 630 attenuated total reflectance (ATR) spectrometer. Microanalyses were carried out using a PerkinElmer-2400 CHNS/O Series II System. GC/MS analyses were conducted on an Agilent 6890 GC coupled to a 5973 MS system with an EI source. A 1µL injection volume of the test compound was used in conjunction with a 100:1 split ratio and a He carrier gas with a constant flow of 1 mL/min. Analytes were separated on an Agilent DB-5MS 30m column with a film thickness of 0.25 µm and a diameter of 0.25 mm on a temperature gradient starting at 50 °C for 3 minutes and ramping to 300 °C at a rate of 12 °C per minute. A final hold time of 5 minutes was employed. The MS was operated in scan mode with a mass range of m/z 30-550 units. Irradiations were carried out using either Kessil PR160L blue (λ = 456 nm, 50W) or UV (λ = 370 nm, 43W) light LED lamps, with the reaction vessel placed approximately 2 cm from the light source, whilst being cooled by an external fan. Melting points were determined in sealed glass capillaries under dinitrogen and are uncorrected. The starting materials [{(Priso)Mg}$_2$],[1] [{(^{Dip}Nacnac)Mg}$_2$][2] and PrisoH[3] were prepared according to the literature procedures.

[{(Priso)Mg}$_2$(µ-H)(µ-C_6H_5)] (4).

NMR scale: [{(Priso)Mg}$_2$] **3** (10 mg, 0.010 mmol) was dissolved in 0.5 mL of C_6D_6 in a J. Young's NMR tube at room temperature. The sample was irradiated with UV light and the progress of the reaction monitored by ^{1}H NMR spectroscopy. After 2h, the near quantitative formation of **4** was observed.

Preparatory scale: [{(Priso)Mg}$_2$] **3** (100 mg, 0.103 mmol) was suspended in 4 mL of benzene in a 10 mm J. Young's NMR tube. The sample was irradiated with UV light for 6h. After this, the colorless solution was filtered, concentrated to *ca.* 1 mL, then placed at 4 °C for 1 d. Volatiles were then removed *in vacuo,* and the residue dissolved in *ca.* 4 mL of hexane, and then filtered. The filtrate was concentrated to *ca.* 2 mL, then placed at -30 °C for 3 d, yielding colorless crystals of **4** (86 mg, 80 %). M.p: >264 °C; ^{1}H NMR (400 MHz, C_6D_6, 298 K) δ 0.78 (d, J = 6.9 Hz, 24H, CH(CH_3)$_2$), 1.01

(d, J = 6.7 Hz, 24H, CH(CH_3)$_2$), 1.30 (d, J = 6.8 Hz, 24H, NCH(CH_3)$_2$), 3.54–3.62 (m, 8H, CH(CH$_3$)$_2$), 3.9 –3.98 (m, 4H, NCH(CH$_3$)$_2$), 4.68 (s, 1H, Mg(µ-H)), 6.82–7.67 (m, 17H, Ar-H); $^{13}C\{^1H\}$ NMR (101 MHz, C_6D_6, 298 K) δ 22.9 (NCH(CH$_3$)$_2$), 23.8, 25.8 (CH(CH$_3$)$_2$), 27.9 (CH(CH$_3$)$_2$), 49.8 (NCH(CH$_3$)$_2$), 123.2, 123.4, 123.5, 142.8, 142.9, 143.2, 143.8, 144.0 (Ar-C), 166.9 (CN$_3$); IR v/cm^{-1} (Nujol): 1609 (s), 1584 (s), 1179 (w), 1153 (w), 1126 (m), 1111 (m), 1042 (m), 933 (w), 799 (m), 756 (s), 712 (m); a satisfactory micro-analysis could not obtained as the compound co-crystallizes with a small amount of PrisoH, consistently generated during reaction work-up, which could not be removed after repeated recrystallizations.

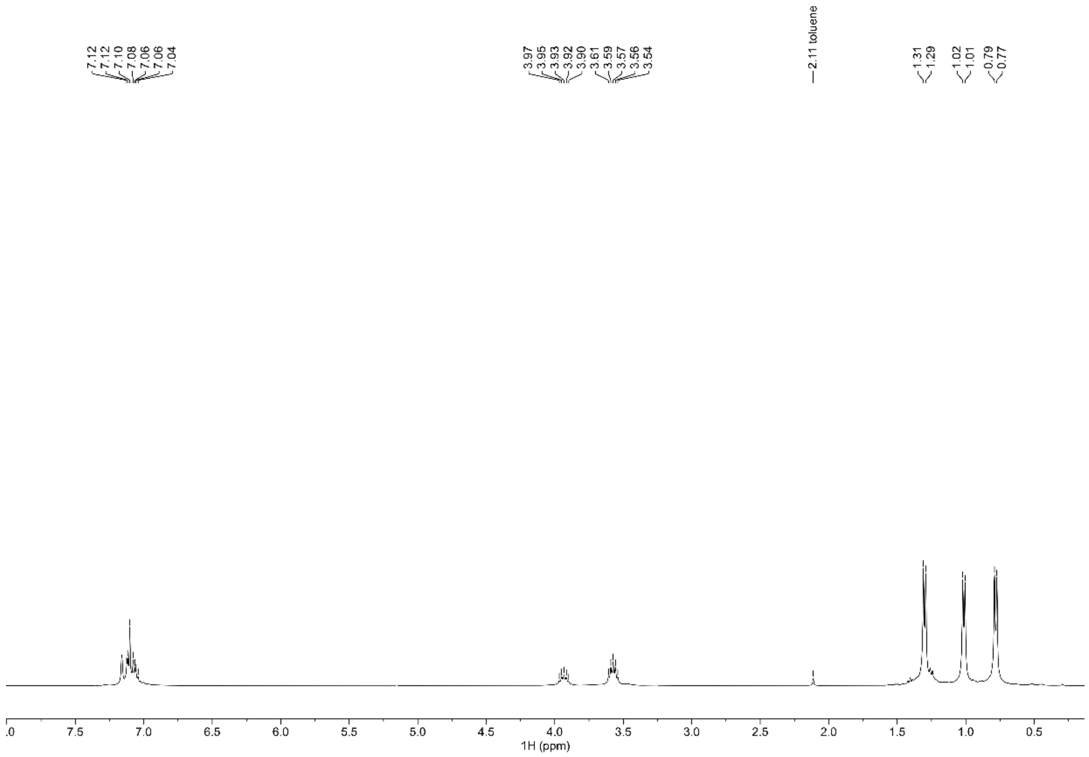

Figure S1. ^{1}H NMR spectrum (400 MHz, 298 K, C_6D_6) of **4**, generated *in situ* by irradiating a C_6D_6 solution of **3** with UV light for 2 hours.

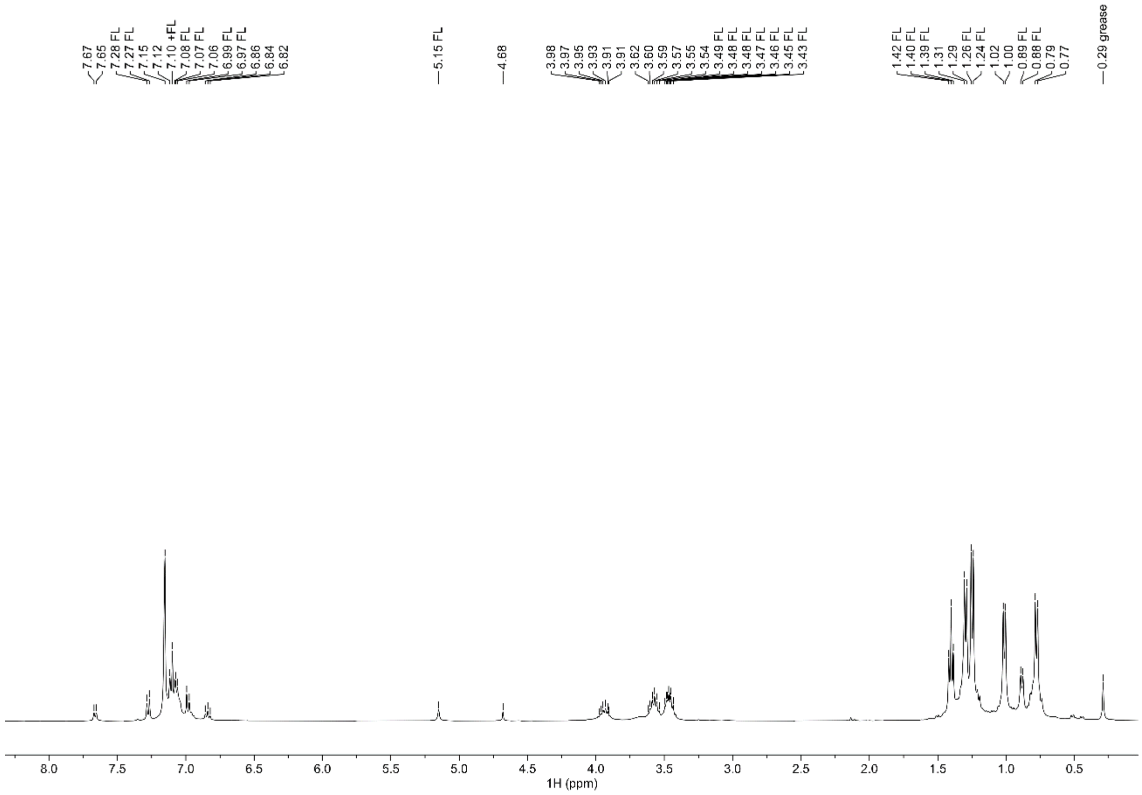

Figure S2. ^{1}H NMR spectrum (400 MHz, 298 K, C_6D_6) of **4**, after work-up and rescrystallization (FL = PrisoH). This shows the generation of PrisoH during the work-up procedure, which occurs even when moisture is rigourously excluded.

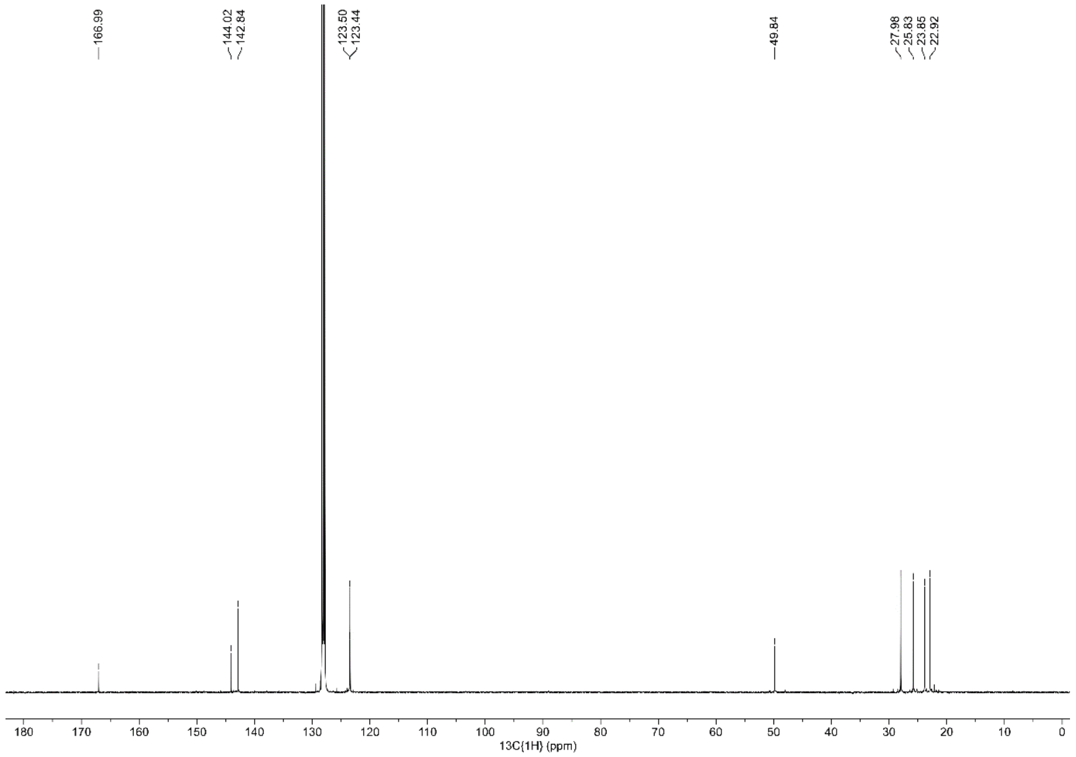

Figure S3. $^{13}C\{^{1}H\}$ NMR spectrum (101 MHz, 298 K, C_6D_6) of **4**, generated *in situ* by irradiating a C_6D_6 solution of **3** with UV light for 2 hours.

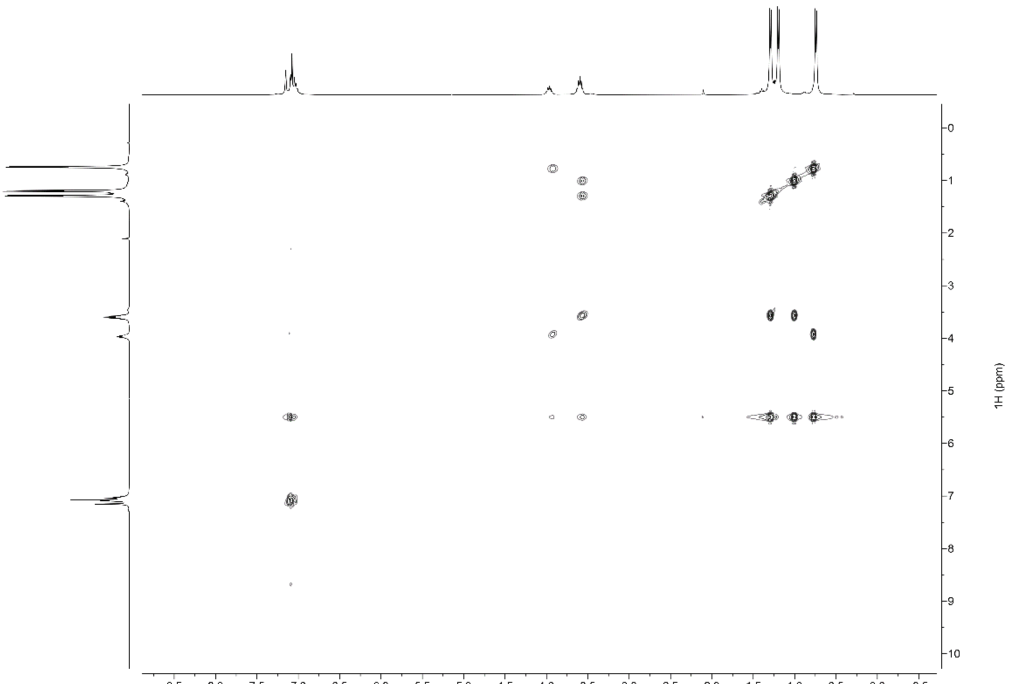

Figure S4. ^{1}H-COSY NMR spectrum (400 MHz, 298 K, C_6D_6) of **4**, generated *in situ* by irradiating a C_6D_6 solution of **3** with UV light for 2 hours.

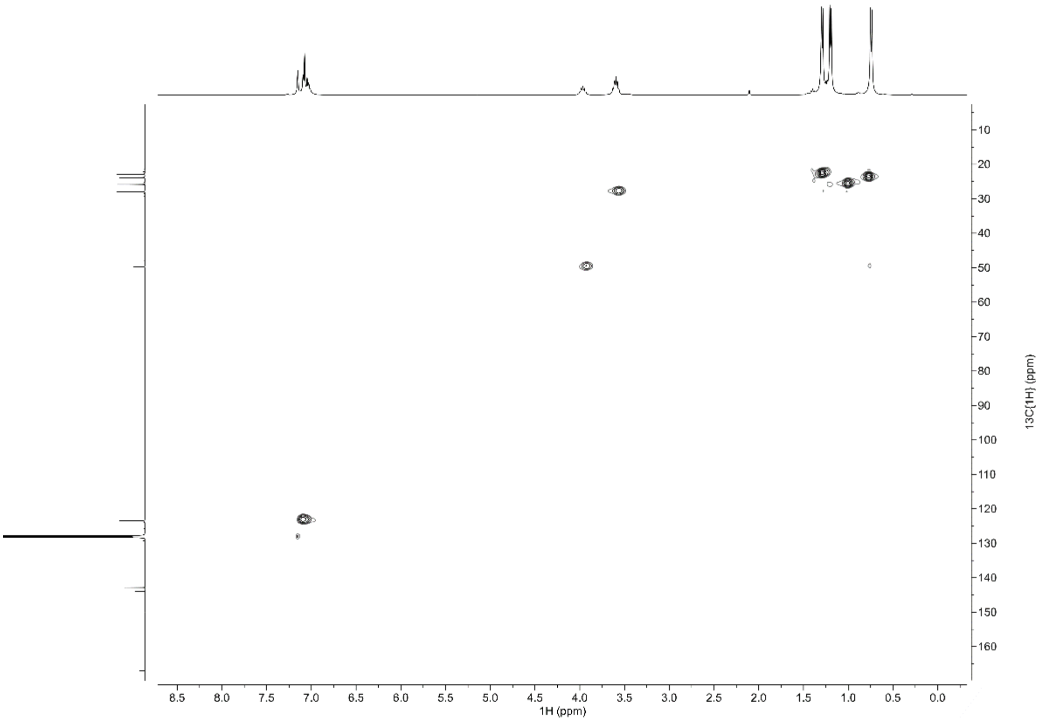

Figure S5. HMQC spectrum (^{1}H: 400 MHz; ^{13}C: 101 MHz, 298 K, C_6D_6) of **4**, generated *in situ* by irradiating a C_6D_6 solution of **3** with UV light for 2 hours.

[{(Priso)Mg}$_2$(μ-H)(μ-C_6H_4Me)] (5).

NMR scale: Compound **3** (10 mg, 0.010 mmol) was dissolved in 0.5 mL of toluene-d_8 in a J. Young's NMR tube at room temperature. The sample was irradiated with UV light, and the progress of the reaction monitored by ^{1}H NMR spectroscopy. After 2h, the formation of three isomers of **5** was observed with a high yield of the isomer mixture.

Preparatory scale: Compound **3** (100 mg, 0.103 mmol) was suspended in 4 mL of toluene in a 10 mm J. Young's NMR tube. The sample was irradiated with UV light for 6h. Volatiles were then removed *in vacuo*, and the residue redissolved in *ca.* 4 mL of hexane, and filtered. The filtrate was concentrated to *ca.* 2 mL, then placed at -30 °C for 2 d. After this, colorless crystals of a mixture of the three isomers of **5** had deposited (85 mg, 78 %). The ^{1}H NMR spectroscopic signals associated with each isomer, and their relative integrations, could not be confidentally assigned. M.p: 131-136 °C; ^{1}H NMR (400 MHz, toluene-d_8, 298 K): δ 0.80 (overlapping d, CH(CH_3)$_2$), 1.02 (overlapping d, CH(CH_3)$_2$), 1.30 (overlapping d, NCH(CH_3)$_2$), 1.79, 1.89, 1.97 (*o*- or *m*- or *p*-tolyl-CH_3), 3.55–3.61 (m, C*H*(CH$_3$)$_2$), 3.92–4.02 (m, NC*H*(CH$_3$)$_2$), 4.68, 4.70, 4.77 (s, Mg(μ-*H*)), 6.67–7.46 (m, Ar-*H*); ^{13}C{^{1}H} NMR (101 MHz, toluene-d_8, 298 K) δ 23.2, 23.3, 23.5 (NCH(*C*H$_3$)$_2$), 24.1, 24.3, 24.4, 26.0, 26.11, 26.14 (CH(*C*H$_3$)$_2$), 28.32 (*C*H(CH$_3$)$_2$), 28.36 (br.s, two resonances signals are overlapping for *C*H(CH$_3$)$_2$), 50.1, 50.2, 50.4 (N*C*H(CH$_3$)$_2$), 123.7, 123.85, 123.88, 143.0, 143.1, 143.2, 144.4, 144.54, 144.59 (Ar-*C*), (some Ar-*C*H and Ar-*C*H$_3$ are with toluene-d_8 resonances) 167.0, 167.3, 167.8 (*C*N$_3$);

IR ν/cm^{-1} (Nujol): 1607 (s), 1580 (s), 1259 (s), 1184 (s), 1154 (s), 1094 (s), 1015 (s), 932 (s), 863 (s), 797 (s), 767 (s), 714 (s); a satisfactory micro analysis could not obtained as the compound co-crystallizes with a small amount of PrisoH, consistently generated during reaction work-up, which could not be removed after repeated recrystallizations.

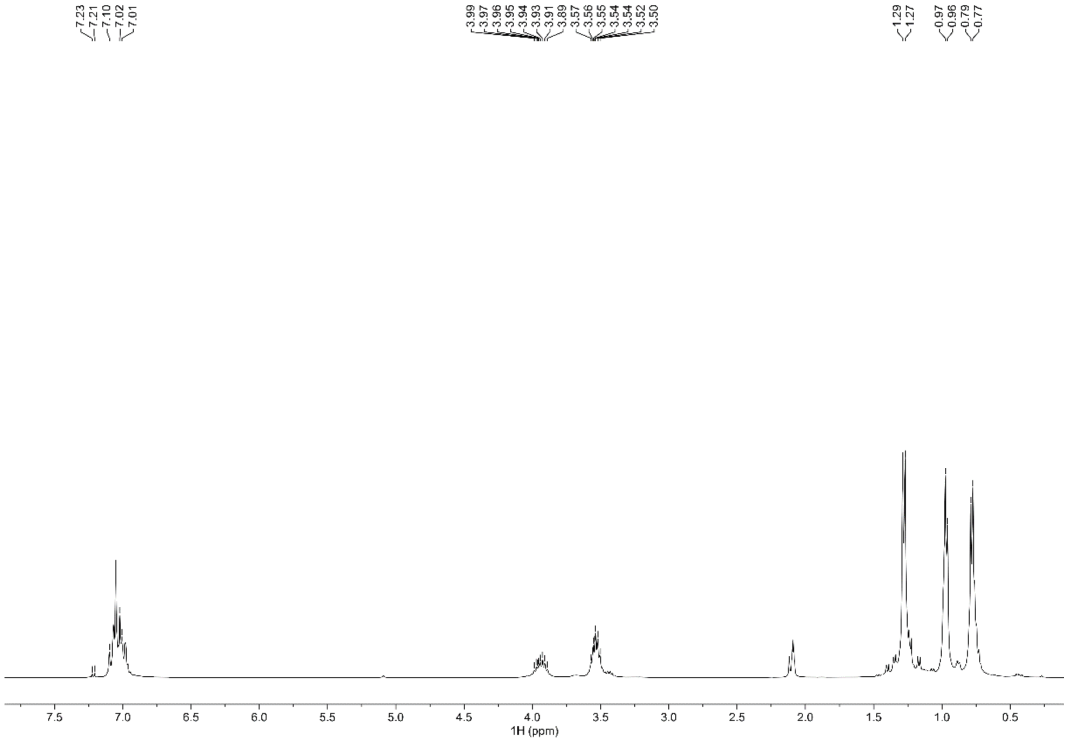

Figure S6. ^{1}H NMR spectrum (400 MHz, 298 K, toluene-d_8) of the isomeric mixture of **5**, generated *in situ* by irradiating a toluene-d_8 solution of **3** with UV light for 2 hours.

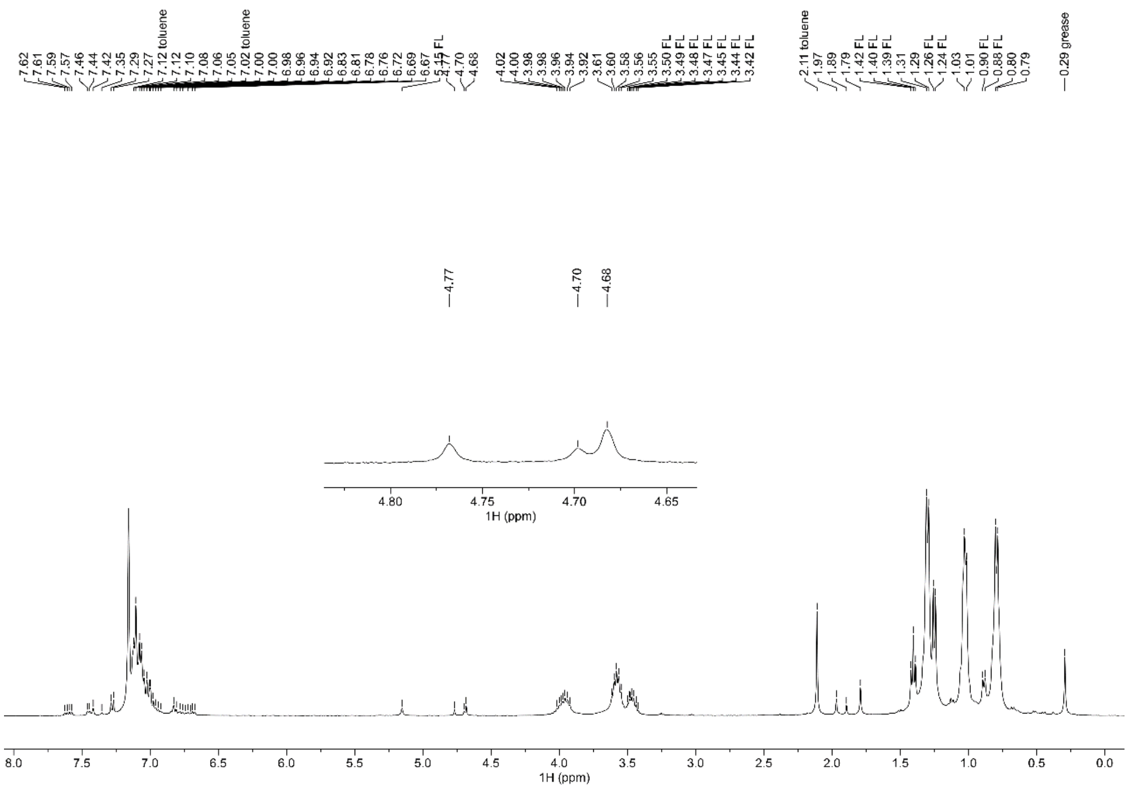

Figure S7. ^{1}H NMR spectrum (400 MHz, 298 K, C_6D_6) of the isomeric mixture of **5**, after work-up and recrystallization (hydride resonances inset, FL = PrisoH). This shows the generation of PrisoH during the work-up procedure, which occurs even when moisture is rigourously excluded.

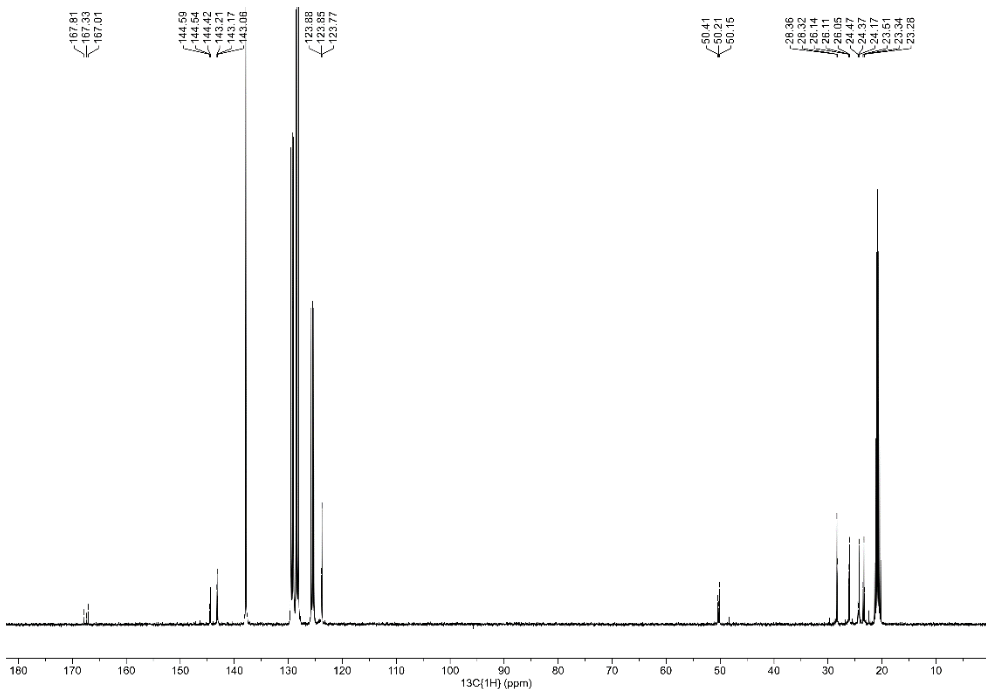

Figure S8. $^{13}C\{^{1}H\}$ NMR spectrum (101 MHz, 298 K, C_6D_6) of the isomeric mixture of **5**, generated *in situ* by irradiating a toluene-d_8 solution of **3** with UV light for 2 hours.

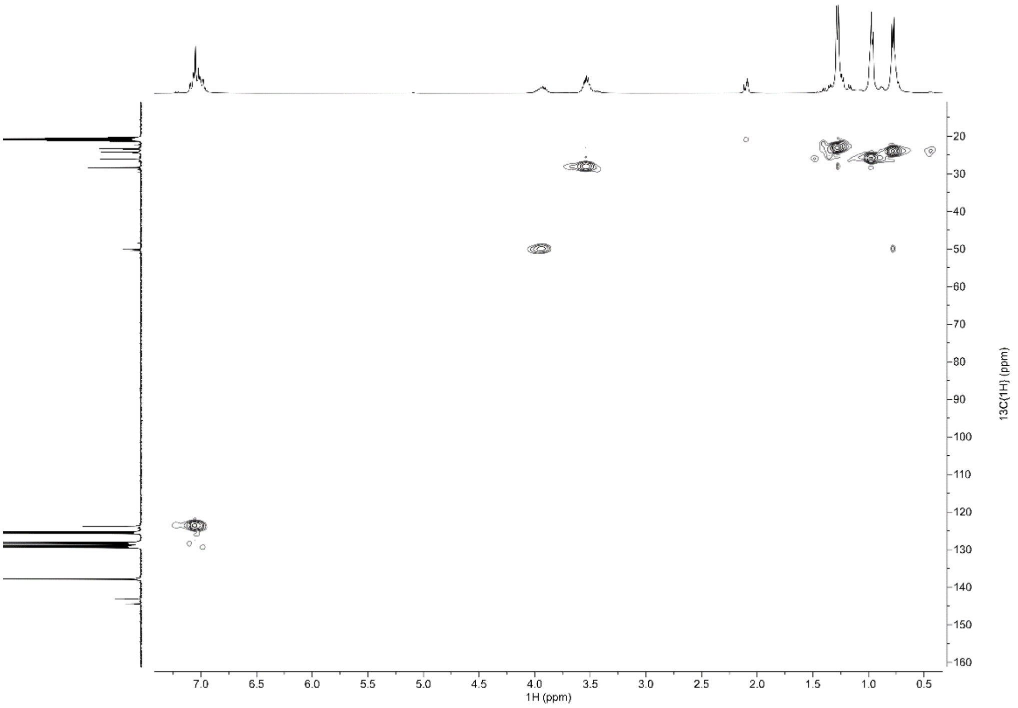

Figure S9. HMQC spectrum (^{1}H: 400 MHz; ^{13}C: 101 MHz, 298 K, C_6D_6) of the isomeric mixture of **5**, generated *in situ* by irradiating a toluene-d_8 solution of **3** with UV light for 2 hours.

[{(Priso)Mg}$_2$(μ-H)(μ-*o*-$C_6H_3Me_2$)] (6).

Compound **3** (60 mg, 0.062 mmol) was suspended in 4 mL of *o*-xylene in a 10 mm J. Young's NMR tube. The sample was irradiated with UV light for 8h. Volatiles were then removed *in vacuo*, the residue redissolved in *ca.* 4 mL of hexane, filtered and the filtrate dried under vacuum, yielding **6** as a pale-yellow powder (55 mg). All attempts to recrystallize the isomeric mixture of **6** failed. M.p: 171–176°C; the ^{1}H NMR spectroscopic signals associated with each isomer, and their relative integrations, could not be confidentally assigned, and the spectrum contains unknown by-products. The presence of the two isomers of **6** is inferred by the results of the I_2/THF quenching experiment detailed below. ^{1}H NMR (400 MHz, C_6D_6, 298 K): δ 0.76–0.83 (overlapping d, CH(C*H*$_3$)$_2$), 1.03 (overlapping d, CH(C*H*$_3$)$_2$), 1.31 (overlapping d, NCH(C*H*$_3$)$_2$), 1.73, 1.83, 1.87, 1.89 (Ar-C*H*$_3$), 3.54–3.62 (m, C*H*(CH$_3$)$_2$), 3.95–4.03 (m, NC*H*(CH$_3$)$_2$), 4.70, 4.80 (s, Mg(μ-*H*)), 6.68–7.47 (m, Ar-*H*); ^{13}C NMR (101 MHz, C_6D_6, 298 K) δ 22.6, 22.7, 22.8 (Ar-CH$_3$), 23.02, 23.09, 23.1, 23.7 (NCH(*C*H$_3$)$_2$), 23.9, 24.0, 24.2 (CH(*C*H$_3$)$_2$), 25.6 (Ar-*C*H$_3$), 25.70, 25.76, 25.8, 26.3, 26.4 (CH(*C*H$_3$)$_2$), 26.7, 26.8, 27.9, 28.0 (*C*H(CH$_3$)$_2$), 49.81, 49.89, 49.9, 50.0 (N*C*H(CH$_3$)$_2$), 123.43, 123.46, 123.53, 123.56, 123.6, 127.5, 129.1, 131.0, 131.2, 132.5, 134.4, 134.6, 135.8, 136.6, 137.1, 138.3, 140.5, 142.4, 142.67, 142.69, 142.7, 142.8, 142.9, 143.2, 143.5, 144.14, 144.16, 144.18, 144.3, 145.2, 145.3, 150.7, 152.9 (Ar-*C*), 167.36, 167.39 (*C*N$_3$); IR ν/cm^{-1} (Nujol): 1609 (s), 1582 (s), 1246 (s), 1185 (s), 1155 (s), 1109 (s), 1046 (s), 1002 (s), 933 (s), 872 (s), 829 (s), 799 (s), 829 (s), 799 (s), 756 (s), 723 (s); a satisfactory

micro analysis could not obtained as the product could not be crystallized, and contains PrisoH and small amounts of other by-products.

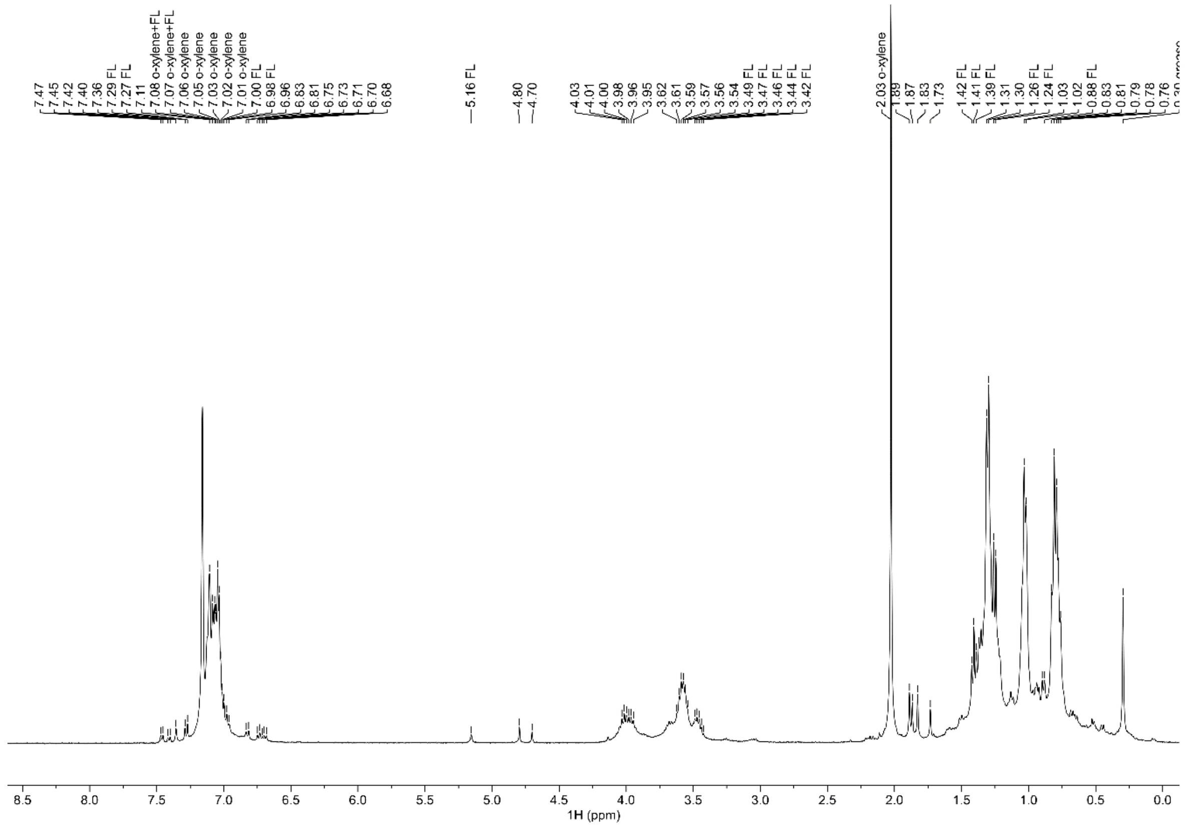

Figure S10. ^{1}H NMR spectrum (400 MHz, 298 K, C_6D_6) of an isomeric mixture of **6**, and reaction by-products (FL = PrisoH).

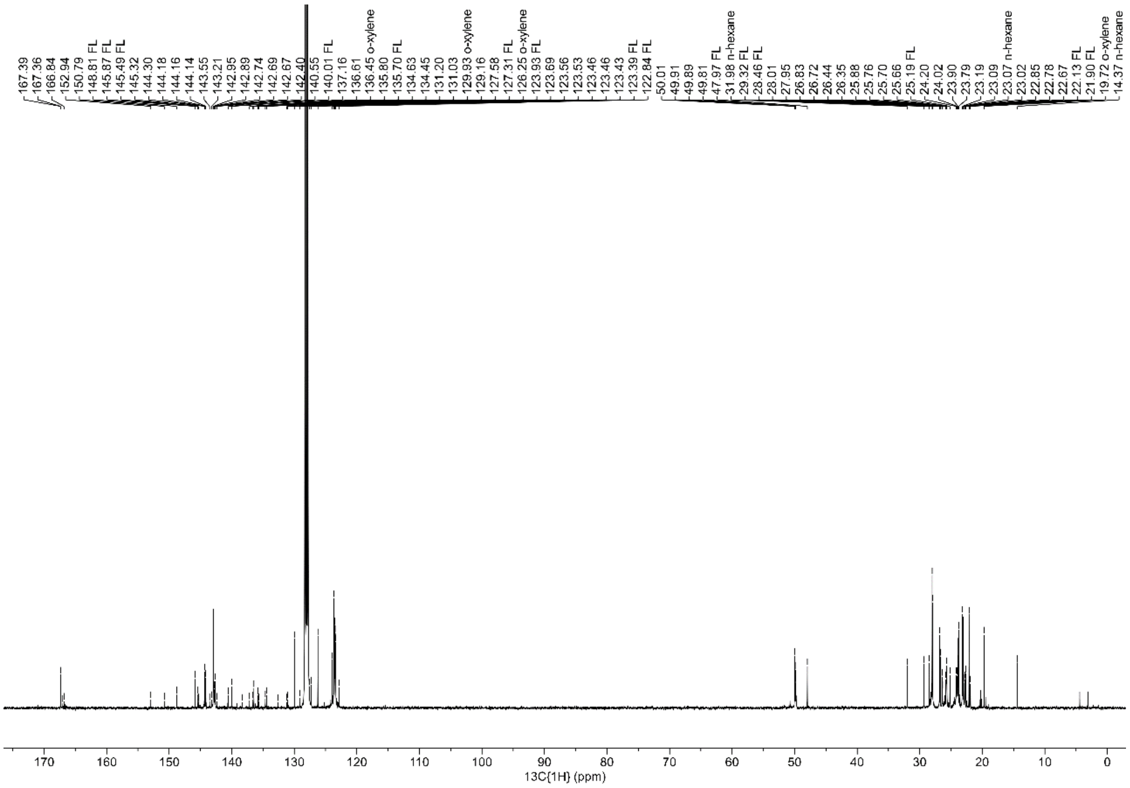

Figure S11. $^{13}C\{^1H\}$ NMR spectrum (101 MHz, 298 K, C_6D_6) of an isomeric mixture of **6**, and reaction by-products (FL = PrisoH).

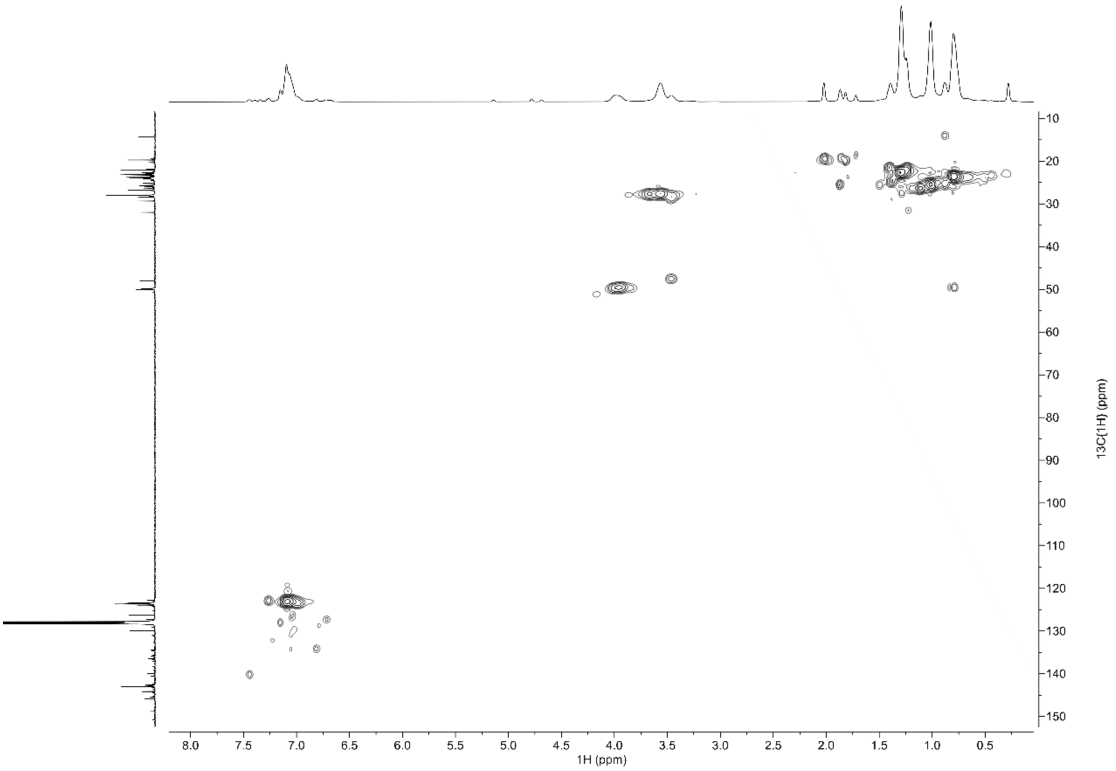

Figure S12. HMQC spectrum (^1H: 400 MHz; ^{13}C: 101 MHz, 298 K, C_6D_6) of an isomeric mixture of **6**, and reaction by-products.

[{(Priso)Mg}$_2$(μ-H)(μ-*m*-$C_6H_3Me_2$)] (7).

Compound **3** (50 mg, 0.051 mmol) was suspended in 4 mL of *m*-xylene in a 10 mm J. Young's NMR tube. The sample was irradiated with UV light for 8h. Volatiles were then removed *in vacuo*, the residue redissolved in *ca.* 4 mL of hexane, filtered and the filtrate dried under vacuum, yielding **7** as a pale-yellow powder (46 mg). All attempts to recrystallize the isomeric mixture of **7** failed. M.p: > 264 °C; the ^{1}H NMR spectroscopic signals associated with each isomer, and their relative integrations, could not be confidentally assigned, and the spectrum contains unknown by-products. The presence of the three isomers of **7** is inferred by the results of the I_2/THF quenching experiment detailed below. δ 0.77–0.82 (overlapping d, CH(C*H*$_3$)$_2$), 1.02–1.06 (overlapping d, CH(C*H*$_3$)$_2$), 1.30 (overlapping d, NCH(C*H*$_3$)$_2$), 1.83, 1.94, 1.96, 2.00 (Ar-C*H*$_3$), 3.53–3.62 (m, C*H*(CH$_3$)$_2$), 3.95–4.06 (m, NC*H*(CH$_3$)$_2$), 4.68, 4.78, 5.00 (s, Mg(μ-*H*)), 6.56–7.54 (m, Ar-*H*); ^{13}C NMR (101 MHz, C_6D_6, 298 K) δ 20.9, 21.3, 22.6 (Ar-*C*H$_3$), 22.7, 22.8, 23.1, 23.5 (NCH(*C*H$_3$)$_2$), 23.8, 23.9, 24.0, 24.1, 24.2, 25.6 (CH(*C*H$_3$)$_2$), 25.7 (Ar-*C*H$_3$), 25.8, 26.3 (CH(*C*H$_3$)$_2$), 26.4, 27.1, 27.4, 27.92, 27.95, 28.0 (*C*H(CH$_3$)$_2$), 49.7, 50.0, 50.2 (N*C*H(CH$_3$)$_2$), 123.3, 123.44, 123.48, 123.5, 123.64, 123.69, 123.7, 126.5, 127.4, 130.6, 134.5, 138.4, 141.7, 142.4, 142.65, 142.69, 142.90, 142.96, 143.1, 143.3, 144.1, 144.3, 144.6, 145.9, 149.4, 154.6 (Ar-*C*), 166.4, 167.4, 168.3 (*C*N$_3$); IR *ν*/cm^{-1} (Nujol): 1607 (s), 1580 (s), 1245 (s), 1182 (s), 1154 (s), 1109 (s), 1045 (s), 1002 (s), 932 (s), 871 (s), 829 (s), 799 (s), 756 (s), 715 (s); a satisfactory micro analysis could not obtained as the product could not be crystallized, and contains PrisoH and small amounts of other by-products.

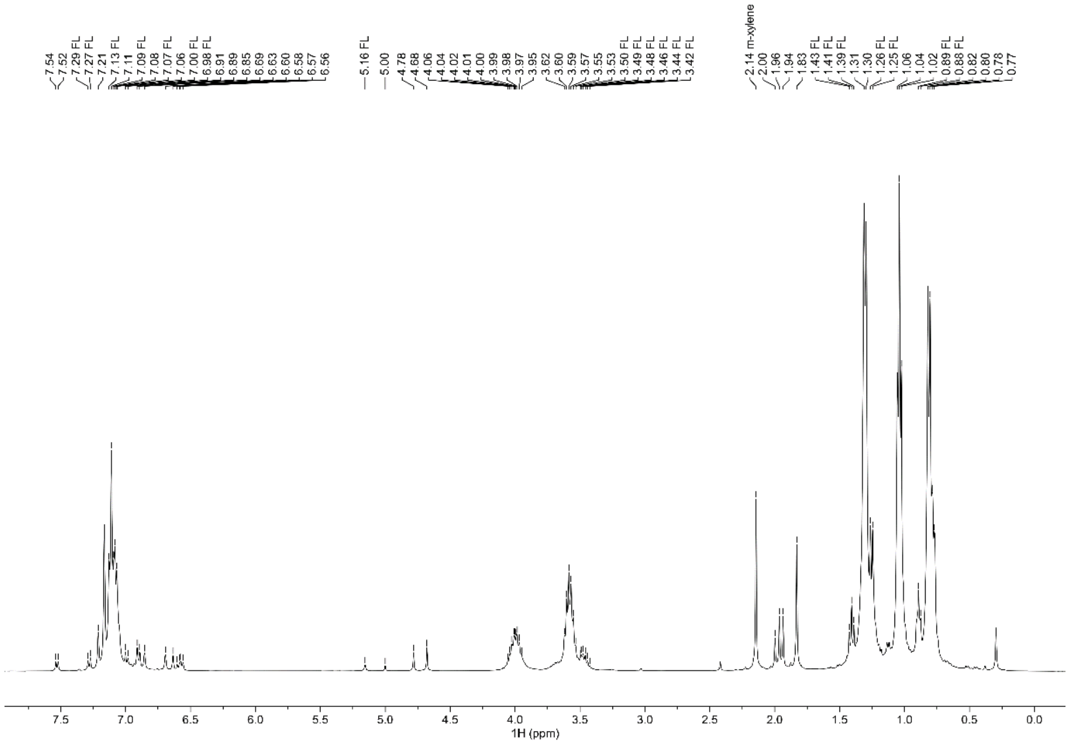

Figure S13. ^{1}H NMR spectrum (400 MHz, 298 K, C_6D_6) of an isomeric mixture of **7**, and reaction by-products (FL = PrisoH).

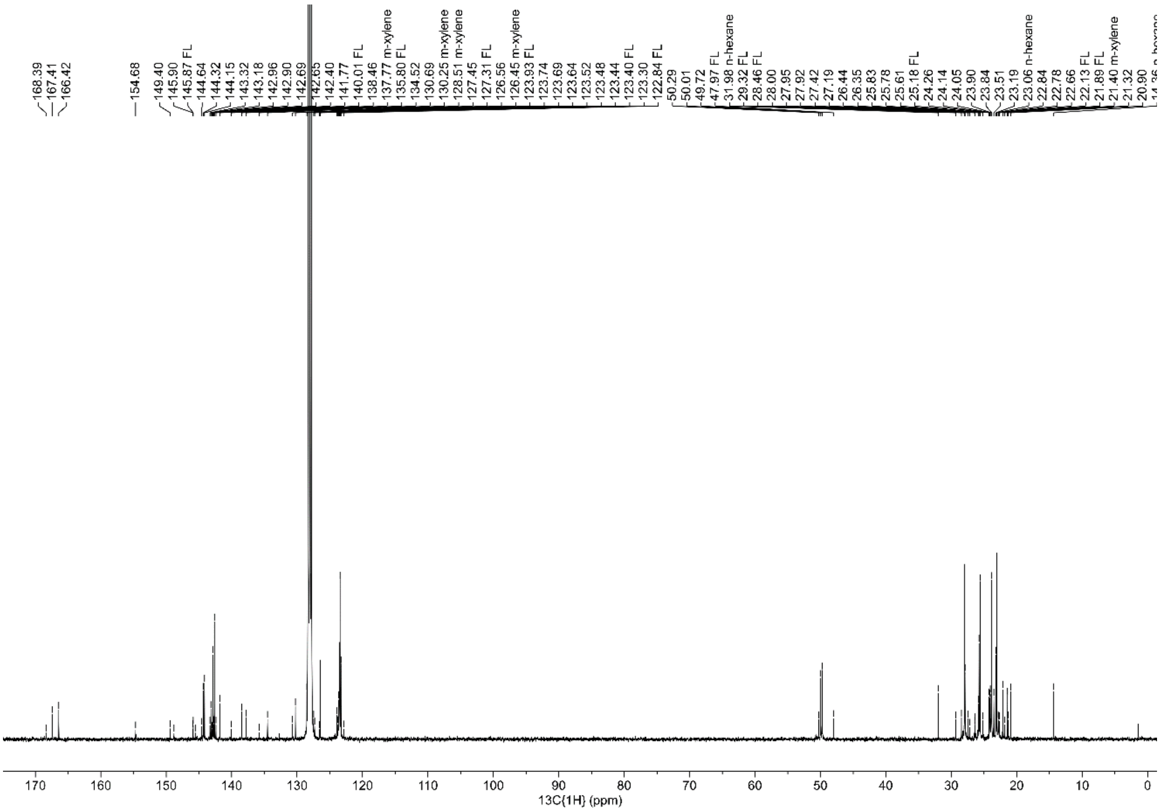

Figure S14. $^{13}C\{^{1}H\}$ NMR spectrum (101 MHz, 298 K, C_6D_6) of an isomeric mixture of **7**, and reaction by-products (FL = PrisoH).

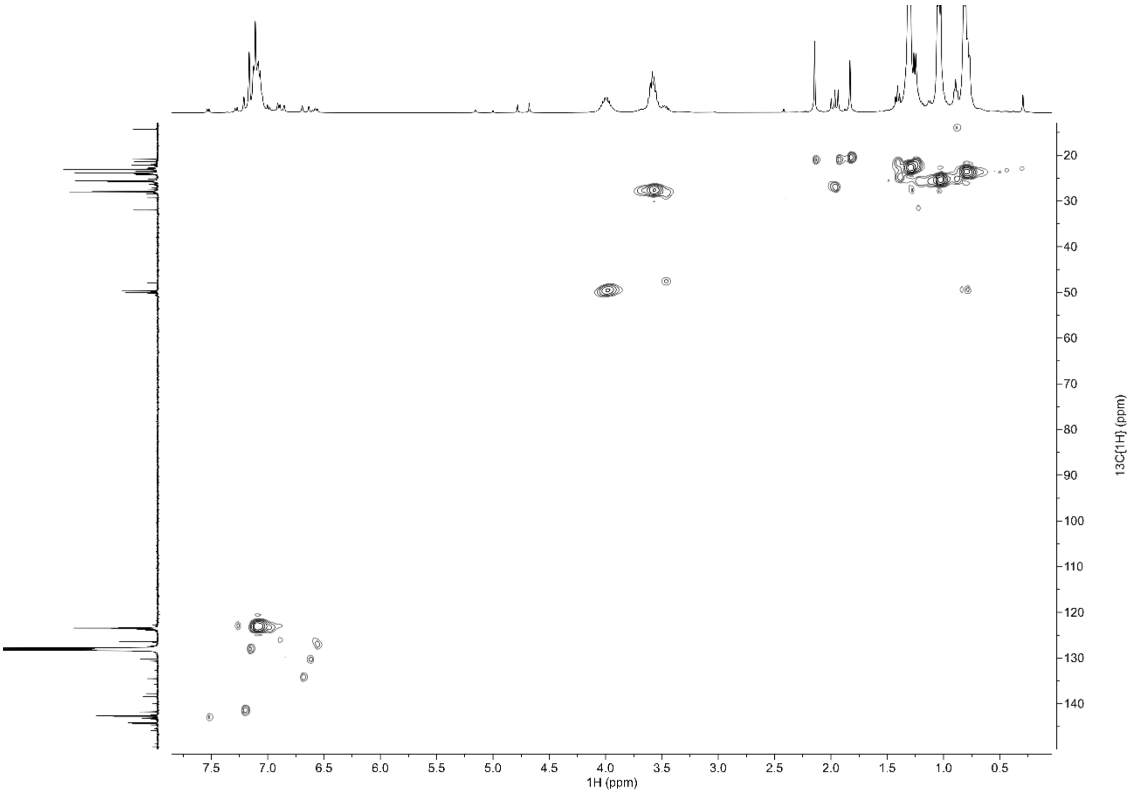

Figure S15. HMQC spectrum (^{1}H: 400 MHz; ^{13}C: 101 MHz, 298 K, C_6D_6) of an isomeric mixture of **7**, and reaction by-products.

[{(Priso)Mg}$_2$(μ-H)(μ-*p*-$C_6H_3Me_2$)] (8).

Compound **3** (100 mg, 0.103 mmol) was suspended in 4 mL of *p*-xylene in a 10 mm J. Young's NMR tube. The sample was irradiated with UV light for 8h. Volatiles were then removed *in vacuo*, the residue redissolved in *ca.* 4 mL of hexane, filtered and the filtrate dried under vacuum, yielding **8** as a pale-yellow powder (96 mg, 87 %). All attempts to recrystallize **8** failed. The presence of **8** is inferred by the results of the I_2/THF quenching experiment detailed below. M.p: 148–152 °C; ^{1}H NMR (400 MHz, C_6D_6, 298 K) δ 0.81 (d, *J* = 5.3 Hz, 24H, CH(C*H*$_3$)$_2$), 0.96 (d, *J* = 6.7 Hz, 12H, CH(C*H*$_3$)$_2$), 1.12 (d, *J* = 6.7 Hz, 12H, CH(C*H*$_3$)$_2$), 1.28–1.32 (overlapping d, *J* = 6.8 Hz, 24H, NCH(C*H*$_3$)$_2$), 1.69 (s, 3H, Ar-C*H*$_3$), 1.92 (s, 3H, Ar-C*H*$_3$), 3.52–3.63 (m, 8H, C*H*(CH$_3$)$_2$), 3.99–4.06 (m, 4H, NC*H*(CH$_3$)$_2$), 4.71 (s, 1H, Mg(μ-*H*)), 6.75–7.33 (m, 15H, Ar-*H*); ^{13}C NMR (101 MHz, C_6D_6, 298 K) δ 21.0 (Ar-*C*H$_3$), 23.1, 23.3 (NCH(*C*H$_3$)$_2$), 24.0, 24.1, 25.4, 25.8 (CH(*C*H$_3$)$_2$), 27.1 (Ar-C*H*$_3$), 27.95, 27.98 (*C*H(CH$_3$)$_2$), 49.9 (N*C*H(CH$_3$)$_2$), 123.42, 123.49, 123.6, 129.2, 133.5, 135.6, 142.73, 142.79, 143.7, 144.3, 150.0, 151.6 (Ar-*C*), 166.9 (*C*N$_3$); IR *v*/cm^{-1} (Nujol): 1607 (s), 1575 (s), 1251 (s), 1182 (m), 1146 (m), 1107 (m), 1045 (m), 1002 (m), 862 (w), 830 (w), 798 (s), 760 (s); a satisfactory micro analysis could not obtained as the product could not be crystallized, and contains a small amount of PrisoH, which could not be separated.

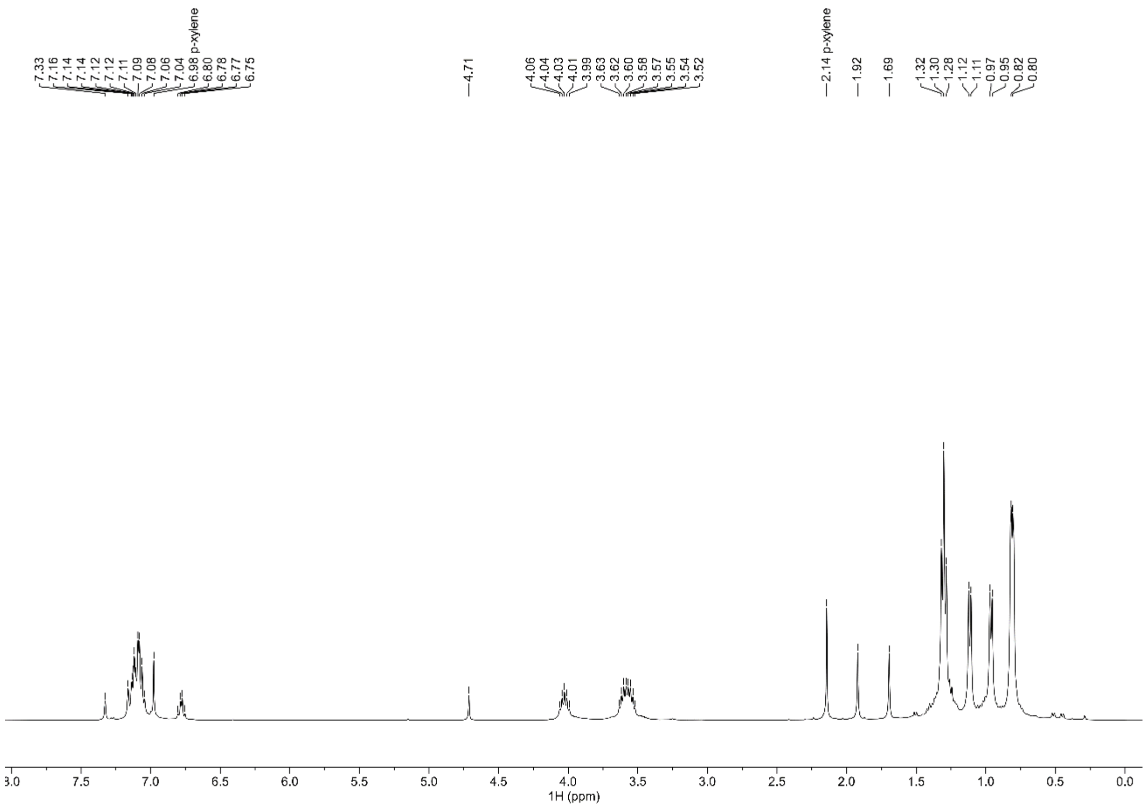

Figure S16. ^{1}H NMR spectrum (400 MHz, 298 K, C_6D_6) of **8**.

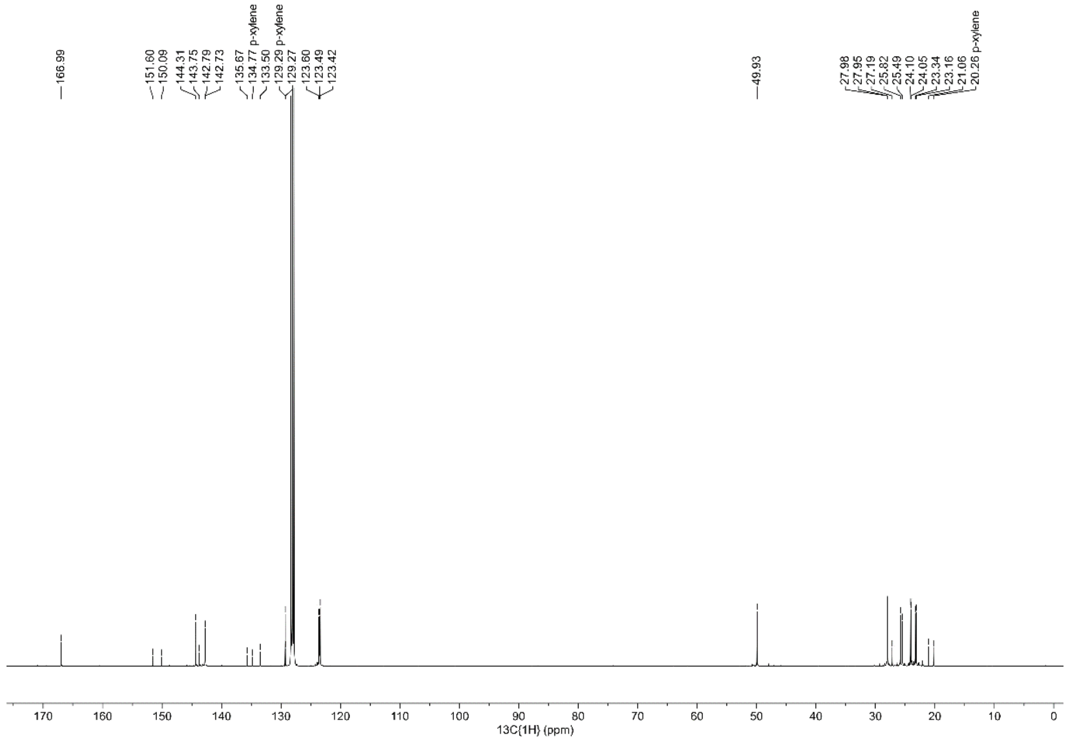

Figure S17. $^{13}C\{^1H\}$ NMR spectrum (101 MHz, 298 K, C_6D_6) of **8**.

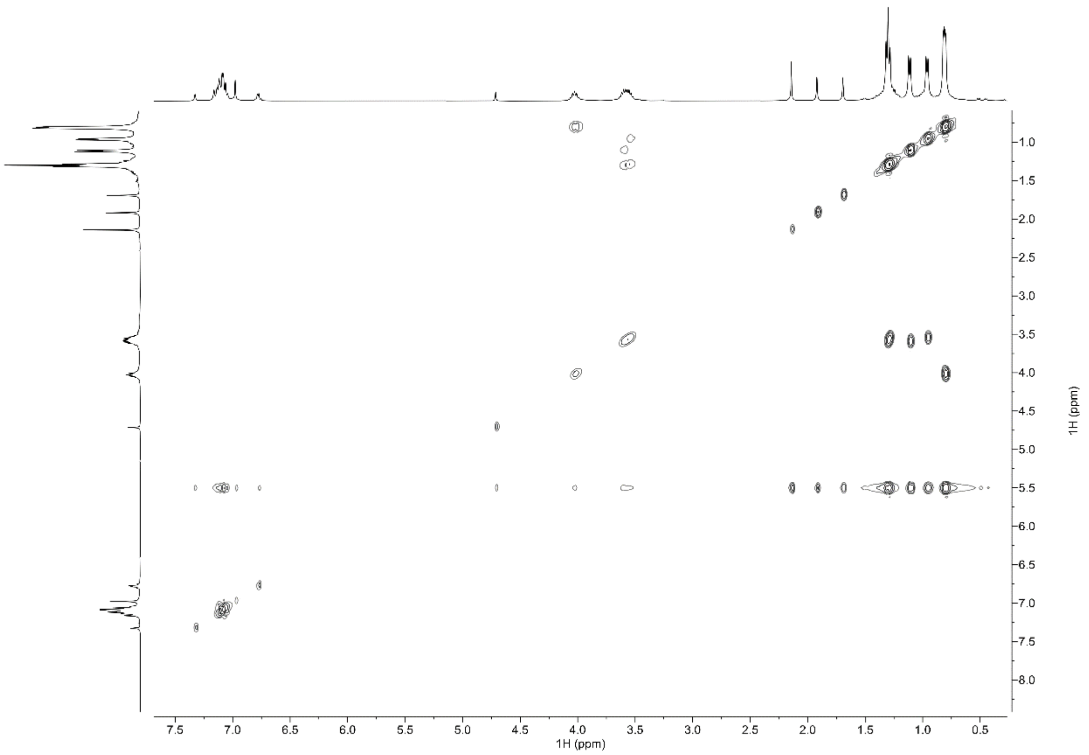

Figure S18. ^1H-COSY NMR spectrum (400 MHz, 298 K, C_6D_6) of **8**.

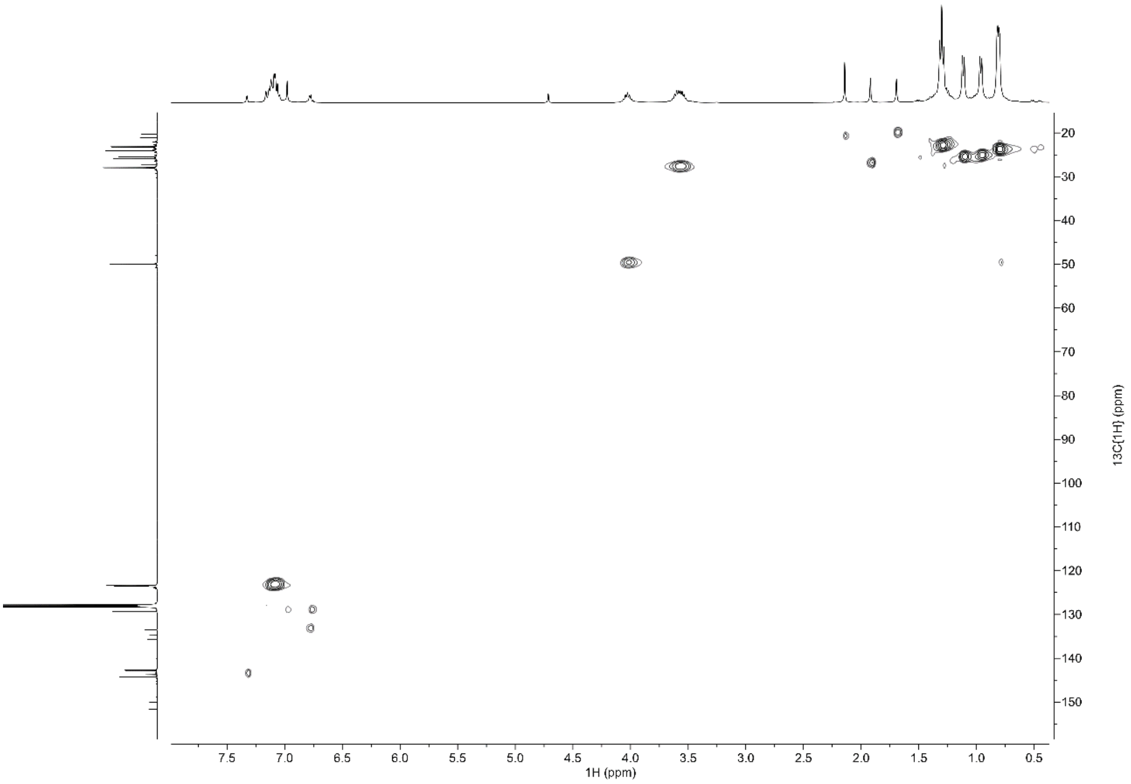

Figure S19. HMQC spectrum (^{1}H: 400 MHz; ^{13}C: 101 MHz, 298 K, C_6D_6) of **8**.

[{(Priso)Mg}$_2$(μ-H)(μ-$C_6H_2Me_3$)] (9).

Compound **3** (80 mg, 0.082 mmol) was suspended in 4 mL of mesitylene in a 10 mm J. Young's NMR tube. The sample was irradiated with UV light for 10h, volatiles were then removed *in vacuo*, and the oily residue dissolved in *ca.* 4 mL of hexane, filtered and the filtrate dried under vacuum, yielding **9** as a pale-yellow oil (63 mg, 70 %). ^{1}H NMR (400 MHz, C_6D_6, 298 K) δ 0.79 (d, *J* = 7.0 Hz, 24H, CH(C*H*$_3$)$_2$), 1.06 (d, *J* = 6.7 Hz, 24H, CH(C*H*$_3$)$_2$), 1.32 (d, *J* = 6.8 Hz, 24H, NCH(C*H*$_3$)$_2$), 1.99 (s, 6H, Ar-C*H*$_3$), 2.00 (s, 3H, Ar-C*H*$_3$), 3.53–3.60 (m, 8H, C*H*(CH$_3$)$_2$), 4.02–4.09 (m, 4H, NC*H*(CH$_3$)$_2$), 5.01 (s, 1H, Mg(μ-*H*)), 6.45 (s, 2H, mesityl Ar-*H*), 7.05–7.12 (m, 12H, Ar-*H*); ^{13}C{^{1}H} NMR (101 MHz, C_6D_6, 298 K) δ 21.2 (Ar-*C*H$_3$), 23.5 (NCH(*C*H$_3$)$_2$), 24.2, 25.8 (CH(*C*H$_3$)$_2$), 27.0 (Ar-*C*H$_3$), 27.9 (*C*H(CH$_3$)$_2$), 50.2 (N*C*H(CH$_3$)$_2$), 123.3, 123.61, 123.68, 127.7, 142.4, 143.1, 144.7, 154.6 (Ar-*C*), 168.3 (*C*N$_3$); IR *ν*/cm^{-1} (Nujol): 1609 (s), 1584 (s), 1254 (s), 1182 (s), 1153 (s), 1125 (s), 1038 (s), 999 (s), 934 (s), 872 (s), 836 (s), 801 (s), 758 (s), 715 (s); a satisfactory micro analysis could not obtained as the product is an oil and could not be crystallized. Furthermore, it contains residual mesitylene, and a small amount of PrisoH, which could not be separated.

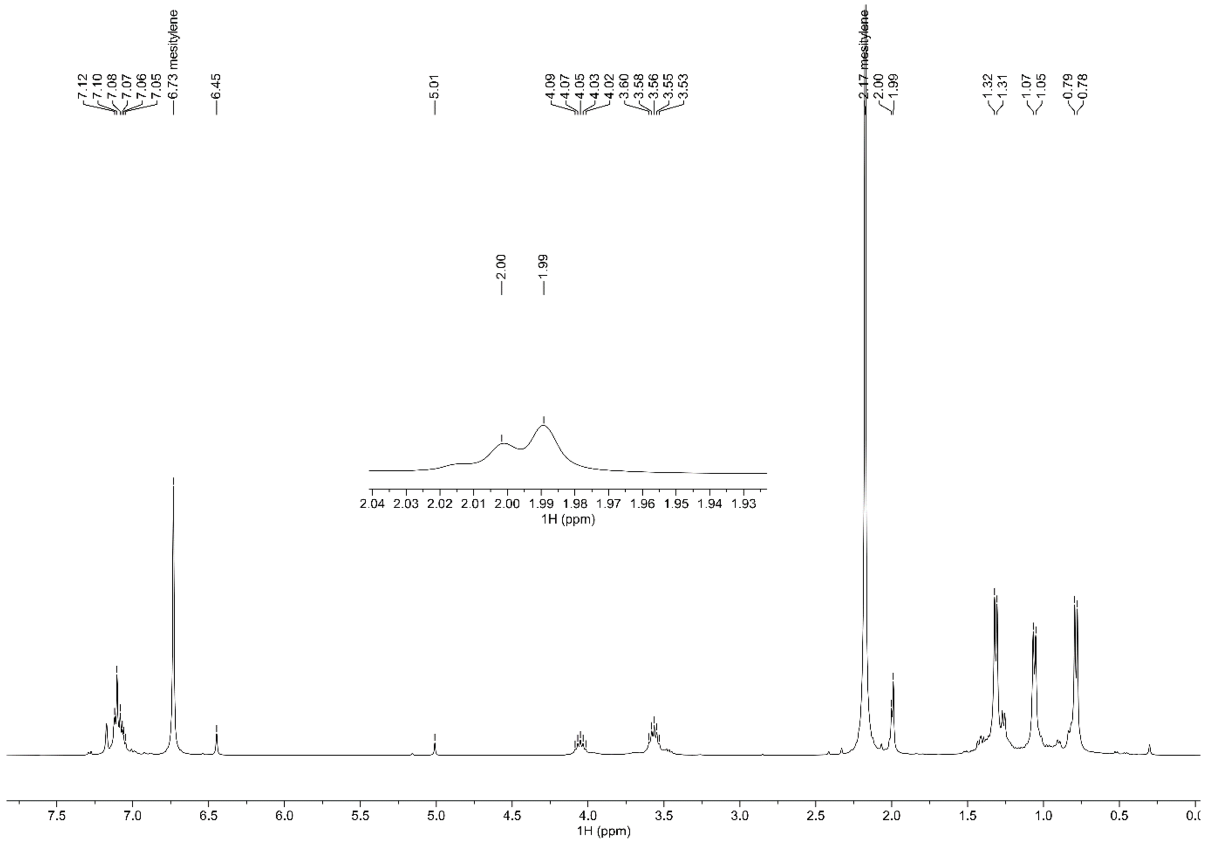

Figure S20. ^{1}H NMR spectrum (400 MHz, 298 K, C_6D_6) of **9** (methyl resonances inset).

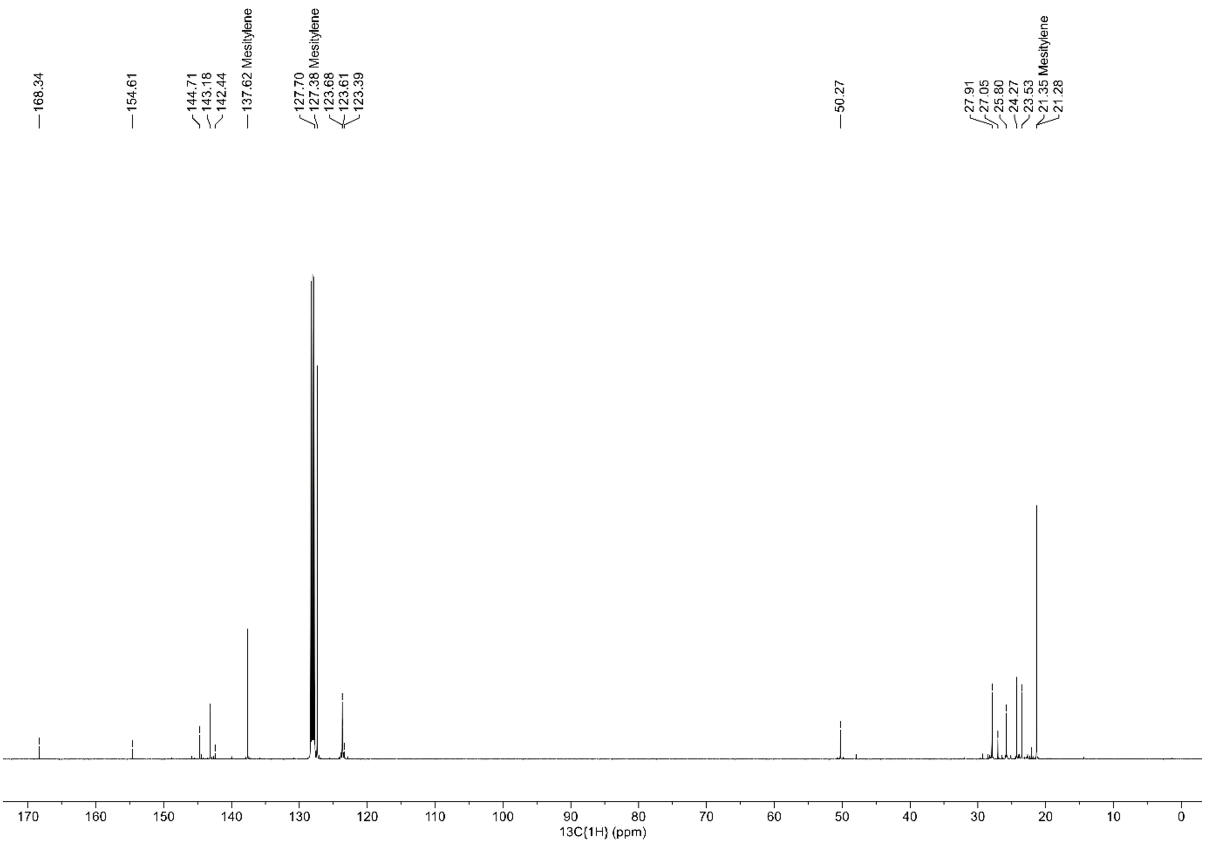

Figure S21. ^{13}C{^{1}H} NMR spectrum (101 MHz, 298 K, C_6D_6) of **9**.

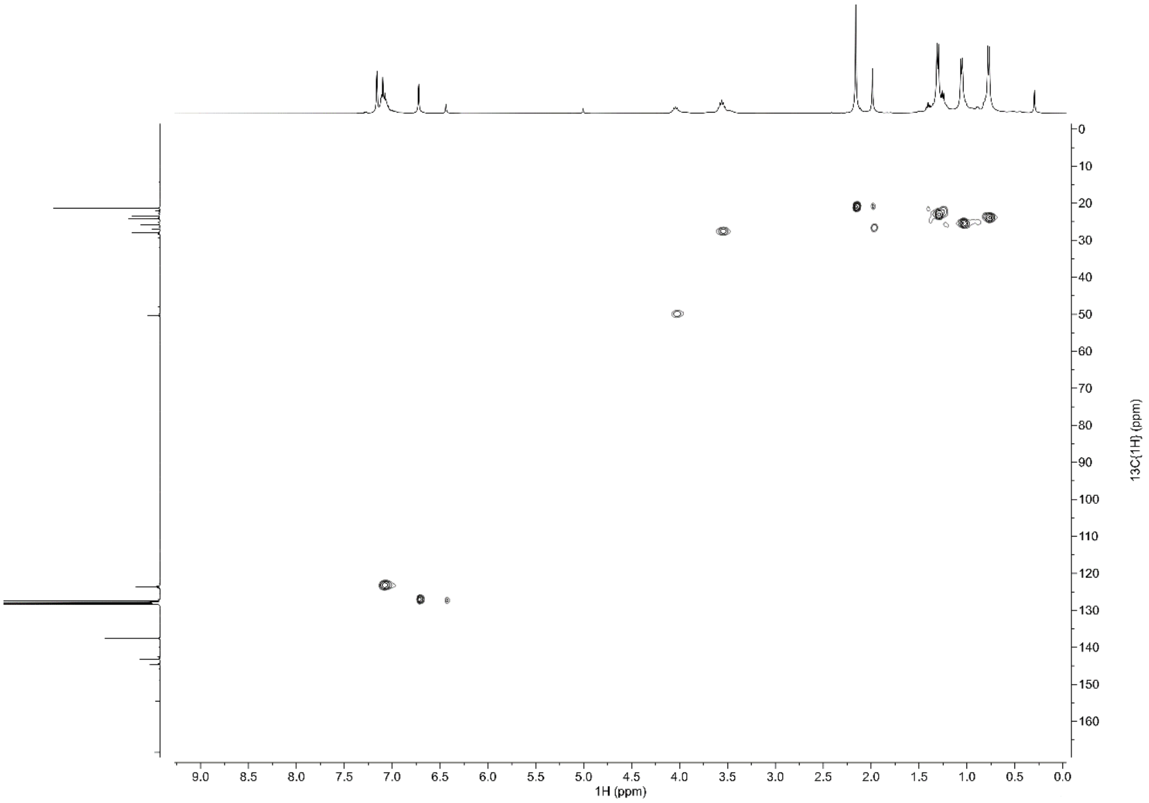

Figure S22. HMQC spectrum (^{1}H: 400 MHz; ^{13}C: 101 MHz, 298 K, C_6D_6) of **9**.

[(Priso)Mg(py$^{Bu\textit{t}}$H)(py$^{Bu\textit{t}}$)$_2$] (10).

Compound **3** (150 mg, 0.154 mmol) was dissolved in 4 mL of toluene and cooled to -78 °C, and 3.7 mL of a 0.25 M solution (0.924 mmol) of 4-*tert*-butylpyridine (py$^{Bu\textit{t}}$) in toluene was added to it. The solution was warmed to room temperature, during which time it turned dark red in color. After stirring overnight, all volatiles were removed *in vacuo*, the residue dissolved in *ca.* 4 mL of hexane, and then filtered. The filtrate was concentrated to *ca.* 2 mL, and placed at -30 °C for 3 d to give an orange crystalline solid (45 mg). Visual inspection of the solid revealed it to contain at least two crystalline solids. A number of single crystals were selected for X-ray crystallographic studies. Only one crystal type was able to be identified by X-ray crystallography as being compound **10**. As the isolated solid is a mixture of products, NMR spectra of compound **10** could not be confidentally assigned.

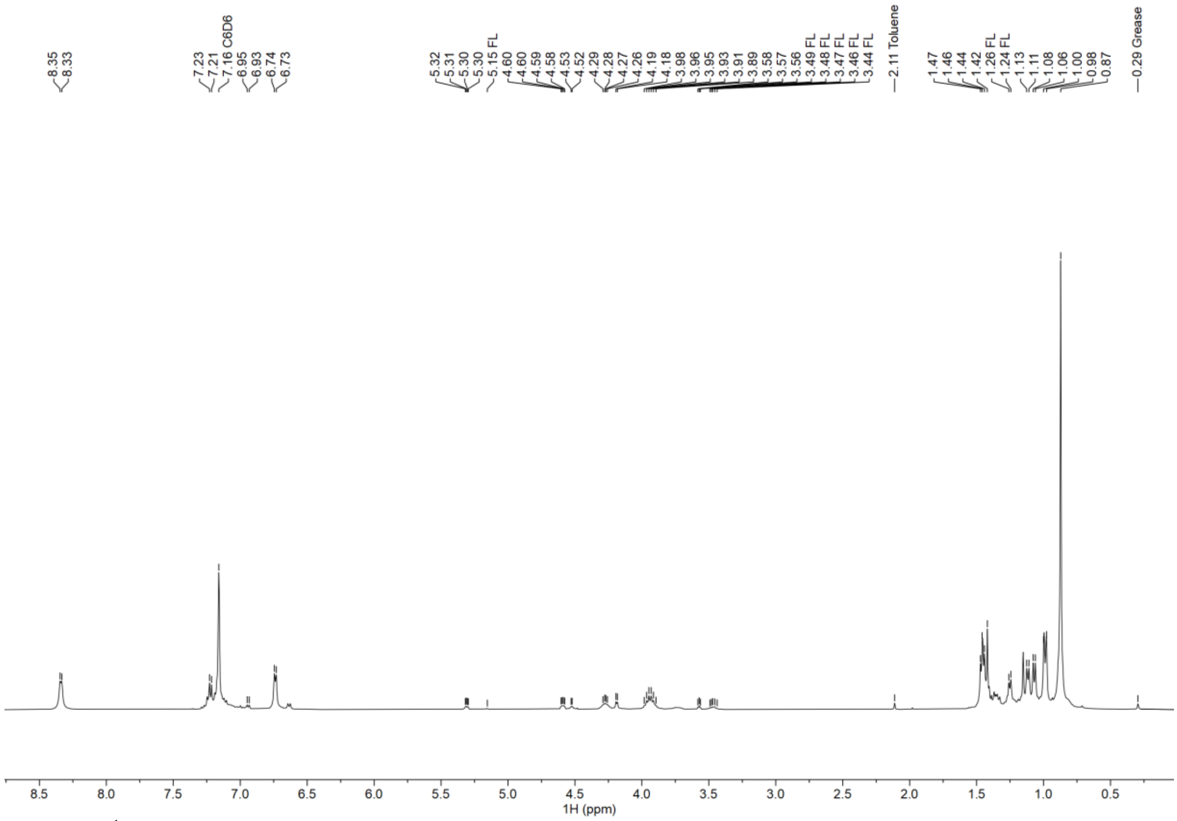

Figure S23. ^{1}H NMR spectrum (400 MHz, 298 K, C_6D_6) of the red crystalline material which contains compound **10** as one component (FL = PrisoH).

[{Mg(μ-Priso^{-H})}$_2$] (12).

Compound **3** (100 mg, 0.103 mmol) was suspended in 5 mL of cyclohexane in a 10 mm J. Young's NMR tube. The sample was irradiated with UV light for 8h, during which time the colorless suspension turned to a colorless solution. All the volatiles were removed *in vacuo* and the residue dissolved in *ca.* 4 mL of hexane, filtered, and the filtrate concentrated to *ca.* 2 mL, and placed at -30 °C for 2 d. After this time, colourless crystals of **12** had deposited (63 mg, 63 %). The NMR spectra for the compound suggest the compound has a more symmetrical structure in solution than in the solid state. Variable temperature NMR experiments did not shed light on the apparent fluxional process the compound is undergoing in solution. ^{1}H NMR (400 MHz, C_6D_6, 298 K) δ 0.81 (d, J = 7.0 Hz, CH(CH_3)$_2$), 1.03 (d, J = 7.1 Hz, CH(CH_3)$_2$), 1.31 (d, J = 6.7 Hz, NCH(CH_3)$_2$), 3.57–3.60 (m, CH(CH$_3$)$_2$), 3.93–3.97 (m, NCH(CH$_3$)$_2$), 7.06–7.11 (m, Ar-H); $^{13}C\{^{1}H\}$ NMR (101 MHz, C_6D_6, 298 K) δ 22.6, 23.8 (CH(CH$_3$)$_2$), 26.4 (NCH(CH$_3$)$_2$), 27.9 (CH(CH$_3$)$_2$), 49.9 (NCH(CH$_3$)$_2$), 123.5, 142.6, 144.1 (Ar-C), 166.8 (CN$_3$); IR ν/cm^{-1} (Nujol): 1611 (s), 1583 (s), 1424 (s), 1248 (s), 1184 (s), 1155 (s), 1111 (s), 1047 (s), 1002 (s), 934 (s), 872 (s), 830 (s), 800 (s), 756 (s), 716 (s), 658 (s); a satisfactory micro analysis could not obtained as the product contains a small amount of PrisoH, which could not be separated by recrystallization.

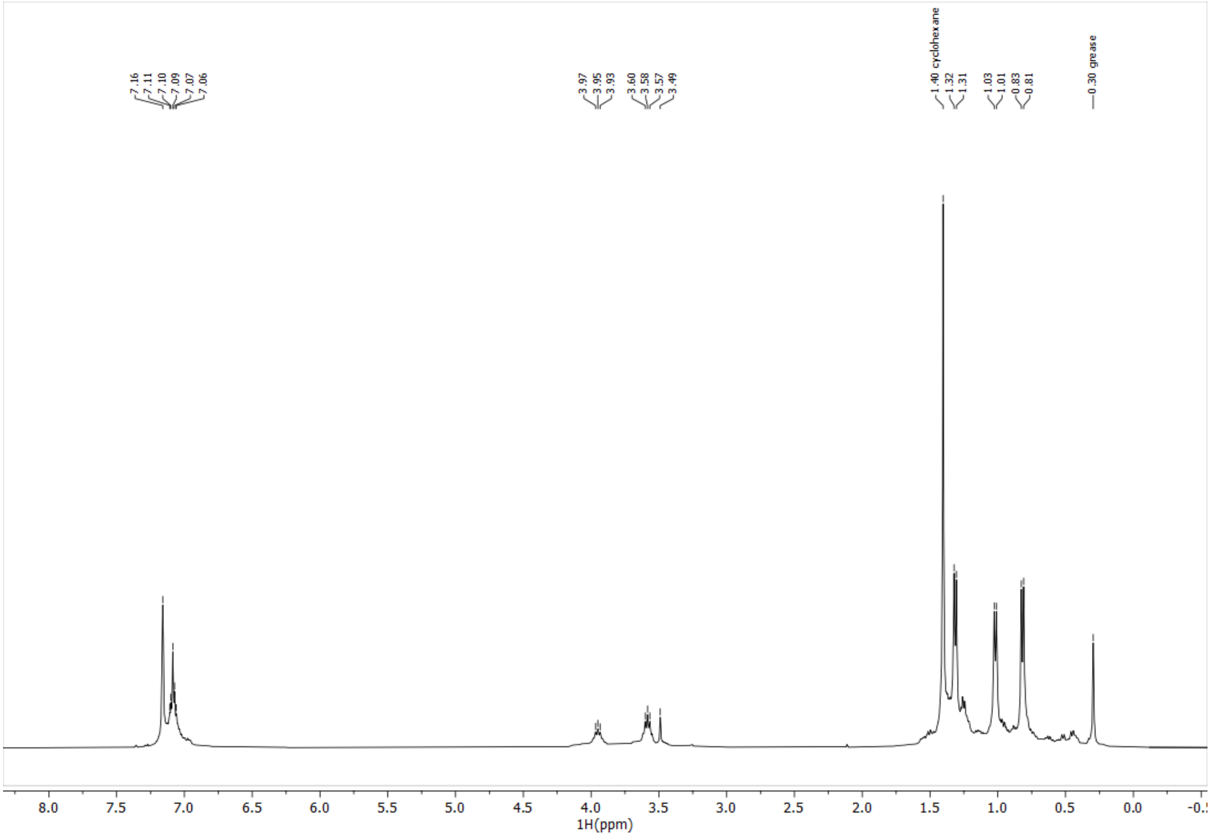

Figure S24. ^{1}H NMR specturm (400 MHz, 298 K, C_6D_6) of **12** containing residual cyclohexane.

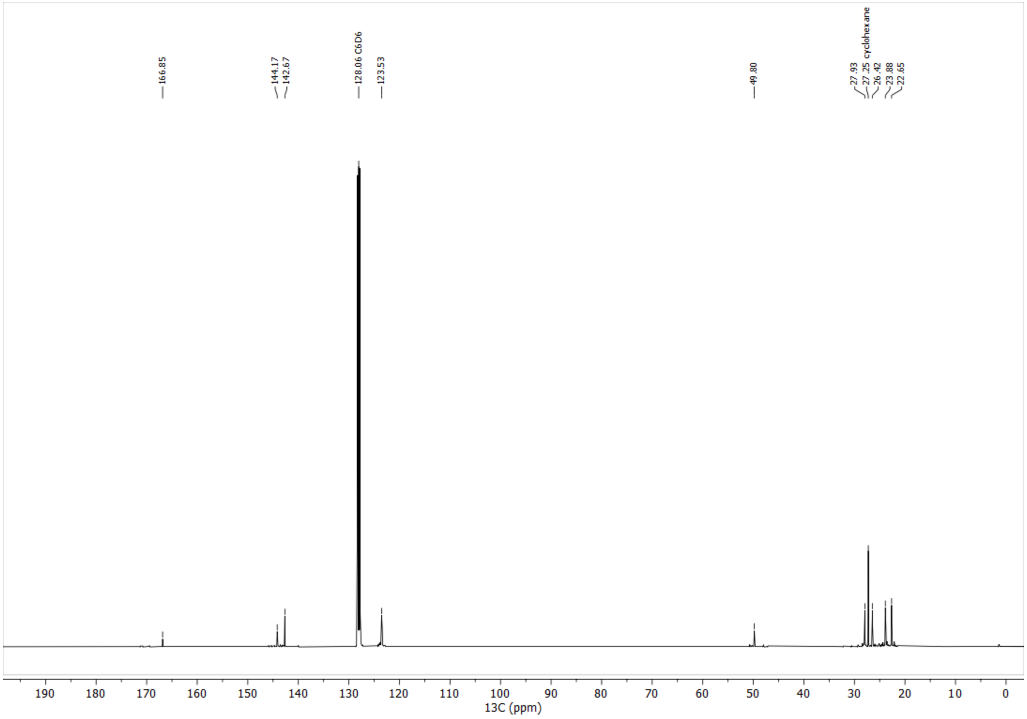

Figure S25. $^{13}C\{^{1}H\}$ NMR spectrum (101 MHz, 298 K, C_6D_6) of **12** containing residual cyclohexane.

[(Priso)Mg–Mg(^{Dip}Nacnac)] (13).

NMR scale: Compound **3** (10 mg, 0.01 mmol) and [{(^{Dip}Nacnac)Mg}$_2$] (9 mg, 0.01 mmol) were suspended in 0.5 mL of C_6D_6 in a J. Young's NMR tube at room temperature. The sample was irradiated with blue light, and the progress of the reaction monitored by ^{1}H NMR spectroscopy. After 4h, near quantitative conversion to compound **13** had occurred.

Preparatory scale: Compound **3** (50 mg, 0.051 mmol) and [{(^{Dip}Nacnac)Mg}$_2$] (45 mg, 0.051 mmol) were suspended in 4 mL of toluene in a 10 mm J. Young's NMR tube at room temperature. The sample was irradiated with blue light for 4 h. Volatiles were then removed *in vacuo*, the residue dissolved in *ca.* 4 mL of hexane, filtered, the filtrate concentrated to *ca.* 2 mL, and placed at -30 °C for 2 d. After this time, pale yellow crystals of **13** were isolated (81 mg, 85 %). M.p: 223-228 °C; ^{1}H NMR (400 MHz, C_6D_6, 298 K) δ 0.74 (d, *J* = 7.0 Hz, 12H, NCH(C*H*$_3$)$_2$), 1.07 (d, *J* = 6.8 Hz, 12H, CH(C*H*$_3$)$_2$), 1.10 (d, *J* = 6.9 Hz, 12H, CH(C*H*$_3$)$_2$), 1.13 (d, *J* = 7.1 Hz, 12H, CH(C*H*$_3$)$_2$), 1.33 (d, *J* = 6.9 Hz, 12H, CH(C*H*$_3$)$_2$), 1.62 (s, 6H, NCC*H*$_3$), 3.10–3.17 (m, 4H, C*H*(CH$_3$)$_2$), 3.45–3.52 (m, 4H, C*H*(CH$_3$)$_2$), 3.93– 4.02 (m, 2H, NC*H*(CH$_3$)$_2$), 4.90 (s, 1H, β-C*H*), 7.05–7.11 (m, 12H, Ar-*H*); ^{13}C{^{1}H} NMR (101 MHz, C_6D_6, 298 K) δ 22.5 (NCH(*C*H$_3$)$_2$), 23.9 (NC*C*H$_3$), 24.2, 24.4, 25.5, 27.2 (CH(*C*H$_3$)$_2$), 28.2, 28.7 (*C*H(CH$_3$)$_2$), 49.7 (N*C*H(CH$_3$)$_2$), 95.9 (β-*C*H), 123.1, 123.3, 123.9, 125.8, 142.3, 142.9, 144.80, 144.87 (Ar-*C*), 168.21 (*C*N$_3$), 168.24 (N*C*CH$_3$); IR *ν*/cm^{-1} (Nujol): 1610 (s), 1521 (s), 1233 (s), 1173 (s), 1103 (s), 1020 (s), 929 (s), 871 (s), 793 (s), 750 (s), 700 (s); anal. calc. for $C_{60}H_{89}Mg_2N_5$: C 77.57 %, H 9.66 %, N 7.54 %: found: C 77.14 %, H 9.79 %, N 7.38 %.

N.B. When the irradiation of **3** and [{(^{Dip}Nacnac)Mg}$_2$] was carried out under 2:1 stoichiometery, all of [{(^{Dip}Nacnac)Mg}$_2$] was consumed, giving compound 13, and unreacted **3**. When the reaction was carried out under a 1:2 stoichiometery, compound **13** was formed, in addition to the previously reported "Birch" product **1** (n = 0).

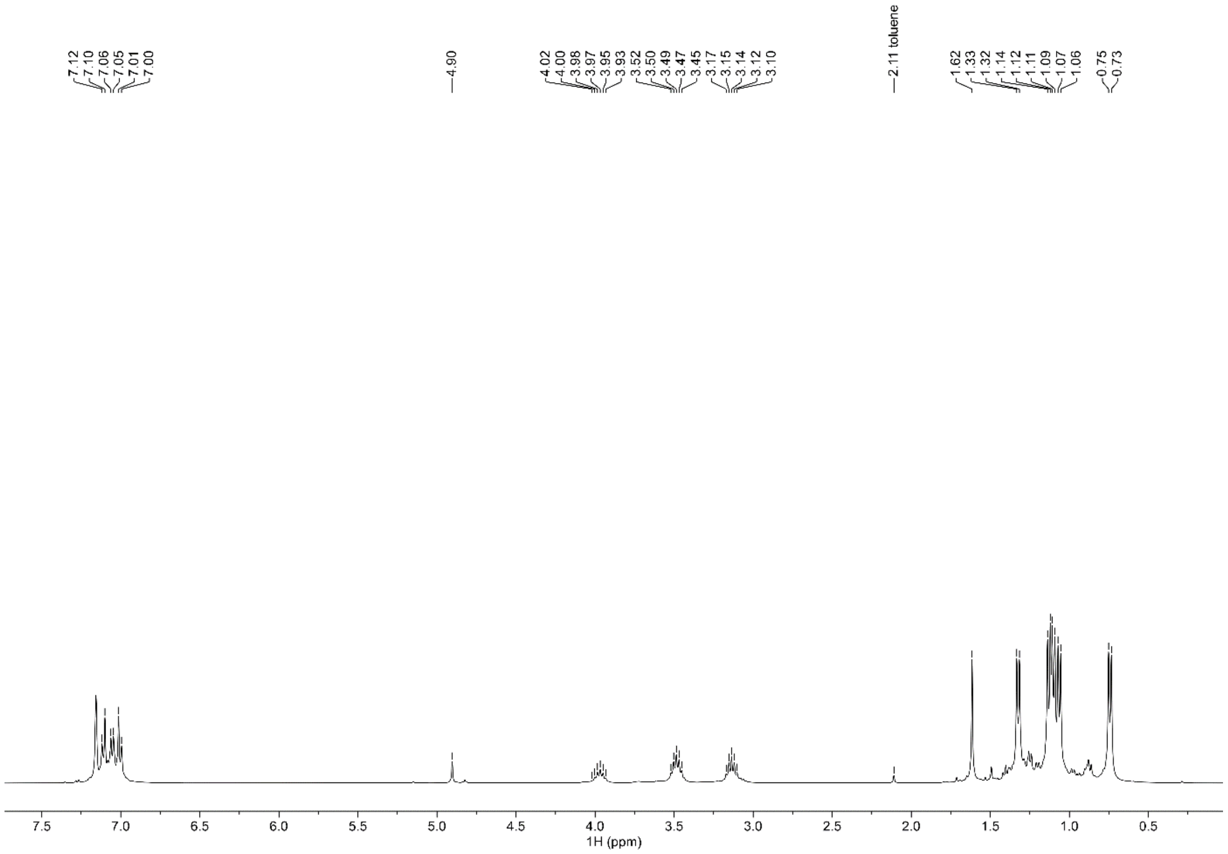

Figure S26. ^{1}H NMR spectrum (400 MHz, 298 K, C_6D_6) of **13**.

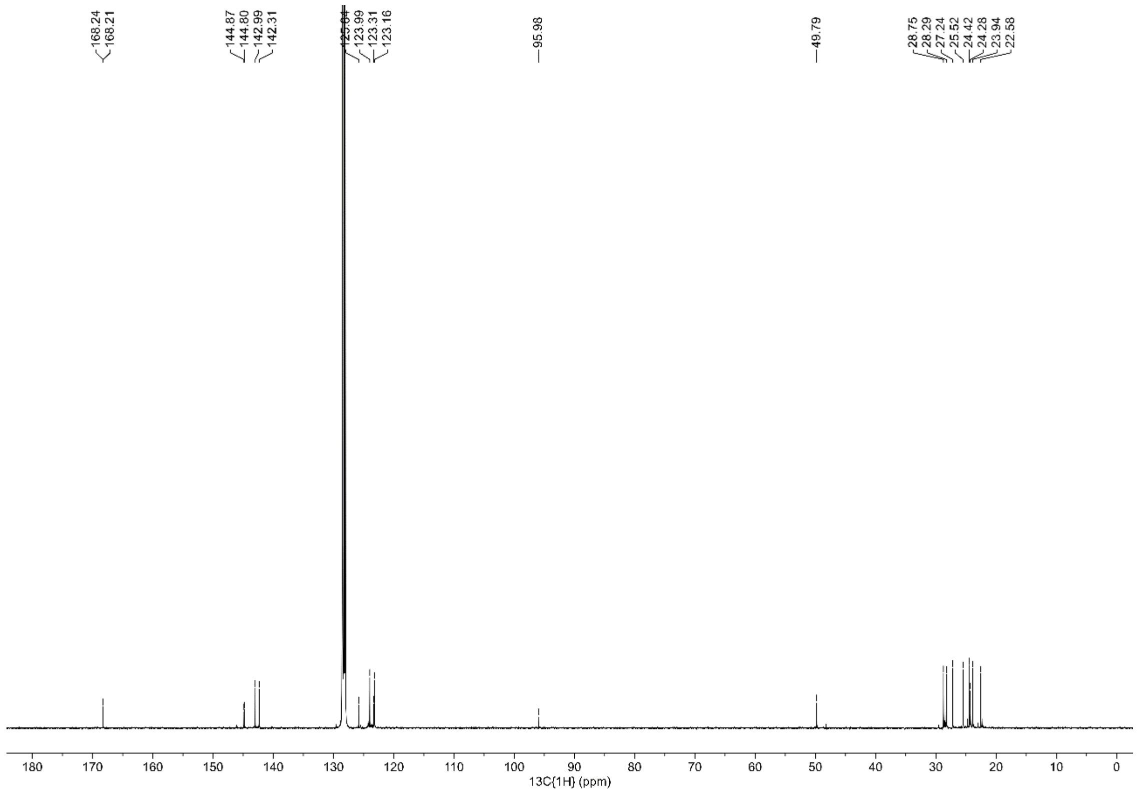

Figure S27. $^{13}C\{^{1}H\}$ NMR spectrum (101 MHz, 298 K, C_6D_6) of **13**.

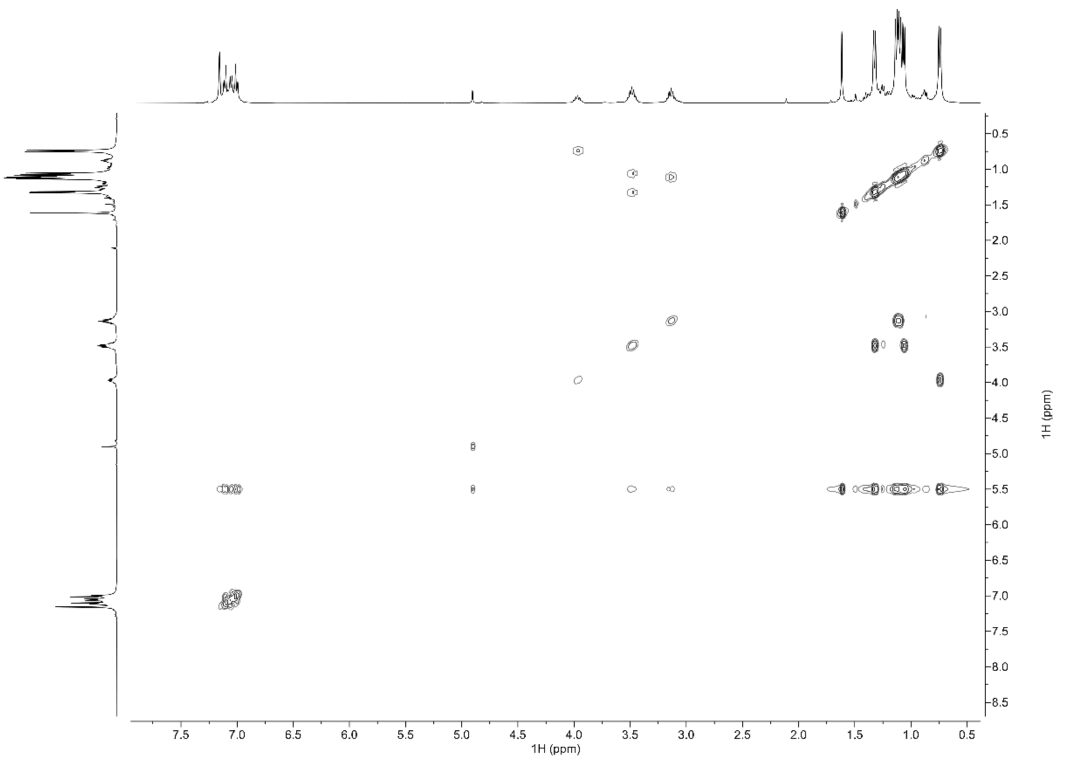

Figure S28. ^{1}H-COSY NMR spectrum (400 MHz, 298 K, C_6D_6) of **13**.

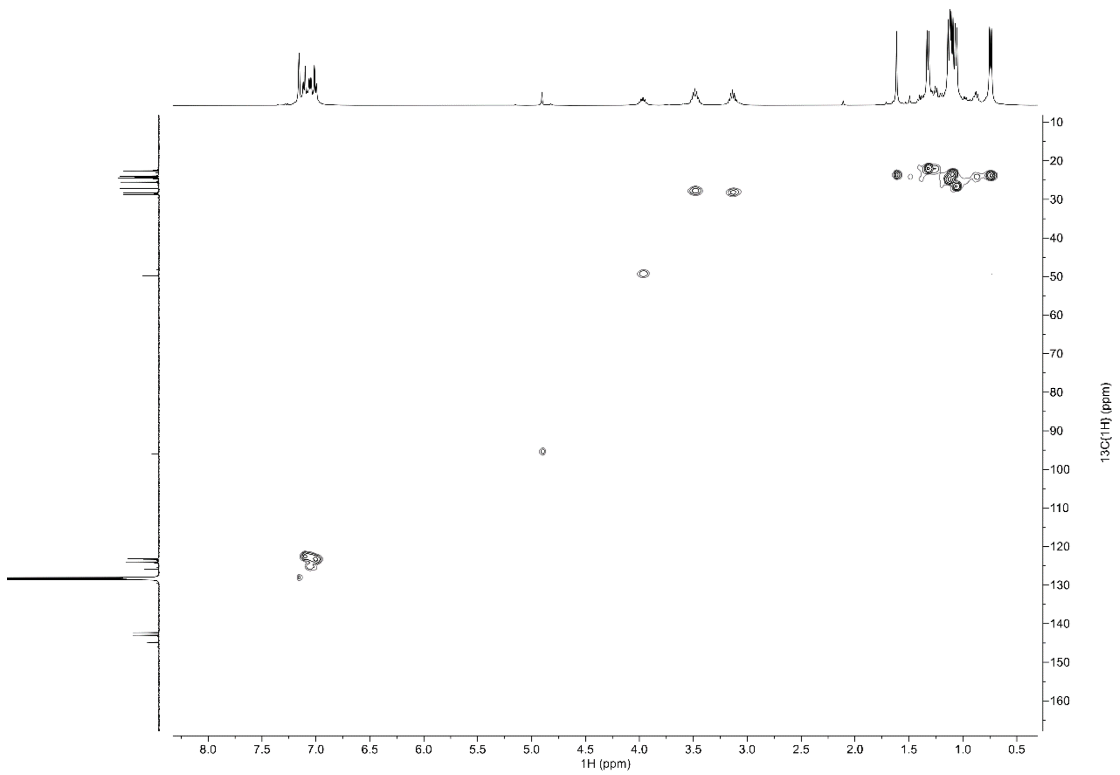

Figure S29. HMQC spectrum (^{1}H: 400 MHz; ^{13}C: 101 MHz, 298 K, C_6D_6) of **13**.

[Mg(Priso)$_2$].

PrisoH (0.63 g, 1.36 mmol) was dissolved in toluene and the solution cooled to -78 °C. A 1.0 M solution of $Bu^n{}_2Mg$ (0.65 mL, 0.65 mmol) was then added to the reaction mixture, and the solution warmed to room temperature. The solution was then heated at 75 °C for 2 h, after which time volatiles were removed *in vacuo*. The product was dissolved in *n*-pentane, filtered, and the filtrate concentrated to 1 mL and kept at -30 °C for one week. No crystals were obtained. Accordingly, volatiles were removed, leaving a colorless solid, the predominant component of which was [Mg(Priso)$_2$] (0.58 g, 90 %). M.p: 248-251 °C; ^{1}H NMR (400 MHz, C_6D_6, 298 K) δ 0.45 (d, *J* = 6.9 Hz, 12H, NCH(C*H*$_3$)$_2$), 0.52 (d, *J* = 6.7 Hz, 12H, NCH(C*H*$_3$)$_2$), 0.97 (d, *J* = 7.0 Hz, 12H CH(C*H*$_3$)$_2$), 1.25 (d, *J* = 6.6 Hz, 12H, CH(C*H*$_3$)$_2$), 1.33 (d, *J* = 6.9 Hz, 12H, CH(C*H*$_3$)$_2$), 1.51 (d, *J* = 6.8 Hz, 12H, CH(C*H*$_3$)$_2$), 3.20–3.30 (m, 4H, C*H*(CH$_3$)$_2$), 3.86–3.93 (m, 4H, C*H*(CH$_3$)$_2$), 3.93–4.00 (m, 4H, NC*H*(CH$_3$)$_2$), 6.96–7.15 (m, 12H, Ar-*H*); ^{13}C{^{1}H} NMR (101 MHz, C_6D_6, 298 K) δ 23.5, 23.6, 24.5, 25.1 (CH(*C*H$_3$)$_2$), 25.9 (NCH(*C*H$_3$)$_2$), 28.12, 28.18 (*C*H(CH$_3$)$_2$), 50.7 (N*C*H(CH$_3$)$_2$), 123.7, 123.9, 124.1, 143.2, 143.5, 145.3 (Ar-*C*), 170.9 (*C*N$_3$); IR ν/cm^{-1} (Nujol): 1605 (s), 1578 (s), 1420 (s), 1244 (w), 1218 (s), 1181 (m), 1152 (m), 1127 (w), 1108 (m), 1044 (w), 1000 (m), 953 (w), 931 (m); a satisfactory micro analysis could not obtained as the product is very soluble in all common organic solvents, could not be crystallized, and contains a small amount of an unknown impurity.

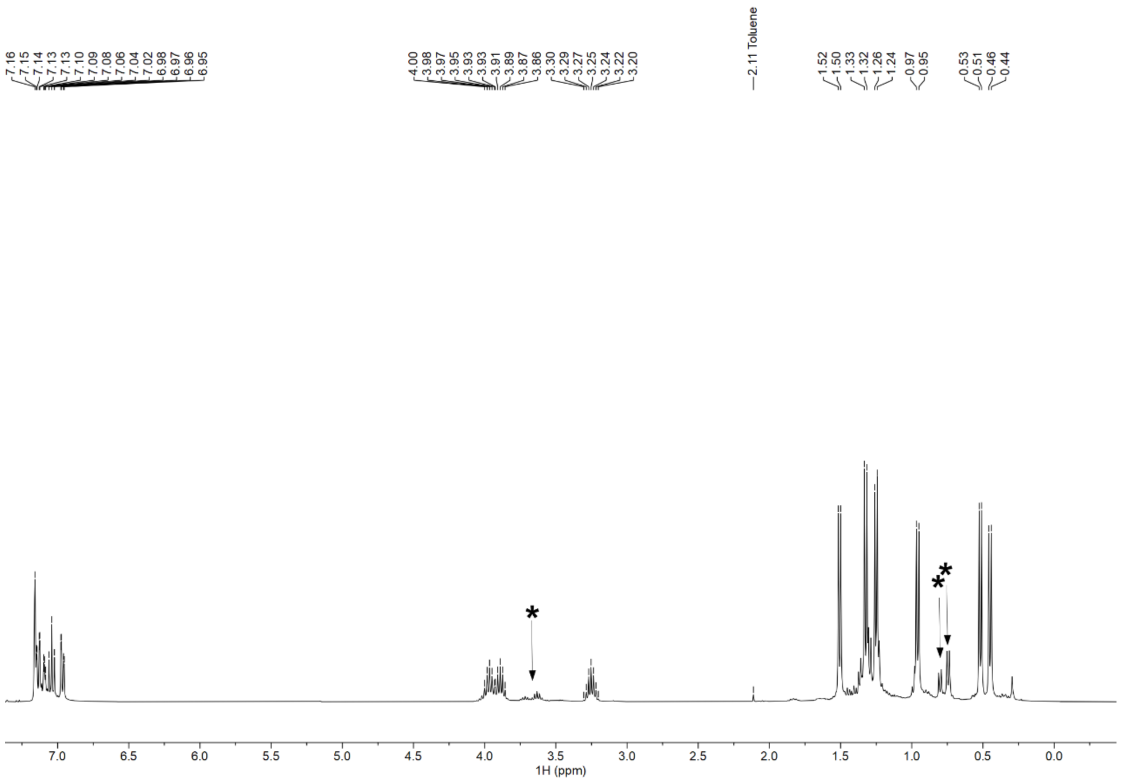

Figure S30. ^{1}H NMR spectrum (400 MHz, 298 K, C_6D_6) of [Mg(Priso)$_2$] (* denotes unidentified species).

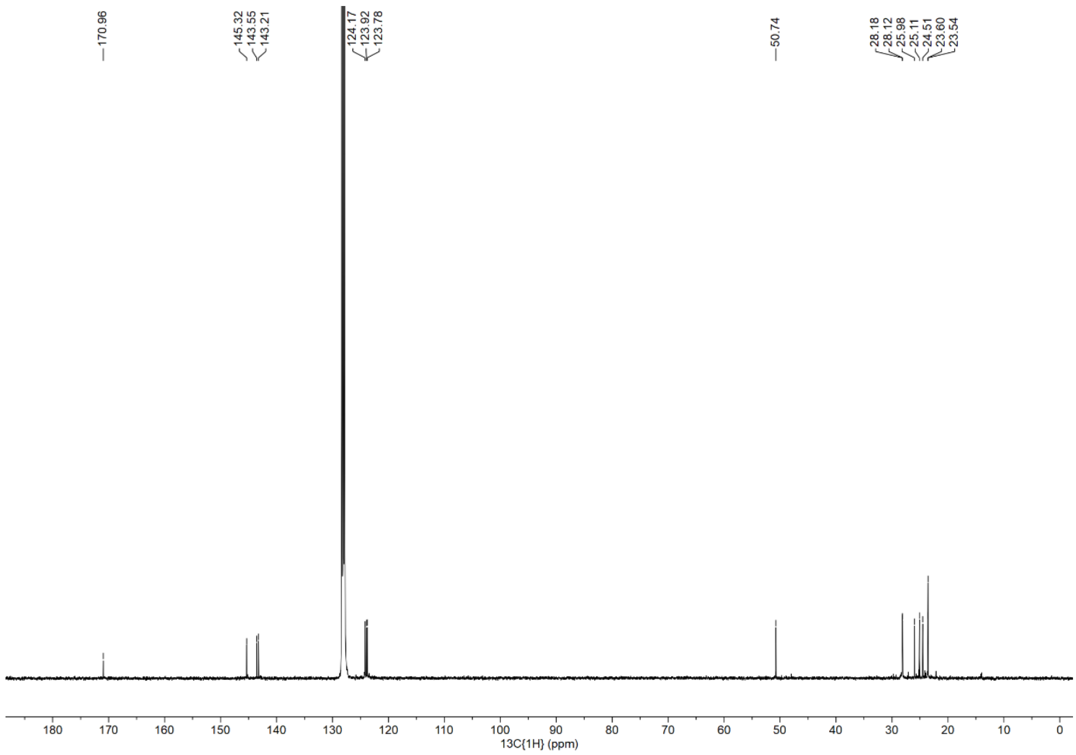

Figure S31. $^{13}C\{^{1}H\}$ NMR spectrum (101 MHz, 298 K, C_6D_6) of $[Mg(Priso)_2]$.

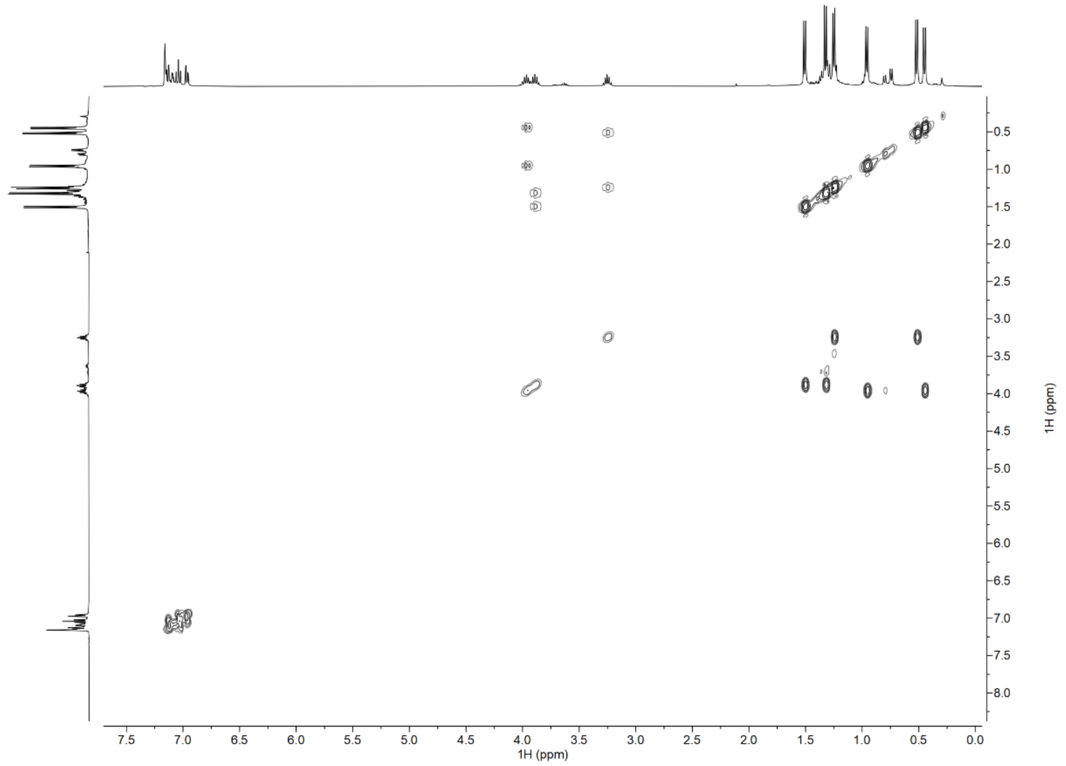

Figure S32. ^{1}H-COSY NMR spectrum (400 MHz, 298 K, C_6D_6) of $[Mg(Priso)_2]$.

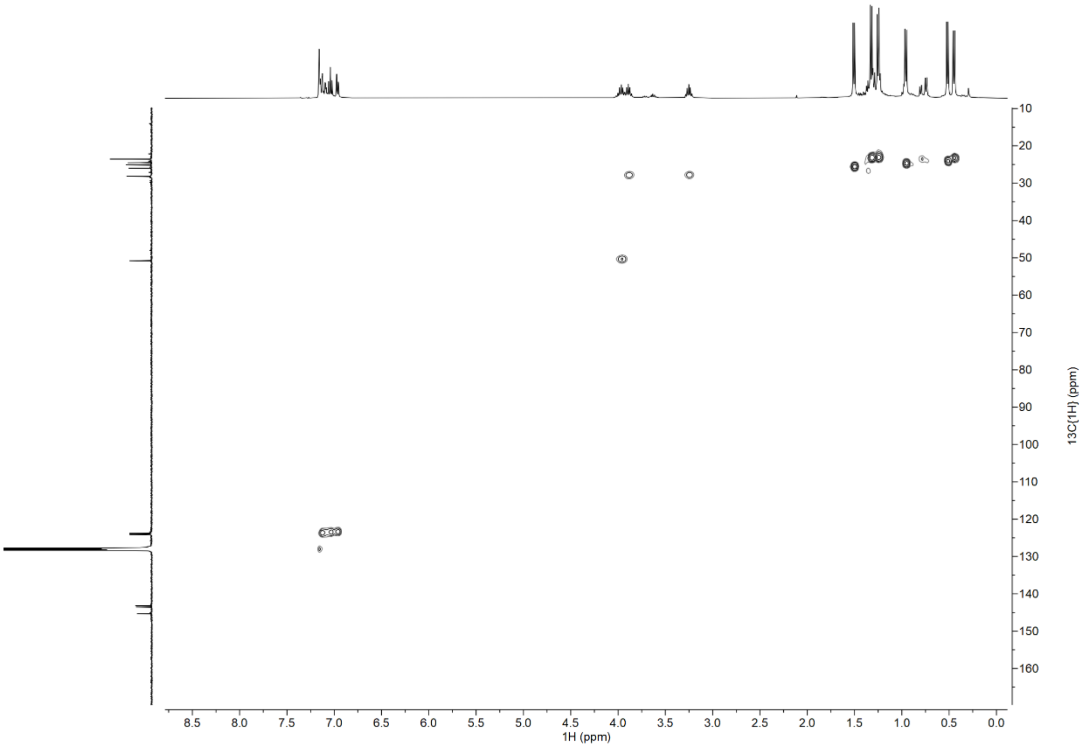

Figure S33. HMQC spectrum (^{1}H: 400 MHz; ^{13}C: 101 MHz, 298 K, C_6D_6) of [Mg(Priso)$_2$].

I_2 quench reactions involving compounds 5-9.

Compounds, **5-9** (*ca.* 15 mg) were generated *in situ* by UV light irradiation of solutions of **3** in the appropriate arene, as described above. Once reactions were complete, 70 μL of a solution of I_2 in THF (0.5 M) was added and the solution shaken. The ^{1}H and ^{13}C{^{1}H} NMR spectra of aliquots of these solutions were recorded in $CDCl_3$, and then samples of those solutions were analyzed by GC/MS. Comparison of the ^{1}H NMR spectra of quenched mixtures with those of known iodo-arenes[4-8] allowed identification of quenched products, and their relative proportions. For the quenches of compounds **8** and **9**, only one iodo-arene product was identified, i.e. 1-iodo-2,5-dimethylbenzene and 1-iodo-2,4,6-trimethylbenzene. The quenches of the product isomeric mixtures of **5-7** yielded the iodo-arene species shown below, with their relative proportions highlighted.

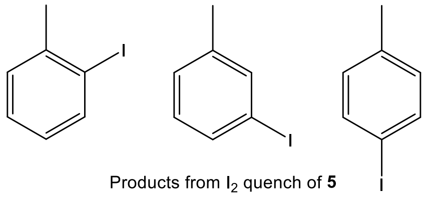

Products from I_2 quench of **5**
2:3:1 ratio

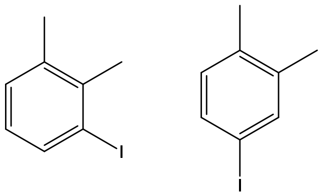

Products from I_2 quench of **6**
1.5:1 ratio

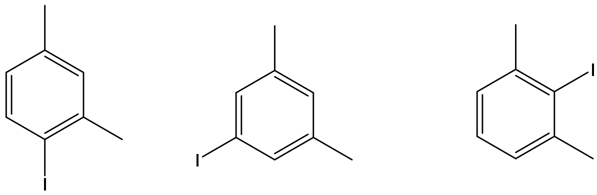

Products from I_2 quench of **7**
2:2:1 ratio

2. X-ray crystallography

Crystals suitable for X-ray structural determination were mounted in silicone oil. Crystallographic measurements were made using a Rigaku Xtalab Synergy Dualflex using a graphite monochromator with Cu Kα radiation (1.54180 Å). All structures were solved by direct methods and refined on F^2 by full matrix least squares (SHELX-16[9]) using all unique data. Hydrogen atoms, except hydride ligands, are typically included in calculated positions (riding model). Crystal data, details of data collections and refinements for all structures can be found in their CIF files and are summarized in Table S1.

Table S1. Summary of Crystallographic Data for Compounds **4**, **5**, **10**, **12** and **13**.

	**4**·(benzene)$_{0.5}$	**5**·(toluene)$_{0.5}$	**10**·(benzene)$_{0.25}$
empirical formula	$C_{71}H_{105}Mg_2N_6$	$C_{145}H_{216}Mg_4N_{12}$	$C_{59.50}H_{89.50}MgN_6$
formula weight	1091.22	2224.53	913.18
crystal system	Monoclinic	Monoclinic	Triclinic
space group	$P2_1/c$	$P2_1/c$	P-1
a (Å)	22.1452(2)	22.1443(6)	10.2656(2)
b (Å)	10.58860(10)	10.78930(10)	14.4708(2)
c (Å)	30.9866(2)	30.8526(7)	21.0517(3)
α (deg.)	90	90	79.0590(10)
β (deg)	110.5310(10)	109.668(3)	78.1960(10)
γ (deg.)	90	90	73.3330(10)
vol (Å^3)	6804.43(11)	6941.3(3)	2903.71(8)
Z	4	2	2
T (K)	123(2)	123(2)	123(2)
ρ (calcd) (g.cm^{-3})	1.065	1.064	1.044
μ (mm^{-1})	0.630	0.625	0.555
F(000)	2388	2436	1001
reflections collected	67247	76304	39432
unique reflections	12636	14527	11827
R_{int}	0.0253	0.0482	0.0342
R1 indices [I>2σ(I)][a]	0.0377	0.0579	0.0794
wR2 indices (all data)[b]	0.0994	0.1454	0.2467
Largest peak and hole (e.A^{-3})	0.359, -0.294	0.323, -0.271	1.060, -0.378
CCDC No.	2182705	2182703	2182702

Table S1 (contd.). Summary of Crystallographic Data for Compounds **4**, **5**, **10**, **12** and **13**.

	**12**·(hexane)	**13**·(hexane)$_{0.5}$
empirical formula	$C_{68}H_{108}Mg_2N_6$	$C_{63}H_{96}Mg_2N_5$
formula weight	1058.22	972.06
crystal system	Triclinic	Triclinic
space group	*P*-1	*P*-1
a (Å)	9.811(2)	12.5057(3)
b (Å)	10.608(2)	13.9605(3)
c (Å)	16.597(3)	21.0678(4)
α (deg.)	86.37(3)	92.058(2)
β (deg)	73.30(3)	105.147(2)
γ (deg.)	74.86(3)	115.965(2)
vol (Å^3)	1596.9(6)	3143.74(13)
Z	1	2
T (K)	123(2)	123(2)
ρ (calcd) (g.cm^{-3})	1.100	1.027
μ (mm^{-1})	0.654	0.624
F(000)	582	1066
reflections collected	29557	39377
unique reflections	5913	11632
R_{int}	0.0209	0.0539
R1 indices [$I>2\sigma(I)$][a]	0.0558	0.0447
*w*R2 indices (all data)[b]	0.1553	0.1216
Largest peak and hole (e.A^{-3})	0.883, -0.717	0.404, -0.310
CCDC No.	2182701	2182704

3. EPR spectroscopic studies

X-band EPR spectra were recorded on a Bruker ELEXSYS spectrometer with a cryogen-free in-cavity temperature control system at 77 K with 9.35 GHz microwave frequency. In a glovebox, a 4 mm thin wall EPR tube was charged with **3** (1.0 mg) and a 3:1 mixture of 2,2-dimethylbutane/tert-butylbenzene (0.15 mL). The tube was capped with a rubber septum. The sample was cooled to 77 K in a nitrogen-filled quartz finger dewar at which temperature the sample was photolyzed for 1 h using a 100 W Hg lamp equipped with a 335 nm longpass filter. X-band EPR spectra were recorded at 77 K.

Spectral simulations were performed using Easyspin (Version 5.2.32) and MATLAB (R2022a, Version 9.12.0.1927505). The isotropic spectra were simulated using "garlic".[10]

As is alluded to in the main text, the EPR signal of an irradiated glassed solution of **3** is broad and has poorly defined hyperfine structure (see Figure 8). Attempts to simulate the spectrum were not successful, but revealed that it is a composite of a two signals (*ca.* 40:1 ratio). The dominant signal is broad and unstructured. The less intense signal displays hyperfine structure that is consistent with the presence of a small amount of ligand radical, Priso· (Figure S34). The experimental spectrum was simulated using two components. A broad range of ^{25}Mg hfc values (A(^{25}Mg) 1-100 G) were examined for the major spectral feature and all gave broad central signals with little fine structure (Figures S34 and S35).

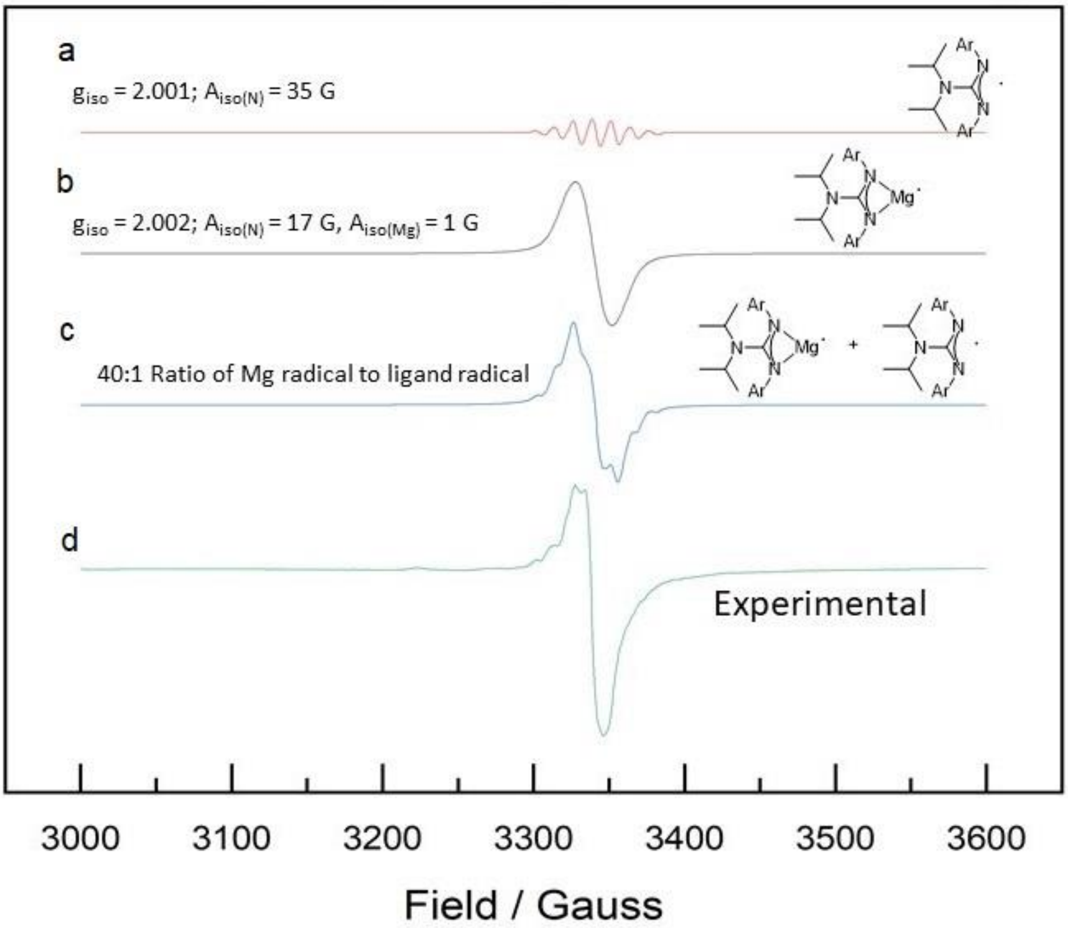

Figure S34. Simulated EPR spectra of (a) Priso· radical, (b) (Priso)Mg· radical and a 40:1 mixture of Priso· and (Priso)Mg· using the inset hyperfine couplings. (d) The experimental EPR spectrum of a solution of UV irradiated **3** (77 K).

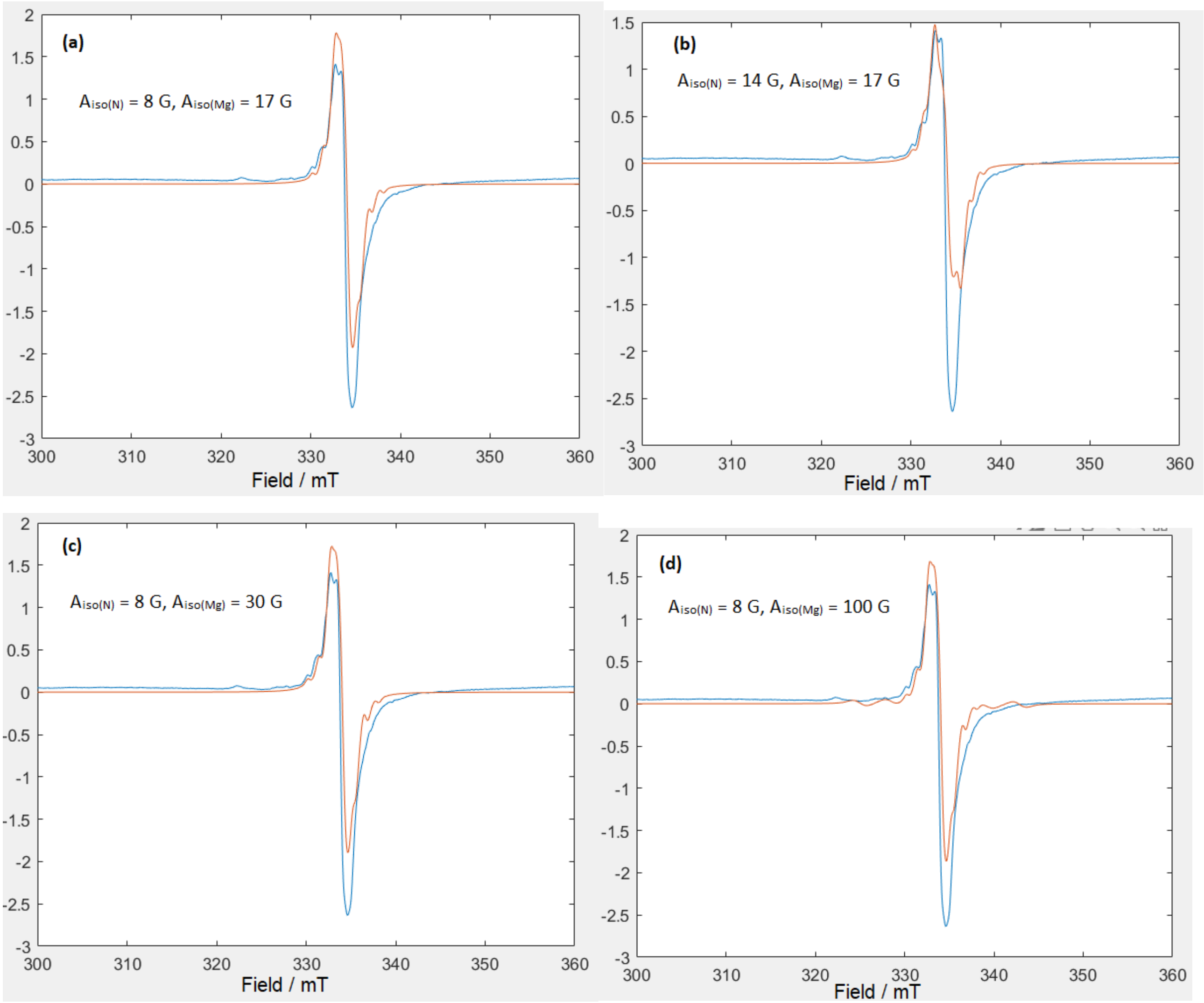

Figure S35. Simulated EPR spectra (red) of a 40:1 mixture of (Priso)Mg· and Priso· using a variety of hyperfine couplings to ^{25}Mg (17-100 G, see insets) for the former, and hyperfine couplings for the latter as shown in Figure S34. The simulated spectra overlay the experimental spectrum (blue) in each case.

4. Computational Studies

All calculations were performed using Gaussian16 suite of programs[11] using the Becke's 3-parameter hybrid functional[12] combined with the non-local correlation functional provided by Perdew/Wang(B3PW91).[13] The 6-311+G(d) all-electron basis set was used for the Mg atoms and the 6-31G(d,p) for the remaining atoms.[14] The nature of the extrema (minimum or transition states) was established with analytical geometry optimizations, and frequency calculations were computed without any symmetry constraints. Intrinsic Reaction Paths (IRPs)[15] were traced from the various transition structures to obtain the connected intermediates. The Gibbs free energy was computed at T=298 K in the gas phase. The molecular orbital diagrams were obtained using Multiwfn suite of programs[16] and VMD.[17]

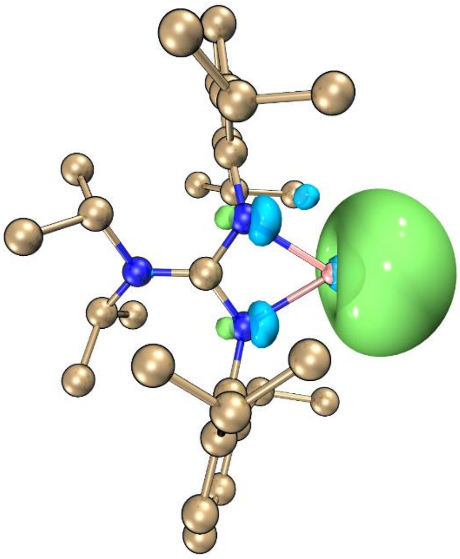

Figure S36. Calculated SOMO for the magnesium(I) radical, (Priso)Mg·.

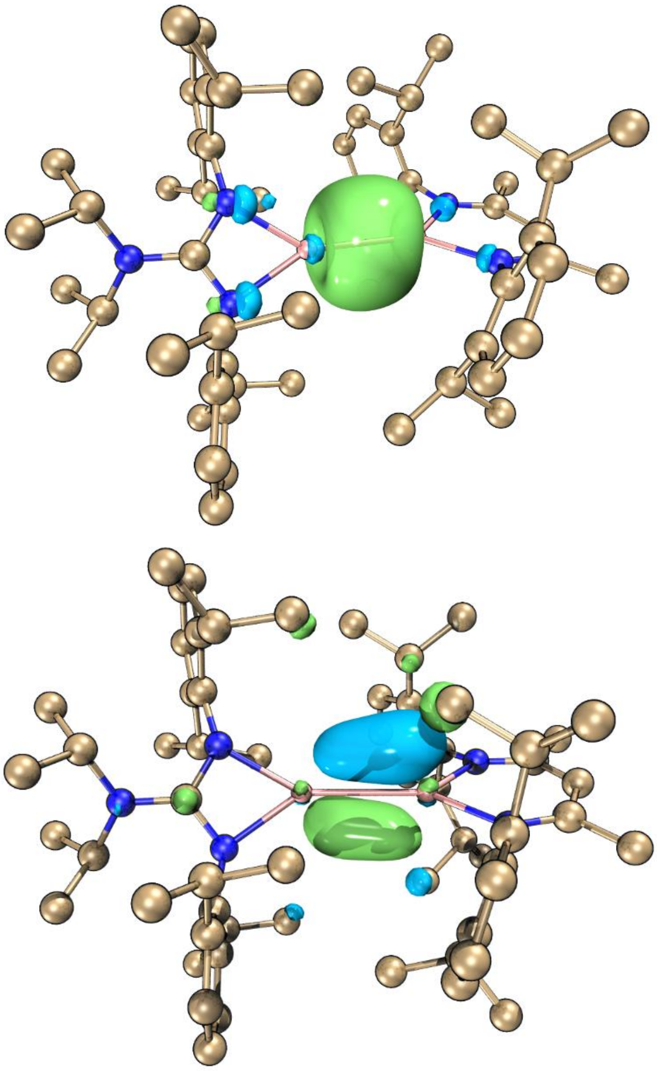

Figure S37. Calculated HOMO (top) and LUMO+1 (bottom) for the unsymmetrical dimagnesium(I) compound **13**.

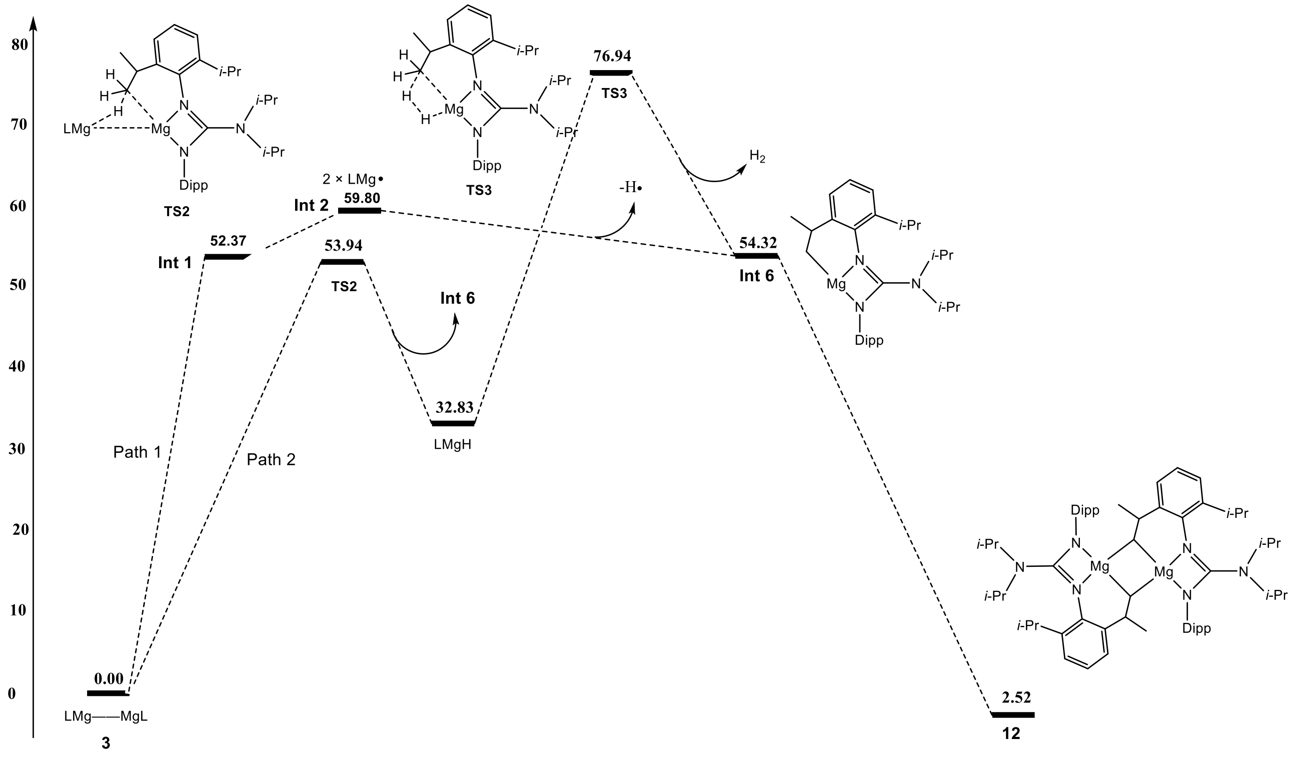

Figure S38. Two computed Gibbs free energy profiles for the formation of compound **12**, *via* intramolecular C–H activation of **3**. Radical Path 1 is thermodynamically more viable than non-radical Path 2. Energies are presented as kcal/mol.

Table S2. Cartesian coordinates of all optimized structures.

166

3

Mg	-1.38724300	0.00008700	-0.00164400
N	-3.13564300	0.74407800	0.83374500
C	-3.88672100	0.00092300	-0.00126100
N	-5.26633000	0.00167700	-0.00165300
C	-3.50611600	1.98746900	1.37017200
C	-3.75616500	3.09909100	0.53293600
C	-4.05995400	4.32688000	1.12509300
H	-4.26261200	5.18834900	0.49655100
C	-4.10589600	4.46879700	2.50873400
H	-4.35150700	5.43071900	2.94955800
C	-3.82167700	3.37802300	3.32345800
H	-3.84269400	3.49706400	4.40258700
C	-3.51281500	2.13333400	2.77351000
C	-3.61580700	2.97074000	-0.97288100
H	-3.94621000	1.96847700	-1.26166900
C	-2.14160400	3.10228700	-1.37656700
H	-1.77106900	4.10908700	-1.15698100
H	-2.00162900	2.90591700	-2.44487500
H	-1.50007100	2.40774600	-0.82067100
C	-4.46400300	3.96855000	-1.76174200
H	-5.50897100	3.95705500	-1.43661900
H	-4.43499000	3.72583000	-2.82876200
H	-4.09188700	4.99287800	-1.65317900

C	-3.13703400	0.94908500	3.64137500
H	-3.40503400	0.04604200	3.07956100
C	-3.86961200	0.90917800	4.98143100
H	-3.56074000	1.72662300	5.64194000
H	-3.65029700	-0.02735800	5.50451200
H	-4.95376100	0.97914700	4.84545100
C	-1.61587700	0.90956800	3.83832200
H	-1.09674300	0.87893400	2.87460300
H	-1.31399300	0.02711300	4.41348900
H	-1.27248900	1.80263700	4.37203700
C	-5.96489200	0.26789000	1.26940600
H	-5.15708000	0.32127000	1.99875300
C	-6.86153700	-0.89185700	1.70579800
H	-7.78999100	-0.93360000	1.13133700
H	-7.13510800	-0.75160800	2.75664300
H	-6.35021800	-1.85112300	1.60938500
C	-6.72738200	1.59246600	1.35547300
H	-6.15206300	2.41545600	0.93214500
H	-6.90815200	1.82535500	2.40946500
H	-7.69957700	1.54167500	0.85897500
N	-3.13623600	-0.74329800	-0.83603200
C	-3.50796400	-1.98725400	-1.37028600
C	-3.75956700	-3.09702400	-0.53095600
C	-4.06474200	-4.32555400	-1.12084200
H	-4.26868400	-5.18555700	-0.49071700
C	-4.11031200	-4.47012200	-2.50423800
H	-4.35697000	-5.43258900	-2.94328300
C	-3.82432400	-3.38132000	-3.32094700
H	-3.84491800	-3.50246000	-4.39985400
C	-3.51427400	-2.13590100	-2.77330800
C	-3.61924100	-2.96602500	0.97465600
H	-3.94968800	-1.96329800	1.26178000
C	-2.14504600	-3.09677600	1.37864700
H	-1.77463400	-4.10413400	1.16145200
H	-2.00501600	-2.89785300	2.44647000
H	-1.50337600	-2.40372200	0.82107500
C	-4.46727900	-3.96248800	1.76537700
H	-5.51230300	-3.95166000	1.44041900
H	-4.43812300	-3.71786700	2.83195600
H	-4.09508500	-4.98697400	1.65855200
C	-3.13664700	-0.95391200	-3.64338900
H	-3.40278800	-0.04944300	-3.08300000
C	-3.86975400	-0.91505800	-4.98321300
H	-3.56278300	-1.73432300	-5.64235200
H	-3.64888700	0.02009300	-5.50812100
H	-4.95397300	-0.98269500	-4.84660700
C	-1.61550100	-0.91749400	-3.84095900
H	-1.09600100	-0.88587900	-2.87747500
H	-1.31229300	-0.03669100	-4.41796000
H	-1.27386200	-1.81220200	-4.37305700
C	-5.96441600	-0.26370600	-1.27318600
H	-5.15614200	-0.31834600	-2.00191000
C	-6.85903100	0.89730700	-1.71036800
H	-7.78770200	0.94071800	-1.13638400
H	-7.13231600	0.75711500	-2.76129800
H	-6.34625200	1.85581500	-1.61407700
C	-6.72881800	-1.58715100	-1.35959400
H	-6.15502700	-2.41096900	-0.93579200
H	-6.90926200	-1.81985300	-2.41368300
H	-7.70127200	-1.53483800	-0.86375600
Mg	1.38702900	-0.00007900	-0.00126900
N	3.13630100	0.83480100	-0.74346000

C	3.88647000	-0.00092500	-0.00013000
N	5.26607000	-0.00162300	0.00038300
C	3.50824900	1.37174500	-1.98619200
C	3.75910300	0.53489900	-3.09795400
C	4.06465400	1.12747600	-4.32509900
H	4.26798700	0.49925100	-5.18664000
C	4.11151200	2.51116400	-4.46627200
H	4.35851600	2.95233000	-5.42768200
C	3.82645700	3.32551500	-3.37544200
H	3.84819400	4.40468800	-3.49394900
C	3.51586200	2.77514300	-2.13136900
C	3.61766800	-0.97089300	-2.97045900
H	3.94698100	-1.26041300	-1.96804300
C	2.14330700	-1.37354800	-3.10348700
H	1.77386900	-1.15352200	-4.11059400
H	2.00240300	-2.44179200	-2.90745200
H	1.50151200	-0.81731900	-2.40946100
C	4.46619600	-1.75984300	-3.96790400
H	5.51141500	-1.43556400	-3.95522200
H	4.43611000	-2.82697900	-3.72582700
H	4.09513900	-1.65038300	-4.99252200
C	3.13937100	3.64262200	-0.94707900
H	3.40596800	3.08009700	-0.04405400
C	3.87289500	4.98212100	-0.90563500
H	3.56541000	5.64330500	-1.72305800
H	3.65293500	5.50484800	0.03094800
H	4.95702100	4.84538400	-0.97448500
C	1.61832300	3.84072900	-0.90913900
H	1.09837700	2.87742000	-0.87946600
H	1.31596900	4.41575700	-0.02675200
H	1.27631500	4.37511400	-1.80233400
C	5.96517500	1.26952200	-0.26396100
H	5.15761400	1.99908200	-0.31774800
C	6.86049900	1.70491600	0.89718700
H	7.78877600	1.13024100	0.93974500
H	7.13445000	2.75579800	0.75794700
H	6.34796000	1.60802500	1.85575700
C	6.72921800	1.35634900	-1.58756900
H	6.15459200	0.93397500	-2.41154500
H	6.91074500	2.41045100	-1.81936500
H	7.70112300	0.85934400	-1.53609800
N	3.13504100	-0.83596200	0.74279800
C	3.50554300	-1.37202600	1.98633300
C	3.75631100	-0.53430400	3.09746100
C	4.06050900	-1.12592900	4.32539600
H	4.26377100	-0.49702600	5.18645700
C	4.10605900	-2.50951900	4.46799700
H	4.35200600	-2.94992800	5.43002400
C	3.82101600	-3.32469600	3.37778600
H	3.84169600	-4.40377000	3.49737200
C	3.51179500	-2.77528700	2.13294200
C	3.61611500	0.97147700	2.96840600
H	3.94684600	1.25985400	1.96613000
C	2.14189700	1.37536200	3.09927300
H	1.77111500	1.15662600	4.10616500
H	2.00202000	2.44350900	2.90197800
H	1.50048900	0.81890900	2.40508100
C	4.46398500	1.76079600	3.96612400
H	5.50900200	1.43581100	3.95502900
H	4.43491000	2.82771800	3.72298900
H	4.09160500	1.65255900	4.99039200
C	3.13539000	-3.64366000	0.94928200

H	3.40305500	-3.08226300	0.04587200
C	3.86781800	-4.98381300	0.90968400
H	3.55919900	-5.64393200	1.72754100
H	3.64807100	-5.50727900	-0.02653600
H	4.95201100	-4.84792500	0.97915900
C	1.61420300	-3.84053100	0.91056500
H	1.09510800	-2.87680400	0.87957100
H	1.31188900	-4.41621400	0.02859200
H	1.27117900	-4.37367800	1.80410900
C	5.96367500	-1.27351300	0.26517900
H	5.15529500	-2.00219800	0.31844500
C	6.85926200	-1.70983300	-0.89542000
H	7.78816300	-1.13612000	-0.93741700
H	7.13204100	-2.76100000	-0.75602400
H	6.34740400	-1.61240500	-1.85429800
C	6.72676900	-1.36120000	1.58927900
H	6.15211400	-0.93818100	2.41290500
H	6.90693700	-2.41551200	1.82117400
H	7.69928600	-0.86532300	1.53844500

166
Int1

C	-3.36273000	-3.11913500	1.50424300
C	-3.07492700	-2.72466200	0.17381900
C	-2.82954100	-3.70658300	-0.81014300
C	-2.96674800	-5.05547100	-0.47816500
C	-3.31972400	-5.44659300	0.80744300
C	-3.49546600	-4.47856400	1.79205600
N	-2.90437000	-1.36965300	-0.15381400
C	-2.36104900	-3.30363900	-2.19389200
C	-3.16408700	-3.96172300	-3.31825900
C	-3.41664100	-2.08873500	2.61574900
C	-2.00069900	-1.81175900	3.13846300
C	-3.84739100	-0.40225600	-0.06679000
N	-3.29832300	0.77465500	0.26041400
C	-3.71149900	2.10115400	0.10846100
C	-3.81264200	2.77151300	-1.13434800
C	-4.06093200	4.15203700	-1.11978700
C	-4.19118600	4.87864700	0.05314800
C	-4.06594500	4.21626200	1.27059200
C	-3.82762100	2.84634600	1.31555500
C	-3.64142900	2.19737700	-2.54124300
C	-4.96976600	2.19587900	-3.30981000
C	-3.66344700	2.15608800	2.65682600
C	-4.70852600	2.59006600	3.68627400
C	-2.95528500	0.84678100	-2.70302800
C	-2.24596700	2.34070000	3.20214500
C	-0.86363900	-3.60035900	-2.33767000
C	-4.33310800	-2.46958900	3.77855200
Mg	-1.34501900	-0.03182500	0.25579300
Mg	2.37156800	-1.52315800	0.33444600
N	2.54691600	0.45228000	-0.34460700
C	3.86610800	0.49898400	-0.06121100
N	4.30400700	-0.67468200	0.43046200
C	5.56368300	-1.24900300	0.21744000
C	6.09033900	-1.41859700	-1.08795100
C	7.34135500	-2.01759100	-1.23172100
C	8.05539800	-2.47990400	-0.12999200
C	7.49191300	-2.38789200	1.13706500
C	6.24448000	-1.79127000	1.33021800
C	5.27505400	-1.03385800	-2.30967700

C	6.11643600	-0.72067600	-3.54709900
C	5.59300800	-1.79516800	2.69892500
C	6.55726000	-1.45939500	3.83745400
C	1.60970100	1.49091700	-0.33016200
C	0.86467300	1.72443200	-1.51200400
C	-0.19742900	2.63303500	-1.47152600
C	-0.52949100	3.30540100	-0.29700200
C	0.22873600	3.08841300	0.84995800
C	1.29456600	2.18528000	0.86146400
C	1.24161300	0.98379700	-2.78314300
C	0.64375900	-0.42590700	-2.83011900
C	2.05863200	1.89245200	2.13820300
C	1.43114800	0.72653900	2.91020900
C	0.89933600	1.75445600	-4.05742100
C	2.21173100	3.10864000	3.05162800
C	4.26059600	-2.13787100	-2.63160200
C	4.90611800	-3.14596300	2.93909700
H	9.03367900	-2.93250300	-0.26382300
H	7.76819500	-2.13133000	-2.22300100
H	8.02908400	-2.78818000	1.99205700
H	4.80405600	-1.03574500	2.68064000
H	-4.15033400	4.77513700	2.19819000
H	3.06294500	1.57930200	1.84939700
H	4.70767800	-0.13025300	-2.07599200
H	-4.12625800	4.67184700	-2.07345200
H	-2.78913200	-5.81096400	-1.23898000
H	-0.02390300	3.62680500	1.75640200
H	-3.72960700	-4.78861000	2.80587600
H	-2.48980200	-2.21804200	-2.26722200
H	2.32987800	0.84674500	-2.74997700
H	-3.79295900	-1.15466800	2.19304400
H	-0.77212700	2.82692200	-2.37021500
H	-3.78144800	1.08511500	2.47408800
H	-2.99570000	2.92729400	-3.05134900
H	6.01142400	-1.36689500	4.78232100
H	7.31143600	-2.24139800	3.97617300
H	7.08217000	-0.51765800	3.65148200
H	4.38664700	-3.15707500	3.90410300
H	4.17045300	-3.35799800	2.15466500
H	5.63972700	-3.95997700	2.93447100
H	3.59975400	-1.84035700	-3.45272000
H	4.77441300	-3.06296400	-2.91389800
H	3.63079100	-2.37587000	-1.76484400
H	5.47968600	-0.31792600	-4.34163400
H	6.89833600	0.01394300	-3.32979000
H	6.60180700	-1.61677600	-3.94791600
H	-4.37270000	5.94887500	0.02065600
H	-2.12376300	1.81968100	4.15855400
H	-1.50920800	1.94305400	2.49818900
H	-2.01930300	3.40167100	3.35883300
H	-4.63990700	1.96373500	4.58213000
H	-4.56127100	3.62752000	4.00507100
H	-5.72317200	2.50475800	3.28624100
H	-4.80489600	1.92996100	-4.35994700
H	-5.65778600	1.46395600	-2.88259800
H	-5.45880000	3.17318700	-3.27771000
H	-2.71294700	0.69047800	-3.76017100
H	-2.01874500	0.80253500	-2.14337800
H	-3.58222000	0.01556100	-2.38303600
H	-1.37170700	3.98894100	-0.27696500
H	1.31418500	1.24117100	-4.93072500
H	-0.18331800	1.82488800	-4.20897300

H	1.30373900	2.77130800	-4.03328400
H	1.03535000	-0.98497700	-3.68666700
H	0.88872900	-0.99057700	-1.92480600
H	-0.44569700	-0.39697000	-2.92064000
H	-0.48414600	-3.25377400	-3.30505000
H	-0.29030600	-3.10724100	-1.54559100
H	-0.67150900	-4.67633500	-2.26114100
H	-2.84025700	-3.58124700	-4.29296900
H	-3.02809800	-5.04838000	-3.32990900
H	-4.23587300	-3.76406700	-3.21600700
H	-2.00485700	-1.00612900	3.88106400
H	-1.57884800	-2.71079800	3.60046900
H	-1.31534100	-1.52108900	2.33021400
H	-4.45986400	-1.61617900	4.45246700
H	-5.32320900	-2.77982300	3.43193600
H	-3.91542300	-3.29061100	4.37104800
H	-3.43384200	-6.49933300	1.04933600
H	2.06897500	0.42599600	3.74837500
H	0.44795100	1.00097200	3.30511500
H	1.27249600	-0.14960100	2.26691200
H	2.87412300	2.86759800	3.88948100
H	2.63618200	3.96379400	2.51647600
H	1.25288100	3.42020600	3.47916300
N	4.64692300	1.61354800	-0.27883800
C	5.88006200	1.96345600	0.46054400
H	5.96079800	3.04085000	0.29069700
C	4.20699600	2.62610200	-1.25819400
H	3.32575900	2.20761000	-1.73878200
C	5.73376900	1.78496200	1.96611700
H	6.67181700	2.06122000	2.45709900
H	4.94059400	2.42209800	2.36426000
H	5.51234100	0.74942500	2.22050300
C	7.19455400	1.36317600	-0.05379700
H	7.40327900	0.38401500	0.37501500
H	7.19393300	1.26339900	-1.13984700
H	8.01370400	2.03516500	0.22586000
C	3.79660800	3.95466300	-0.62365700
H	3.04317800	3.80315500	0.14952500
H	4.64946300	4.48765800	-0.19016500
H	3.36329300	4.60408900	-1.39029700
C	5.25259700	2.84372600	-2.35247600
H	5.52078900	1.90292300	-2.83731900
H	4.84482500	3.51717200	-3.11283500
H	6.16617000	3.30388400	-1.96341200
N	-5.17955300	-0.63188400	-0.36908700
C	-6.35128700	-0.02575200	0.30092800
H	-7.17997200	-0.63334200	-0.07139800
C	-5.51112000	-1.78080400	-1.23804500
H	-4.55714300	-2.14567500	-1.60937200
C	-6.32483400	-0.23611800	1.80941300
H	-7.25822100	0.12396600	2.25388800
H	-6.21433800	-1.29492200	2.04883500
H	-5.50455300	0.31114700	2.27358000
C	-6.71760200	1.41092400	-0.06666600
H	-6.08400100	2.15042200	0.42012400
H	-6.66193400	1.57329700	-1.14323700
H	-7.75092100	1.59121400	0.24994200
C	-6.20268300	-2.93644600	-0.51346800
H	-5.65041600	-3.23136000	0.37931800
H	-7.23160000	-2.68798100	-0.23033100
H	-6.24793200	-3.80387200	-1.17864300
C	-6.32096000	-1.33520000	-2.45547500

H	-5.76697900	-0.60837600	-3.05220200
H	-6.53553300	-2.20187000	-3.08877800
H	-7.28167600	-0.89030300	-2.17552900

178

Int1a

C	1.16498100	-0.79544100	-3.29179400
C	-0.06496300	-1.40623000	-3.26354800
C	-1.28610700	-0.64398200	-3.15975400
C	-1.16352100	0.79480600	-3.29252000
C	0.06645000	1.40555200	-3.26422000
C	1.28741300	0.64334000	-3.16006800
H	-0.12327800	-2.48828700	-3.33967100
H	2.05946900	-1.40624600	-3.38134700
H	2.22129000	1.12717100	-3.43336500
H	0.12484200	2.48757200	-3.34091900
H	-2.05796300	1.40562400	-3.38260400
H	-2.21967200	-1.12776100	-3.43436600
C	3.13525700	2.50175900	2.06420200
C	3.40002600	2.29117500	0.68863500
C	3.70649000	3.39500000	-0.13846700
C	3.73214300	4.67826700	0.41107000
C	3.45835700	4.89048100	1.75567200
C	3.16697400	3.80094800	2.57135000
N	3.19253000	1.05097300	0.06535100
C	3.98147200	3.20094900	-1.61495900
C	5.38577700	3.67221600	-2.00342700
C	2.80154500	1.32240100	2.95033600
C	1.35383400	0.87203300	2.77822500
C	3.75758300	-0.16084600	0.25789100
N	2.98147200	-1.12813400	-0.25849900
C	3.26226100	-2.43845800	-0.66575600
C	4.12856900	-2.78448600	-1.73796800
C	4.25292200	-4.14226800	-2.06917500
C	3.56651200	-5.14559900	-1.40628400
C	2.65726900	-4.79082700	-0.41746400
C	2.46870400	-3.45850900	-0.06265500
C	4.91086700	-1.86157400	-2.68264000
C	6.41511400	-2.17490500	-2.68698600
C	1.31199800	-3.11550600	0.86239000
C	1.20900500	-4.01169200	2.09832400
C	4.72800700	-0.35365700	-2.56868200
C	0.00471100	-3.18284300	0.06207900
C	2.90802300	3.88253300	-2.46835100
C	3.10275900	1.54566000	4.43166500
Mg	1.53266700	0.22862700	-0.88083000
H	2.06022800	-5.55945000	0.06445100
H	4.90166200	-4.41401400	-2.89767100
H	3.96539300	5.52425100	-0.23065400
H	2.96079500	3.96563600	3.62416000
H	3.92453500	2.12597800	-1.80322800
H	3.43296400	0.49975000	2.61329100
H	1.44243500	-2.08038700	1.19539900
H	4.53977800	-2.13620900	-3.68200200
H	3.70509800	-6.18575100	-1.68623300
H	-0.85295100	-2.89241700	0.67568900
H	0.05901800	-2.53132300	-0.81866300
H	-0.17931900	-4.20008500	-0.29973600
H	0.39068100	-3.67014700	2.74000700
H	0.98553800	-5.04905400	1.82879200
H	2.13332500	-4.00818500	2.68228200
H	6.91833800	-1.59114000	-3.46545000

H	6.86707200	-1.91363600	-1.72815600
H	6.62561300	-3.23064400	-2.87069800
H	5.19398000	0.13068900	-3.43361500
H	3.68195400	-0.05917400	-2.54550100
H	5.20138200	0.04841400	-1.67397700
H	2.92418500	4.96985300	-2.33479600
H	5.58010200	3.47071500	-3.06252200
H	5.50845600	4.74860100	-1.84059100
H	6.15283600	3.15879800	-1.41490000
H	1.17355000	-0.04601200	3.34359900
H	0.65941500	1.63897800	3.13623400
H	1.10925100	0.66767900	1.73098000
H	2.97670700	0.60753900	4.98242700
H	4.12568800	1.89951900	4.59372000
H	2.41879400	2.27463400	4.87978300
H	3.47526500	5.89461300	2.16939200
N	4.98425800	-0.34844500	0.87252900
C	5.34628400	-1.51770000	1.70545900
H	6.15119200	-1.12782400	2.33560000
C	5.97941900	0.74440700	0.84319400
H	5.55623200	1.50836100	0.19042300
C	4.22468700	-1.93973100	2.64973200
H	4.50464900	-2.86782100	3.15658600
H	4.03299600	-1.18349500	3.41376700
H	3.30157100	-2.12165500	2.10192400
C	5.93762000	-2.73263400	0.98477900
H	5.17485700	-3.36810800	0.53829700
H	6.63575800	-2.43539900	0.20265500
H	6.48936100	-3.33173100	1.71781800
C	6.24843500	1.39387200	2.20115000
H	5.33489000	1.77194100	2.65425700
H	6.73360100	0.70363100	2.89927000
H	6.92463000	2.24295400	2.06335800
C	7.30055400	0.28937200	0.22113900
H	7.15511800	-0.11881700	-0.77977000
H	7.97809100	1.14542400	0.14312800
H	7.79929800	-0.46723900	0.83503000
H	3.06206000	3.67311300	-3.53286500
H	1.91112100	3.53124600	-2.18898900
C	-3.13593100	-2.50180200	2.06412800
C	-3.40040300	-2.29112700	0.68849800
C	-3.70611700	-3.39498900	-0.13885800
C	-3.73166300	-4.67830800	0.41055500
C	-3.45846300	-4.89057000	1.75527000
C	-3.16762300	-3.80104400	2.57114300
N	-3.19324200	-1.05086900	0.06529100
C	-3.98061500	-3.20090700	-1.61543400
C	-5.38503000	-3.67168400	-2.00415700
C	-2.80245700	-1.32248500	2.95041700
C	-1.35522800	-0.87091000	2.77741600
C	-3.75807900	0.16100500	0.25780500
N	-2.98176500	1.12820400	-0.25845600
C	-3.26227900	2.43843300	-0.66613500
C	-4.12849500	2.78444500	-1.73845600
C	-4.25261500	4.14216700	-2.06991700
C	-3.56602000	5.14552700	-1.40723700
C	-2.65681400	4.79077400	-0.41839600
C	-2.46850700	3.45848300	-0.06331900
C	-4.91110600	1.86157900	-2.68287500
C	-6.41537800	2.17466800	-2.68628400
C	-1.31182600	3.11547800	0.86174400
C	-1.20876200	4.01186900	2.09753300

C	-4.72766600	0.35370100	-2.56948600
C	-0.00454500	3.18262100	0.06141400
C	-2.90723300	-3.88280100	-2.46866800
C	-3.10241300	-1.54634400	4.43189100
Mg	-1.53376900	-0.22910700	-0.88175900
H	-2.05959700	5.55935600	0.06336400
H	-4.90131200	4.41385300	-2.89848100
H	-3.96441800	-5.52428900	-0.23135000
H	-2.96176800	-3.96577400	3.62401000
H	-3.92334300	-2.12596600	-1.80376400
H	-3.43473800	-0.50020100	2.61407700
H	-1.44235100	2.08043200	1.19494000
H	-4.54062300	2.13652800	-3.68237100
H	-3.70445400	6.18564500	-1.68738600
H	0.85311100	2.89221600	0.67506800
H	-0.05888900	2.53112900	-0.81930600
H	0.17959200	4.19987600	-0.30036400
H	-0.39044800	3.67040400	2.73927200
H	-0.98525100	5.04919200	1.82787200
H	-2.13308500	4.00846400	2.68149000
H	-6.91901500	1.59103000	-3.46458200
H	-6.86674900	1.91318800	-1.72725700
H	-6.62602100	3.23044700	-2.86965300
H	-5.19385800	-0.13051300	-3.43438100
H	-3.68144800	0.05969700	-2.54705400
H	-5.20035500	-0.04903400	-1.67471900
H	-2.92356100	-4.97010900	-2.33503600
H	-5.57904100	-3.47030400	-3.06333200
H	-5.50812200	-4.74799300	-1.84111600
H	-6.15201100	-3.15785200	-1.41589200
H	-1.17548100	0.04743000	3.34247700
H	-0.65993900	-1.63717100	3.13521100
H	-1.11137000	-0.66654300	1.73001600
H	-2.97646600	-0.60830700	4.98281700
H	-4.12502800	-1.90081400	4.59462800
H	-2.41769800	-2.27507600	4.87926100
H	-3.47534800	-5.89473400	2.16891000
N	-4.98474400	0.34873600	0.87250200
C	-5.34645000	1.51796000	1.70562500
H	-6.15120300	1.12811000	2.33599100
C	-5.97995500	-0.74405600	0.84316700
H	-5.55678200	-1.50804700	0.19042400
C	-4.22458300	1.93995700	2.64961300
H	-4.50473200	2.86769700	3.15701300
H	-4.03221600	1.18347800	3.41323500
H	-3.30179500	2.12250400	2.10149200
C	-5.93790600	2.73299800	0.98522200
H	-5.17524700	3.36814000	0.53809000
H	-6.63675000	2.43594200	0.20366100
H	-6.48888900	3.33239400	1.71858900
C	-6.24904100	-1.39367400	2.20107200
H	-5.33547900	-1.77138100	2.65443400
H	-6.73472900	-0.70372400	2.89910900
H	-6.92479400	-2.24306300	2.06303500
C	-7.30106800	-0.28901700	0.22110300
H	-7.15562200	0.11875600	-0.77996900
H	-7.97877800	-1.14497000	0.14348500
H	-7.79960300	0.46790300	0.83477200
H	-3.06118700	-3.67348300	-3.53321300
H	-1.91029200	-3.53162300	-2.18930200

83
Int2

C	2.59752800	1.76495900	-0.17988400
C	2.36248400	0.39569300	-0.43909600
C	3.44557400	-0.50534200	-0.53696800
C	4.74234400	-0.03196200	-0.33632600
C	4.98031500	1.31051800	-0.05933500
C	3.91223900	2.19824700	0.00723300
N	1.07252000	-0.07419800	-0.74106700
C	3.17743900	-1.95195400	-0.90108500
C	4.11699100	-2.94269300	-0.21487100
C	1.45195300	2.75963700	-0.17117500
C	1.30450700	3.40522200	-1.55450300
C	-0.01549900	-0.09931600	0.06498900
N	-1.14458900	0.05220800	-0.65728200
C	-2.43570600	-0.42368300	-0.40498200
C	-2.74126600	-1.80628500	-0.38197300
C	-4.04681600	-2.19006400	-0.05820700
C	-5.05380300	-1.25843900	0.15915900
C	-4.77945100	0.09062000	-0.04297700
C	-3.49123900	0.52263900	-0.35491500
C	-1.78508100	-2.92324900	-0.77919700
C	-0.86707100	-3.40404000	0.34261100
C	-3.24122700	1.96387900	-0.76025300
C	-4.05966300	2.98807700	0.02597100
C	-0.96061900	-2.64499900	-2.04284000
C	-3.49678400	2.10874500	-2.26722700
C	3.20580900	-2.12229500	-2.42470700
C	1.58299800	3.83485400	0.90888900
Mg	-0.08922600	0.25922100	-2.44455600
H	-5.58267900	0.81726600	0.03262100
H	-4.27722800	-3.25181600	-0.00228500
H	5.57938200	-0.72087500	-0.39963300
H	4.10387200	3.24831800	0.20781300
H	2.15408800	-2.17687700	-0.57810600
H	0.52916700	2.21008200	0.03251800
H	-2.17814000	2.17101600	-0.59990100
H	-2.44558400	-3.76335700	-1.02891900
H	-6.05607600	-1.58337200	0.42217800
H	-3.27757900	3.12726000	-2.60656000
H	-2.87032300	1.41813800	-2.84383800
H	-4.54192500	1.88274100	-2.50525500
H	-3.74347400	4.00349200	-0.23391100
H	-5.12811300	2.91593000	-0.20351400
H	-3.93896200	2.85815700	1.10532800
H	-0.31319300	-4.29690800	0.03166400
H	-0.14458500	-2.62864400	0.59556900
H	-1.43690400	-3.65010800	1.24354100
H	-0.63070900	-3.58657200	-2.49422700
H	-1.54648300	-2.10825600	-2.79853600
H	-0.04750200	-2.08578800	-1.80402700
H	4.20002700	-1.88888400	-2.82101900
H	3.79638900	-3.96982400	-0.41685900
H	5.14670800	-2.84928500	-0.57613700
H	4.12788300	-2.79580800	0.87000300
H	0.43635500	4.07253200	-1.58970700
H	2.19864000	3.98512100	-1.80777000
H	1.18202200	2.65077200	-2.34348300
H	0.67469500	4.44519900	0.94579100
H	1.73755900	3.39314200	1.89807000
H	2.42000000	4.51257900	0.71165600
H	5.99529200	1.66515800	0.09503000

N	0.05815100	-0.27084800	1.43199900
C	-0.92374400	0.22789200	2.41991500
H	-0.34604900	0.23728700	3.34772800
C	1.29048100	-0.81305200	2.04733300
H	1.87928100	-1.21642600	1.22669400
C	-1.32452200	1.67388900	2.17330200
H	-1.98152600	2.01385700	2.97980100
H	-0.44573400	2.32239500	2.14886600
H	-1.86260200	1.78015200	1.23555000
C	-2.12643900	-0.67845300	2.69440200
H	-2.92700500	-0.54990800	1.96847300
H	-1.83283400	-1.72948000	2.69944900
H	-2.52869000	-0.43353700	3.68364800
C	2.13033800	0.24690700	2.76251200
H	2.34591100	1.09219100	2.11152300
H	1.63506900	0.61222100	3.66875300
H	3.08557800	-0.19142000	3.06639800
C	1.00319000	-1.96803100	3.01170200
H	0.42595500	-2.76633800	2.54595600
H	1.95664600	-2.38873400	3.34546400
H	0.46730300	-1.63405200	3.90554900
H	2.95307000	-3.14920000	-2.71119700
H	2.48959000	-1.44785100	-2.90661700

178

Int3

C	0.37508900	-0.72343900	-1.38458700
C	-0.17091400	-1.61871400	-0.32268200
C	-0.19097200	-1.08714900	0.94854900
C	0.21360500	0.32245200	1.07576300
C	-0.32638200	1.15293900	-0.01075700
C	-0.31480800	0.59837900	-1.27102800
H	-0.40645800	-2.66440300	-0.51225200
H	-0.43838600	-1.68310600	1.82442200
H	0.18711700	0.75413800	2.07116700
H	-0.70773100	2.15058600	0.19692500
H	-0.67280700	1.13584200	-2.14607800
H	0.36625700	-1.14964100	-2.38518900
C	4.25100200	-3.23090600	0.30113600
C	4.12917100	-2.13949500	1.19133200
C	4.12094200	-2.36390200	2.58733700
C	4.26189400	-3.66495200	3.06935600
C	4.39542300	-4.74237500	2.19882800
C	4.38074500	-4.51914000	0.82702800
N	3.86839600	-0.84134500	0.72497400
C	3.91648700	-1.19487300	3.53073200
C	4.75112300	-1.28781200	4.80764500
C	4.18525500	-3.03433300	-1.20184500
C	2.79987200	-3.43057200	-1.72559400
C	4.67252800	-0.01375300	0.02205000
N	3.94200900	0.83000300	-0.73522000
C	4.26227900	2.10971500	-1.19824500
C	4.45655700	3.21015900	-0.32628200
C	4.84611300	4.43439600	-0.87952100
C	4.96764200	4.61780000	-2.25113100
C	4.59863400	3.58117900	-3.10249700
C	4.21612900	2.33885700	-2.59871800
C	4.16536800	3.20562000	1.16871900
C	5.29925300	2.67391700	2.04157700
C	3.62956800	1.28936000	-3.52502500
C	4.32711600	1.18254000	-4.88120100
C	2.84825800	2.52343900	1.54952800

C	2.13408700	1.58164000	-3.70805600
C	2.42563700	-1.02787300	3.84529500
C	5.27812500	-3.79592700	-1.95620300
Mg	2.14685700	-0.07826400	-0.16935700
H	4.59790500	3.74404600	-4.17626200
H	5.03238900	5.27023700	-0.20837000
H	4.26405200	-3.84053800	4.14128100
H	4.47111800	-5.36272100	0.14808300
H	4.22285800	-0.29248300	2.98984000
H	4.32349900	-1.96934300	-1.40935300
H	3.70279200	0.32302900	-3.01573000
H	4.04597400	4.26746000	1.42062200
H	5.28572200	5.57543000	-2.65280800
H	1.64792500	0.79373800	-4.29433700
H	1.62881000	1.64509700	-2.74112500
H	1.98970300	2.53761800	-4.22406400
H	3.92284900	0.33698400	-5.44760800
H	4.17099600	2.08043600	-5.48883000
H	5.40523300	1.03590200	-4.77146800
H	5.07658700	2.83687300	3.10219700
H	5.42571800	1.60332600	1.88413000
H	6.24599300	3.17094800	1.80923500
H	2.51057000	2.87608000	2.53019600
H	2.05608600	2.74254700	0.82718400
H	2.96633000	1.43900800	1.63469900
H	2.03502400	-1.91700900	4.35310800
H	4.65893000	-0.36362400	5.38755400
H	4.42308200	-2.10905300	5.45402300
H	5.81087700	-1.44404100	4.58097200
H	2.71735600	-3.23675100	-2.80086300
H	2.61399400	-4.49648400	-1.55245000
H	2.01164400	-2.86655300	-1.22151600
H	5.25161200	-3.54021500	-3.02079800
H	6.27464600	-3.55863400	-1.57269300
H	5.14232300	-4.87978600	-1.87908500
H	4.50365300	-5.75077900	2.58803600
N	6.05389300	-0.04735400	0.09546600
C	6.97762800	0.31709900	-0.99898600
H	7.89581300	-0.21046700	-0.72648700
C	6.72346900	-0.68460700	1.25008100
H	5.95144800	-0.81692000	2.00515900
C	6.55185700	-0.26349400	-2.33840100
H	7.29591900	-0.01434600	-3.10140200
H	6.46699100	-1.35063000	-2.28069900
H	5.59492800	0.14186600	-2.65497100
C	7.36602200	1.79541900	-1.09523100
H	6.63783500	2.38797900	-1.64572900
H	7.48670600	2.23545000	-0.10396500
H	8.32515800	1.87099700	-1.61996600
C	7.32276700	-2.05695800	0.93690300
H	6.57842000	-2.73443600	0.52387900
H	8.16308600	-1.98112600	0.23796900
H	7.70524700	-2.50447000	1.85928900
C	7.80967300	0.20811500	1.85963800
H	7.43701700	1.19787600	2.12030600
H	8.18184200	-0.26711300	2.77250100
H	8.66455300	0.32935600	1.18700100
H	2.25549500	-0.15956700	4.49199100
H	1.85683500	-0.88198900	2.92383100
C	-3.64462100	3.12726400	0.38739900
C	-3.72904600	2.02966800	1.27769100
C	-3.64448400	2.24357400	2.67328000

C	-3.50025800	3.54300000	3.15755600
C	-3.43297900	4.62886500	2.29038200
C	-3.50289400	4.41269200	0.91985600
N	-3.73902600	0.70245700	0.81584200
C	-3.68127600	1.06215600	3.62411400
C	-4.55780000	1.30839300	4.85297300
C	-3.71131800	2.96731200	-1.12278400
C	-2.46615800	3.52973200	-1.81933800
C	-4.68478800	0.04908500	0.10196900
N	-4.11070900	-0.87129100	-0.70211500
C	-4.60534700	-2.08163100	-1.20354100
C	-4.92211600	-3.18387400	-0.37253400
C	-5.44334400	-4.33635600	-0.97219200
C	-5.58812400	-4.44909400	-2.34881100
C	-5.12102600	-3.42209200	-3.16280000
C	-4.60044100	-2.25203100	-2.61291800
C	-4.66306000	-3.26072000	1.12606000
C	-5.75379000	-2.62950600	1.98744700
C	-3.91837700	-1.21842100	-3.49276000
C	-4.53688100	-1.06037700	-4.88126200
C	-3.28815400	-2.74541500	1.56211600
C	-2.43003500	-1.57314500	-3.60644800
C	-2.26129200	0.64869900	4.02328600
C	-4.95690200	3.64501600	-1.70841200
Mg	-2.20865000	-0.32228800	-0.10000800
H	-5.15082300	-3.53813800	-4.24178400
H	-5.71948200	-5.17278900	-0.33386800
H	-3.43703900	3.70947000	4.22913800
H	-3.44380400	5.26108000	0.24301500
H	-4.10682500	0.21992600	3.06761500
H	-3.76889200	1.89658500	-1.34893600
H	-3.98211600	-0.25665000	-2.97450900
H	-4.66745700	-4.33603300	1.34544700
H	-6.00725700	-5.35083400	-2.78530500
H	-1.88219100	-0.80458500	-4.16297600
H	-1.96461800	-1.66797800	-2.62121600
H	-2.30016000	-2.53075900	-4.12236000
H	-4.06386200	-0.22610800	-5.40931100
H	-4.38938300	-1.95447700	-5.49646600
H	-5.61170200	-0.86282600	-4.82799800
H	-5.57319100	-2.83367600	3.04876300
H	-5.76199100	-1.54950400	1.84604300
H	-6.74234700	-3.01702700	1.72474000
H	-3.02531400	-3.15740700	2.54239700
H	-2.50637000	-3.04279200	0.85710700
H	-3.28726100	-1.65521400	1.67669100
H	-1.75335900	1.46110500	4.55484100
H	-4.65002300	0.39077200	5.44319700
H	-4.13548500	2.07567700	5.51038000
H	-5.56357000	1.63288200	4.56682300
H	-2.50425400	3.32110900	-2.89408600
H	-2.40376100	4.61606800	-1.69719500
H	-1.54481100	3.10194700	-1.42255800
H	-5.00210400	3.49659300	-2.79290200
H	-5.87768400	3.25570300	-1.27112900
H	-4.93239100	4.72411200	-1.52244900
H	-3.32154300	5.63629700	2.68062800
N	-6.03351300	0.30783000	0.22622200
C	-7.05989700	0.07335300	-0.81184600
H	-7.83412400	0.80230200	-0.55230600
C	-6.53945000	1.00971300	1.43151200
H	-5.72696100	0.96878800	2.15319600

C	-6.60698900	0.42212200	-2.22253800
H	-7.48934500	0.57683000	-2.85137600
H	-6.00828400	1.33250900	-2.24356300
H	-6.03424800	-0.39194900	-2.65738100
C	-7.73085800	-1.30234000	-0.78689700
H	-7.08009200	-2.08024100	-1.18388100
H	-8.03300500	-1.58850200	0.22084300
H	-8.63053700	-1.26413300	-1.41082200
C	-6.89270400	2.47840800	1.19580400
H	-6.03247300	3.05307900	0.86049200
H	-7.70233900	2.58700500	0.46561100
H	-7.24138500	2.91952100	2.13448400
C	-7.74489900	0.31002400	2.06843100
H	-7.55965600	-0.74245400	2.28014800
H	-7.96906500	0.80866700	3.01617700
H	-8.64082100	0.38590900	1.44473600
H	-2.27737100	-0.23037200	4.67715200
H	-1.66820100	0.40745300	3.13760500

178

Int4

C	-0.18314500	1.26359900	-0.34928700
C	0.49689100	1.10803200	0.80900600
C	0.89916800	-0.22222400	1.36618700
C	0.27275800	-1.34622600	0.61427300
C	-0.42019800	-1.19256700	-0.53631000
C	-0.81009100	0.13940800	-1.11039100
H	0.83444100	2.00074200	1.33914500
H	0.79056100	-0.30181900	2.45301600
H	0.42356900	-2.35577100	0.99618000
H	-0.76524400	-2.08873700	-1.05170900
H	-0.61432900	0.20407700	-2.18989700
H	-0.33480600	2.27412400	-0.72752300
C	5.42838900	2.10163200	2.22344400
C	4.91852100	2.22340100	0.91032900
C	4.71091700	3.49832700	0.34263400
C	5.03696600	4.63222000	1.08854400
C	5.55329500	4.52183300	2.37495800
C	5.74037600	3.26193800	2.93465100
N	4.48321900	1.08669600	0.20613100
C	4.11615100	3.62258300	-1.04646600
C	4.81835700	4.67572100	-1.90528100
C	5.56657100	0.73672000	2.87172900
C	4.29942900	0.39991100	3.66866400
C	5.22603400	0.04306600	-0.23339200
N	4.51765600	-1.10416400	-0.17731300
C	4.56801800	-2.23171800	-1.00349100
C	4.22560800	-2.16966600	-2.37473300
C	4.35735500	-3.33255200	-3.14051700
C	4.73107400	-4.54478700	-2.57354500
C	4.88543600	-4.62706600	-1.19250600
C	4.77586000	-3.49230800	-0.39016500
C	3.62202300	-0.95393200	-3.06575300
C	4.65027900	0.04844200	-3.58871500
C	4.74263800	-3.60844100	1.12266400
C	5.66897500	-4.68263300	1.69041100
C	2.53522600	-0.22288900	-2.26354500
C	3.29299700	-3.84181100	1.57094800
C	2.60796500	3.88195800	-0.96471200
C	6.80839900	0.60132000	3.75387300
Mg	2.86310400	-0.14426800	0.55853000
H	5.08158200	-5.59075800	-0.73232000

H	4.13116800	-3.28376100	-4.20356300
H	4.88326100	5.61701500	0.65644900
H	6.13283800	3.17996200	3.94401700
H	4.24307600	2.64914700	-1.53371400
H	5.65801300	-0.00820900	2.07711800
H	5.04077800	-2.63870300	1.53336100
H	3.11496500	-1.37329100	-3.94399700
H	4.83821800	-5.43070500	-3.19251800
H	3.22038800	-3.87578800	2.66374900
H	2.63606600	-3.04292500	1.21299900
H	2.91067300	-4.78667500	1.16983100
H	5.67873200	-4.63359900	2.78412600
H	5.33810200	-5.69022700	1.41685500
H	6.69565100	-4.55786900	1.33332000
H	4.16220000	0.81982600	-4.19493400
H	5.15346000	0.54136700	-2.75714000
H	5.40868500	-0.44480800	-4.20435900
H	1.85450300	0.30382500	-2.93851800
H	1.90870700	-0.91303000	-1.68327000
H	2.97005800	0.55966100	-1.62802700
H	2.40311700	4.81713600	-0.43154800
H	4.43711500	4.64392900	-2.93119100
H	4.64965400	5.68986900	-1.52778300
H	5.90005100	4.50873700	-1.93792100
H	4.35028100	-0.61413600	4.08022400
H	4.16840600	1.10376400	4.49780000
H	3.39505400	0.46452200	3.05066600
H	6.92054300	-0.43475700	4.08992200
H	7.71591200	0.88462700	3.21159300
H	6.74524100	1.22722600	4.65013700
H	5.80354800	5.41368200	2.94232200
N	6.52194600	0.17290600	-0.68118800
C	7.54860500	-0.89082000	-0.67738900
H	8.48147200	-0.32515200	-0.74698700
C	7.05211000	1.50862500	-1.02898400
H	6.18646800	2.16315800	-1.08747000
C	7.62034600	-1.63714400	0.64593600
H	8.47207700	-2.32433700	0.63208600
H	7.75251200	-0.94083400	1.47788100
H	6.72142500	-2.22176300	0.81967700
C	7.54344800	-1.82952100	-1.88622200
H	6.80943100	-2.62820600	-1.79627900
H	7.34140800	-1.28026600	-2.80758600
H	8.53312400	-2.29101700	-1.97537800
C	8.00642800	2.07161500	0.02528900
H	7.55148600	2.06889100	1.01503200
H	8.94515000	1.50810100	0.06360500
H	8.25581000	3.10719800	-0.22479800
C	7.72572400	1.52904500	-2.40395200
H	7.07091900	1.15134400	-3.18965300
H	7.98922800	2.56243500	-2.64919300
H	8.65230700	0.94680700	-2.41964900
H	2.17130500	3.95719600	-1.96698600
H	2.09913900	3.07014800	-0.43809200
C	-3.97797400	-1.94041800	2.21789300
C	-4.28633100	-2.16977400	0.85691900
C	-4.34120600	-3.48367700	0.34779000
C	-4.11245300	-4.55356000	1.21485500
C	-3.82645100	-4.33849000	2.55882100
C	-3.75583500	-3.03819700	3.05003400
N	-4.40667000	-1.09310700	-0.04078600
C	-4.61111500	-3.69865900	-1.12859600

C	-5.40785500	-4.96798600	-1.42688400
C	-3.82446000	-0.51963100	2.72605800
C	-2.39241400	-0.02370000	2.49191800
C	-5.30386700	-0.07758000	0.00659500
N	-4.73567900	1.07785100	-0.39461400
C	-5.30446900	2.19756600	-1.01097300
C	-5.84236000	2.14491500	-2.31805700
C	-6.43449900	3.30199700	-2.83553700
C	-6.43788600	4.50034900	-2.13273300
C	-5.75186200	4.57885300	-0.92380400
C	-5.15235000	3.45097800	-0.36628200
C	-5.75062700	0.94406900	-3.24937100
C	-6.85974200	-0.08837100	-3.05163200
C	-4.23040300	3.56762800	0.83383900
C	-4.64555500	4.62824500	1.85145600
C	-4.38311200	0.24567900	-3.26895700
C	-2.80272800	3.82128400	0.33103500
C	-3.29852300	-3.66866100	-1.91970700
C	-4.22365300	-0.33390600	4.18894000
Mg	-2.90323500	0.17329300	-0.66488700
H	-5.67116200	5.53449800	-0.41489700
H	-6.88240900	3.25883100	-3.82601600
H	-4.15914200	-5.56986100	0.83528100
H	-3.51988200	-2.87589800	4.09733600
H	-5.20021200	-2.83957700	-1.47265600
H	-4.48917100	0.11711700	2.13832800
H	-4.21565200	2.59387000	1.33352600
H	-5.88768300	1.36969200	-4.25160700
H	-6.91756300	5.38047100	-2.55069800
H	-2.08180900	3.80556500	1.15491500
H	-2.49928800	3.05754200	-0.39178900
H	-2.73687700	4.79211200	-0.17213800
H	-3.99638800	4.57983700	2.73160400
H	-4.55743900	5.64037900	1.44235400
H	-5.67926000	4.48764300	2.18134700
H	-6.80652900	-0.86476500	-3.82312200
H	-6.75693400	-0.56797200	-2.07833800
H	-7.84822000	0.37783800	-3.10074200
H	-4.24639800	-0.28782000	-4.21529500
H	-3.55891100	0.96364700	-3.18074800
H	-4.31532200	-0.51427400	-2.48054500
H	-2.63046700	-4.47328800	-1.59412700
H	-5.68453300	-5.00098600	-2.48568600
H	-4.82868200	-5.87354600	-1.21666500
H	-6.32590000	-5.01167500	-0.83200100
H	-2.31284000	1.06104500	2.61420100
H	-1.69907100	-0.49695000	3.19444100
H	-1.99957800	-0.29587100	1.50500000
H	-4.19657300	0.72839300	4.45311900
H	-5.23500200	-0.70744600	4.37947500
H	-3.53982600	-0.85293000	4.86865100
H	-3.65247000	-5.18145500	3.22138600
N	-6.61289400	-0.25277300	0.39964400
C	-7.50477900	0.78416700	0.96412000
H	-8.28080000	0.19039500	1.45343600
C	-7.17498300	-1.61956900	0.48108000
H	-6.46155100	-2.26286100	-0.02721500
C	-6.85144700	1.59405600	2.07346500
H	-7.59276600	2.26560400	2.51768600
H	-6.46568000	0.94028300	2.85934000
H	-6.03676700	2.20332500	1.69250600
C	-8.24169500	1.66344900	-0.04848600

H	-7.63246600	2.48654500	-0.41706500
H	-8.58112400	1.07591100	-0.90332500
H	-9.12365100	2.09193400	0.44024500
C	-7.32936300	-2.12209900	1.91747600
H	-6.39726500	-2.02774100	2.47340600
H	-8.11988900	-1.58561500	2.45361300
H	-7.60369000	-3.18111900	1.90418600
C	-8.50718300	-1.74695300	-0.26254000
H	-8.43101000	-1.43046500	-1.30336800
H	-8.81617900	-2.79663500	-0.24915600
H	-9.30506300	-1.16892900	0.21409600
H	-3.48322400	-3.78697200	-2.99331200
H	-2.77700400	-2.71993300	-1.76642600

178

TS1

C	1.11812900	-1.77127400	-3.29226500
C	-0.16834700	-1.61203000	-2.85484800
C	-0.59084800	-0.28221100	-2.40138900
C	-0.02838500	0.77356100	-3.24541600
C	1.25673800	0.62704500	-3.69755400
C	1.95006400	-0.61137100	-3.45855600
H	-0.81783100	-2.47373800	-2.72150500
H	0.19276700	-0.13650900	-1.16791800
H	-0.56525100	1.70715300	-3.39672400
H	1.76758100	1.43013400	-4.22597800
H	2.95983500	-0.73881100	-3.84039900
H	1.52750200	-2.75348800	-3.52056500
C	-5.69117500	-0.20583000	-2.48223000
C	-5.11526300	0.78905500	-1.66143600
C	-5.42551200	2.15101900	-1.86229200
C	-6.35373900	2.49506500	-2.84541000
C	-6.95359100	1.51895600	-3.63506200
C	-6.61112000	0.18229100	-3.45876300
N	-4.11107500	0.46705500	-0.73231500
C	-4.69615600	3.19885500	-1.04552000
C	-5.52363500	4.45087900	-0.76105400
C	-5.25141400	-1.65206700	-2.35448900
C	-4.06864400	-1.92704200	-3.29303700
C	-4.19439800	-0.33688800	0.35350700
N	-3.02538400	-0.98271300	0.56207700
C	-2.42788600	-1.37272300	1.76585000
C	-2.04064000	-0.42621600	2.74412000
C	-1.51811300	-0.90071300	3.95114000
C	-1.27996400	-2.25315900	4.16642800
C	-1.48871000	-3.14906600	3.12196800
C	-2.03644600	-2.72688900	1.91060600
C	-2.03974300	1.08203000	2.54607400
C	-3.36272600	1.77708100	2.85989600
C	-2.09895300	-3.67402700	0.72672600
C	-2.53912700	-5.09320000	1.08788300
C	-1.50574900	1.55994800	1.18937200
C	-0.73428000	-3.69559700	0.02469400
C	-3.36899600	3.55971700	-1.72622100
C	-6.37413200	-2.66116900	-2.59638100
Mg	-2.16516600	-0.08342300	-1.06134000
H	-1.21670100	-4.19274200	3.24892500
H	-1.26249600	-0.17921700	4.72400500
H	-6.60946800	3.53895000	-2.99993200
H	-7.06451400	-0.57226500	-4.09454000
H	-4.44312200	2.73517400	-0.08433000
H	-4.89859700	-1.80802000	-1.33209300

H	-2.81659000	-3.25624100	0.01330800
H	-1.31208300	1.44420400	3.27894800
H	-0.87824900	-2.59664600	5.11510900
H	-0.76393200	-4.32416300	-0.87174200
H	-0.41980100	-2.69366400	-0.28071600
H	0.03589700	-4.09446900	0.69331800
H	-2.67493200	-5.68957000	0.17980000
H	-1.78850400	-5.60539000	1.69933500
H	-3.48124000	-5.09513600	1.64312900
H	-3.22996500	2.86483800	2.85596800
H	-4.11519500	1.52695400	2.11251800
H	-3.74253000	1.48180400	3.84275000
H	-1.06851000	2.55538800	1.29618400
H	-0.69721400	0.92102500	0.81414400
H	-2.31742200	1.65687300	0.45494400
H	-3.54974500	4.02704600	-2.70011800
H	-4.98732800	5.10923800	-0.07002700
H	-5.71877700	5.02834400	-1.67105500
H	-6.48853800	4.19669300	-0.31081800
H	-3.67572700	-2.93874600	-3.14418700
H	-4.37737700	-1.82996300	-4.33934800
H	-3.24319800	-1.22036300	-3.14681300
H	-6.02413300	-3.67404300	-2.37194900
H	-7.24368700	-2.45289100	-1.96496000
H	-6.70867400	-2.65906800	-3.63897200
H	-7.67571100	1.80060200	-4.39601300
N	-5.32813300	-0.44993900	1.12426600
C	-5.69373800	-1.62310000	1.94648000
H	-6.76924400	-1.49211600	2.09032500
C	-6.40296900	0.56046300	1.01259000
H	-5.97182600	1.39298400	0.45953700
C	-5.53558200	-2.93477600	1.19229700
H	-5.86993600	-3.76419600	1.82296900
H	-6.13673400	-2.93219200	0.27967000
H	-4.49758500	-3.11226000	0.92274000
C	-5.09265300	-1.66921000	3.35356900
H	-4.08545800	-2.08164900	3.36864900
H	-5.06193000	-0.67334900	3.79936000
H	-5.72498900	-2.30482200	3.98317400
C	-7.62481900	0.05734800	0.24234800
H	-7.34618300	-0.33495200	-0.73443700
H	-8.16130000	-0.71940600	0.79811500
H	-8.32125400	0.88600400	0.08331800
C	-6.83953900	1.09616000	2.37916400
H	-6.00062000	1.47785400	2.96103800
H	-7.54768700	1.91584700	2.22417600
H	-7.35221000	0.33462300	2.97499000
H	-2.78631600	4.25506500	-1.11239600
H	-2.75716800	2.66861100	-1.90034100
C	5.83604800	-0.33474600	-2.24474100
C	5.09077600	-1.10743000	-1.32041200
C	5.24327000	-2.51155400	-1.28349600
C	6.09947700	-3.12870000	-2.19624400
C	6.80592900	-2.38162800	-3.13181400
C	6.67777700	-0.99594500	-3.14131300
N	4.06788300	-0.55651600	-0.53664500
C	4.45563500	-3.31940400	-0.27066400
C	5.24867700	-4.48539400	0.32064500
C	5.74072900	1.18164800	-2.24181900
C	4.55527100	1.70459900	-3.05366200
C	4.07956800	0.44318300	0.36735300
N	2.89417900	1.08566300	0.36530200

C	2.23834700	1.75252600	1.40198300
C	1.79187800	1.09677000	2.57565700
C	1.21166500	1.87522800	3.58359700
C	0.98055500	3.23622600	3.42606600
C	1.25924900	3.82899300	2.19697700
C	1.85913700	3.10081700	1.17097400
C	1.80770400	-0.40615300	2.81492200
C	3.11233000	-0.94361400	3.39543700
C	2.00094400	3.68963300	-0.22007000
C	2.31313800	5.18469900	-0.24261300
C	1.38380100	-1.26594000	1.62369000
C	0.72903800	3.38413900	-1.02259300
C	3.13106000	-3.80420600	-0.86855200
C	7.02672400	1.87356100	-2.69833000
Mg	2.12158100	-0.12710800	-1.16043600
H	1.00286300	4.87148400	2.03400600
H	0.91424800	1.38331200	4.50721100
H	6.21553800	-4.20848400	-2.17505400
H	7.25114500	-0.41595300	-3.85778800
H	4.20354700	-2.63536700	0.54825700
H	5.57019200	1.48054500	-1.20591400
H	2.81727100	3.15696200	-0.71760300
H	1.03284300	-0.56317400	3.57261900
H	0.53822100	3.81633000	4.23067700
H	0.82994600	3.71334100	-2.06183800
H	0.51596300	2.31038400	-1.03355700
H	-0.13668300	3.89196200	-0.58351700
H	2.51109100	5.51260300	-1.26821700
H	1.47331300	5.78207500	0.12880400
H	3.19000500	5.42146800	0.36743800
H	2.99720200	-1.99595900	3.67866100
H	3.90650600	-0.87789700	2.65271000
H	3.42031000	-0.38138200	4.28232400
H	1.05046800	-2.24326500	1.98164200
H	0.54488800	-0.82527200	1.07863600
H	2.21901400	-1.44420100	0.93554400
H	3.31193000	-4.48123100	-1.71098600
H	4.68410200	-4.95177200	1.13489400
H	5.44757500	-5.26521100	-0.42235100
H	6.21123200	-4.15004100	0.71980600
H	4.51842100	2.79898900	-3.02077100
H	4.62519100	1.39238900	-4.10117800
H	3.60776800	1.33281200	-2.66208300
H	6.95566400	2.95033400	-2.51174400
H	7.90574300	1.49349700	-2.16926100
H	7.19849300	1.74539600	-3.77240600
H	7.46423300	-2.87342400	-3.84218600
N	5.16385000	0.72245200	1.17935700
C	5.47994300	2.03670700	1.78175200
H	6.54671100	1.94964500	2.00497300
C	6.22725900	-0.29426500	1.35944100
H	5.81421600	-1.22645300	0.97297300
C	5.35739000	3.19703100	0.80308100
H	5.60587100	4.13057600	1.31720000
H	6.04353500	3.08096200	-0.03927600
H	4.34459300	3.28559400	0.41867000
C	4.80279100	2.34712500	3.11946800
H	3.79630300	2.74361600	2.99794400
H	4.74865600	1.45932700	3.75076600
H	5.39925900	3.10146400	3.64452100
C	7.50999100	0.01797300	0.58681800
H	7.33417200	0.06485000	-0.48509000

H	7.95903400	0.96114900	0.91641200
H	8.24401900	-0.77312200	0.76791700
C	6.58071500	-0.51911000	2.83409700
H	5.70865900	-0.74231500	3.44721200
H	7.26812000	-1.36769200	2.90172400
H	7.09245000	0.34545000	3.26849800
H	2.53285000	-4.33735500	-0.12183900
H	2.53247000	-2.96999300	-1.24549600

178

Int5

C	1.38778000	0.70215800	-2.61647900
C	0.15321300	0.30876200	-2.08808900
C	-0.77617500	1.22766400	-1.55967400
C	-0.38505700	2.58189900	-1.60633700
C	0.85043300	2.99780000	-2.10609000
C	1.75188300	2.05607200	-2.61132200
H	-0.06067200	-0.75758700	-2.09363200
H	1.14478700	1.64232100	1.00878600
H	-1.04596400	3.35165000	-1.21212500
H	1.11918200	4.05087500	-2.09587500
H	2.71100100	2.37035000	-3.01302000
H	2.05268600	-0.04955900	-3.02570400
C	-5.91531900	1.43419900	-2.13358800
C	-5.37224800	1.93496000	-0.92819600
C	-5.61430500	3.26883300	-0.53574500
C	-6.43389100	4.06969300	-1.33227500
C	-6.99821300	3.57547700	-2.50372900
C	-6.72919800	2.26962000	-2.90119600
N	-4.47916900	1.15976000	-0.16726600
C	-4.94407100	3.80590200	0.71327000
C	-5.76796100	4.85810000	1.45329000
C	-5.54700100	0.04313900	-2.61492400
C	-4.25859700	0.09685700	-3.44677300
C	-4.72701400	-0.04787400	0.40581500
N	-3.64632000	-0.84541100	0.30934700
C	-3.17771300	-1.88407300	1.12192200
C	-2.68175200	-1.64588300	2.42413700
C	-2.22169900	-2.73981700	3.16463200
C	-2.17050900	-4.01928200	2.62682400
C	-2.51574300	-4.20988500	1.29162500
C	-2.99305100	-3.15315900	0.51728800
C	-2.53591800	-0.27297200	3.06719600
C	-3.80827000	0.19442100	3.77401300
C	-3.18974800	-3.30860800	-0.98135400
C	-3.65259000	-4.69919200	-1.41177500
C	-2.00081600	0.84829300	2.16312700
C	-1.89805200	-2.91649400	-1.70692500
C	-3.54725800	4.33663700	0.37007400
C	-6.65853400	-0.65816400	-3.39500800
Mg	-2.53005300	0.64102900	-0.55939800
H	-2.39721300	-5.19136800	0.84331100
H	-1.86297200	-2.56885700	4.17706300
H	-6.63571300	5.09380300	-1.03334200
H	-7.15676500	1.89580500	-3.82660500
H	-4.79679700	2.95340300	1.38796600
H	-5.33734900	-0.57487800	-1.73894100
H	-3.94499600	-2.57996700	-1.29020800
H	-1.77228000	-0.41707600	3.84189100
H	-1.81046000	-4.85087400	3.22489000
H	-2.03311400	-2.94167200	-2.79366600
H	-1.59313700	-1.90403100	-1.42923700

H	-1.06991900	-3.58256100	-1.44757200
H	-3.88722100	-4.70315100	-2.48097800
H	-2.87496400	-5.45346500	-1.25093000
H	-4.54626600	-5.01545100	-0.86502400
H	-3.63419200	1.13878000	4.30175600
H	-4.60185000	0.35068400	3.04249900
H	-4.15494100	-0.54781500	4.49888900
H	-1.55335900	1.63806200	2.77335700
H	-1.19091800	0.51246200	1.50299300
H	-2.81508200	1.33319800	1.60730200
H	-3.61291500	5.17879900	-0.32708800
H	-5.29097500	5.11025600	2.40577900
H	-5.85478900	5.78743500	0.88025200
H	-6.77996300	4.49694200	1.66283400
H	-3.90751400	-0.90948700	-3.69856000
H	-4.42430400	0.64867500	-4.37822600
H	-3.44566400	0.61639500	-2.92513400
H	-6.37581200	-1.69561300	-3.60133500
H	-7.59747900	-0.66588800	-2.83232200
H	-6.84973300	-0.17739300	-4.36002400
H	-7.63742500	4.20946700	-3.11136300
N	-5.92343600	-0.34608500	1.01731800
C	-6.48084800	-1.69669400	1.25252100
H	-7.54041200	-1.49076900	1.42340600
C	-6.87774200	0.74450300	1.32209000
H	-6.32387200	1.66827500	1.18246700
C	-6.43248800	-2.59447700	0.02594100
H	-7.04573600	-3.48295200	0.20600900
H	-6.82486100	-2.07993000	-0.85444700
H	-5.42045400	-2.92990800	-0.18007600
C	-5.99064800	-2.41152400	2.51193100
H	-4.99290800	-2.83096700	2.39456500
H	-5.98190400	-1.73610600	3.36911400
H	-6.67827300	-3.23424600	2.73586600
C	-8.08218200	0.76536900	0.37980400
H	-7.76832700	0.76492000	-0.66366600
H	-8.74933400	-0.08647100	0.55117400
H	-8.66289100	1.67624100	0.55271600
C	-7.34748800	0.72150900	2.77829600
H	-6.51359800	0.75952700	3.48070100
H	-7.97742900	1.59858400	2.95525500
H	-7.95121700	-0.16185900	3.00830000
H	-3.01662900	4.67034500	1.26821400
H	-2.94520900	3.55791600	-0.10720100
C	5.13810400	1.94414700	2.36629800
C	5.17390200	2.02617500	0.95532000
C	5.84706200	3.10208400	0.33156600
C	6.51144000	4.04371200	1.11623000
C	6.50630300	3.95082100	2.50423300
C	5.81211700	2.91369100	3.11487400
N	4.43748500	1.14576000	0.14295100
C	5.78770300	3.24964200	-1.17542700
C	7.12952700	3.62606200	-1.80397700
C	4.34783600	0.87073200	3.09071000
C	3.08333100	1.46425800	3.72434300
C	4.61702700	-0.18538300	-0.01336400
N	3.45551600	-0.78182600	-0.32467700
C	3.11871600	-1.96807700	-0.98048700
C	3.42138500	-2.26279600	-2.33685000
C	2.85387500	-3.41854600	-2.89745500
C	2.02808100	-4.27542700	-2.18860100
C	1.71349200	-3.96508600	-0.87023900

C	2.22405000	-2.82098800	-0.26615600
C	4.24278800	-1.44425800	-3.34507500
C	5.43336300	-2.24918900	-3.89085600
C	1.74232600	-2.43755600	1.12225500
C	1.62252100	-3.62004400	2.08644600
C	4.74771100	-0.05558300	-2.96370500
C	0.39715500	-1.71307600	1.03579100
C	4.69047400	4.25114400	-1.55547900
C	5.18486900	0.13932900	4.14494600
Mg	2.36009100	0.98034800	-0.01972200
H	1.04342500	-4.60897500	-0.30797800
H	3.05677600	-3.63482400	-3.94352900
H	7.03432100	4.86612600	0.63576900
H	5.78528500	2.85648900	4.19978400
H	5.48394100	2.27658400	-1.57401500
H	4.02536600	0.13182900	2.35056000
H	2.46646800	-1.73000600	1.53497200
H	3.55598300	-1.30209700	-4.19380100
H	1.61599000	-5.16049500	-2.66445000
H	0.11334800	-1.32253100	2.01599300
H	0.42951800	-0.87138000	0.33759300
H	-0.38881500	-2.39725000	0.70630600
H	1.39770200	-3.25527500	3.09397600
H	0.80320800	-4.28882500	1.80388400
H	2.54429100	-4.20760800	2.13128800
H	5.90402500	-1.70934700	-4.71969700
H	6.18542000	-2.39281500	-3.11254600
H	5.14155100	-3.23740100	-4.25368400
H	5.11562800	0.44697300	-3.86487400
H	3.99123400	0.57742500	-2.50704900
H	5.57818200	-0.11899900	-2.26315600
H	4.92409200	5.24896100	-1.16772100
H	7.05459400	3.61676100	-2.89656400
H	7.45403100	4.62977200	-1.50904800
H	7.91614000	2.92307800	-1.51095800
H	2.50641000	0.68203300	4.23149600
H	3.34440800	2.22880900	4.46473300
H	2.43927500	1.91889500	2.96641100
H	4.61004500	-0.68204100	4.58656200
H	6.10208100	-0.27606600	3.71751800
H	5.47302500	0.80969700	4.96133800
H	7.02747800	4.69017000	3.10574800
N	5.84583400	-0.80660100	0.11762300
C	6.06356900	-2.18728800	0.59619600
H	7.05957200	-2.13818500	1.04893000
C	7.07798600	-0.01288000	-0.07385400
H	6.74638700	0.94040700	-0.48030900
C	5.10927900	-2.60905200	1.70380000
H	5.51435400	-3.49280500	2.20646900
H	4.97924100	-1.82307500	2.44727100
H	4.13883000	-2.87968000	1.29594400
C	6.12684200	-3.27145300	-0.48079000
H	5.15753800	-3.42907400	-0.95334800
H	6.85680300	-3.03376300	-1.25487400
H	6.43342500	-4.21393500	-0.01398600
C	7.85106500	0.27061700	1.21476600
H	7.21279900	0.72272100	1.97175800
H	8.30689700	-0.63669700	1.62602000
H	8.66222700	0.97299100	1.00024700
C	8.01817300	-0.63978500	-1.10649700
H	7.51020500	-0.85432300	-2.04889700
H	8.83396000	0.05982300	-1.31376400

H	8.47091700	-1.56524100	-0.73752500
H	4.58343400	4.32555900	-2.64416200
H	3.72758300	3.94834600	-1.13298900

178

4

Mg	-1.31538200	0.21839600	0.43550700
Mg	1.33762200	-0.16984100	0.44806600
N	3.08434000	-0.93863200	-0.29493800
N	-3.09932900	1.00222600	-0.22057000
N	-2.75951700	-1.17629500	0.10644700
N	-4.79192600	-0.55208200	-0.99065700
N	2.83241900	1.19171200	0.29403500
N	4.84111900	0.63369100	-0.88183900
C	-3.59967300	-0.23938400	-0.37561900
C	3.38777000	-2.07256100	-1.06674600
C	3.63586400	0.29096600	-0.30838100
C	-3.91064100	-2.35327900	1.93643600
C	-3.41578500	2.17637800	-0.92626400
C	4.00807300	-3.15913900	-0.40136900
C	-3.06366800	-2.35180800	0.79889400
C	-4.07858900	3.20641400	-0.21861300
C	-4.62126100	-1.08717200	2.38552900
H	-4.87046600	-0.50936200	1.49249300
C	2.95717500	-2.20838500	-2.40282300
C	-0.00745000	-0.16910600	2.24113300
C	3.18269100	2.36324400	0.96802200
C	-2.35534400	-3.52120500	0.44323500
C	4.13542100	2.37269800	2.01620000
C	-3.70715700	-0.21520600	3.25207400
H	-3.40219400	-0.75162100	4.15557200
H	-4.22014800	0.70504200	3.55058300
H	-2.79397900	0.06701500	2.72367700
C	4.38577000	-3.00896300	1.06027600
H	4.60703500	-1.94835700	1.22602700
C	-4.03497700	-3.53064200	2.67483300
H	-4.68018200	-3.54394400	3.54766900
C	-4.92131400	-1.89670700	-1.58771300
H	-3.90167100	-2.28442300	-1.56356400
C	-5.88865700	0.43300400	-0.97175800
H	-5.54562600	1.19375800	-0.27147600
C	4.80522100	1.08363800	2.45709900
H	5.00251500	0.48993400	1.56276000
C	5.86972700	-0.39873800	-1.09107600
H	5.41611700	-1.31366300	-0.72051100
C	-4.44822500	2.98897400	1.23598300
H	-4.57958100	1.91074900	1.37621900
C	-2.95404300	2.40027800	-2.24086100
C	3.20318600	-3.41898000	-3.06233300
H	2.87431300	-3.53520400	-4.09164300
C	2.44812100	3.53419900	0.66360100
C	-5.94066500	-1.35252800	3.11253400
H	-6.58682300	-2.02998000	2.54496400
H	-6.47904300	-0.41107800	3.26279600
H	-5.78062200	-1.79269300	4.10251700
C	0.32142000	0.87709400	3.13663000
H	0.66125000	1.83703700	2.74946000
C	2.14866600	-1.13620900	-3.11168400
H	2.14349300	-0.24055100	-2.48151700
C	4.36188100	3.56834900	2.69983400
H	5.09279500	3.59183300	3.50197500
C	4.22862500	-4.34561500	-1.09621900

H	4.71015000	-5.17974800	-0.59512700
C	-2.50673700	-4.67333500	1.21759900
H	-1.95724300	-5.57120700	0.94639200
C	-3.34264900	-4.68765700	2.32673200
H	-3.45401900	-5.59050900	2.92022500
C	-4.32672300	4.41986500	-0.85715300
H	-4.84475300	5.21094100	-0.32335800
C	1.37540000	3.49649500	-0.40772900
H	1.56673200	2.60142700	-1.00944000
C	3.83766500	-4.47730800	-2.42690900
H	4.01734500	-5.40662600	-2.95988200
C	3.65666800	4.72883100	2.39249500
H	3.84956000	5.64600300	2.94158500
C	-3.22284900	3.63539500	-2.84159700
H	-2.87745700	3.81547100	-3.85627400
C	-0.40297600	-1.38984400	2.84076800
H	-0.69434100	-2.23359100	2.21898700
C	-2.13722800	1.37430600	-3.00628600
H	-2.06682200	0.46595300	-2.39886600
C	-3.91238700	4.63521200	-2.16895300
H	-4.11382900	5.58436400	-2.65727900
C	2.69677800	4.70088200	1.38775300
H	2.13381400	5.60122800	1.15721900
C	-6.16758700	1.14843600	-2.29486500
H	-5.24830700	1.51289500	-2.75061700
H	-6.80107500	2.01889600	-2.09660100
H	-6.69367500	0.51111500	-3.00888900
C	3.85945000	0.26444000	3.34271700
H	3.60877600	0.81406900	4.25603000
H	4.32107800	-0.68715700	3.62787600
H	2.92228900	0.04220900	2.82810500
C	-1.39831600	-3.51477600	-0.73359800
H	-1.55662700	-2.56840100	-1.26138500
C	6.14761900	1.29185300	3.15642200
H	6.82133300	1.91546800	2.55997000
H	6.63539600	0.32598800	3.32490100
H	6.02841700	1.76533200	4.13686200
C	-5.33756000	-1.84854100	-3.05670200
H	-6.38802700	-1.57057800	-3.17817200
H	-5.21444400	-2.84570700	-3.49145400
H	-4.72114100	-1.14960500	-3.62502700
C	7.13306100	-0.20036400	-0.24422800
H	7.79812100	0.57040900	-0.63665000
H	7.69336200	-1.14120500	-0.23122600
H	6.88266300	0.05432800	0.78680300
C	4.94712700	1.99546000	-1.44431300
H	4.31537500	2.62400900	-0.81982700
C	3.19011500	-3.35494300	1.95627200
H	2.32858800	-2.72352900	1.72459200
H	3.43594800	-3.20810700	3.01336400
H	2.88963900	-4.39851000	1.81294800
C	2.72648800	-0.75512700	-4.47775700
H	3.76932100	-0.43875300	-4.40656800
H	2.15365500	0.07084900	-4.91256000
H	2.67758800	-1.59331100	-5.18086000
C	0.25273200	0.72570700	4.52114600
H	0.51440700	1.55491600	5.17334400
C	6.21625600	-0.66330400	-2.55257900
H	5.32098400	-0.92183300	-3.11963600
H	6.90284300	-1.51367200	-2.60922600
H	6.70815800	0.19006300	-3.02728300
C	-0.71217400	1.89218300	-3.23693400

H	-0.72601200	2.85644300	-3.75589700
H	-0.13699100	1.19106400	-3.84840500
H	-0.18042800	2.01313800	-2.29192300
C	5.62174200	-3.81244500	1.46137200
H	5.43570300	-4.89175400	1.43835200
H	5.91867100	-3.55863100	2.48411500
H	6.46727200	-3.60257000	0.79843600
C	0.69424200	-1.59943400	-3.26997400
H	0.64834900	-2.56620500	-3.78281800
H	0.11719800	-0.87883400	-3.85646800
H	0.20507900	-1.69617900	-2.30012300
C	-0.46845300	-1.55758800	4.22315600
H	-0.78545500	-2.51048800	4.63831800
C	-0.00971300	3.33855200	0.23145800
H	-0.03770000	2.44324100	0.86497300
H	-0.80265000	3.26581000	-0.52005200
H	-0.24162100	4.18681900	0.88400200
C	-7.18588500	-0.13082700	-0.39103700
H	-7.68877700	-0.80442600	-1.08975400
H	-7.87049000	0.69957800	-0.19136900
H	-7.01369900	-0.66513500	0.54507900
C	-2.78643000	0.99662300	-4.34147600
H	-3.81833700	0.66426700	-4.20610000
H	-2.22642400	0.18784800	-4.82293400
H	-2.80071700	1.84601700	-5.03295700
C	-5.79889500	-2.89494800	-0.82381900
H	-5.62871900	-2.84406000	0.25086500
H	-5.53846500	-3.90815000	-1.14604300
H	-6.86286400	-2.74942500	-1.02429400
C	-3.28067700	3.40490000	2.13894600
H	-2.37746100	2.84092400	1.89071800
H	-3.51318200	3.22029300	3.19325800
H	-3.05650200	4.47008200	2.01589500
C	6.33755800	2.60850200	-1.34836000
H	7.07168300	2.11225600	-1.98844700
H	6.69821400	2.59833400	-0.31721500
H	6.27598200	3.65197700	-1.67152300
C	-1.67011000	-4.65460900	-1.71783400
H	-1.48234100	-5.63515100	-1.26723300
H	-1.01829900	-4.56402000	-2.59299500
H	-2.70990700	-4.63986500	-2.06004700
C	-5.74559000	3.67990700	1.65221000
H	-6.02190200	3.38145400	2.66875500
H	-6.57046800	3.41559400	0.98244500
H	-5.64947900	4.77098000	1.65156100
C	0.05799700	-3.54405500	-0.25464300
H	0.28423300	-2.65890700	0.35129100
H	0.76440700	-3.56883300	-1.08899000
H	0.25091300	-4.41570300	0.37933300
C	1.40128000	4.70767300	-1.34030400
H	1.14925800	5.63531500	-0.81532300
H	0.66890900	4.57920100	-2.14358300
H	2.38976100	4.83552800	-1.79207300
C	-0.14279000	-0.49572900	5.06602600
H	-0.19707400	-0.61871800	6.14455200
C	4.36966400	2.07437500	-2.85825500
H	4.32397100	3.11990500	-3.18141700
H	3.35237900	1.67494000	-2.87326500
H	4.96798600	1.52215900	-3.58646900
H	0.02875400	0.13228300	-0.80920000

84

LMgH

Mg	-0.00000300	-0.00152100	-2.60825000
N	1.11584200	-0.11499800	-0.88119000
N	-1.11596000	0.11512200	-0.88136500
N	-0.00024900	0.00065000	1.24565900
C	-2.38374100	-0.33903400	-0.46752300
C	-2.61196000	-1.71525200	-0.23328500
C	2.38383000	0.33878400	-0.46762600
C	-3.89311300	-2.12678300	0.13782600
H	-4.08346100	-3.17761800	0.33098400
C	2.61272800	1.71501700	-0.23418400
C	-4.70964500	0.13109500	-0.01178900
H	-5.52993000	0.83887800	0.06611500
C	-0.00007700	0.00031100	-0.13156600
C	-3.44186500	0.58643200	-0.37933600
C	-1.01646200	0.81039700	1.94395600
H	-1.53291000	1.32378100	1.13377500
C	-1.50053400	-2.72279900	-0.46516400
H	-0.55770900	-2.27267700	-0.13894900
C	-3.20185200	2.04421500	-0.71668300
H	-2.11873700	2.20509100	-0.66849100
C	1.50178200	2.72299400	-0.46638800
H	0.55873200	2.27339400	-0.14005300
C	3.44148700	-0.58716800	-0.37895900
C	1.01608100	-0.80828500	1.94469000
H	1.53253300	-1.32239000	1.13497100
C	-1.37410900	-3.02686000	-1.96460700
C	3.20062700	-2.04501200	-0.71542800
H	2.11747400	-2.20541000	-0.66640500
C	-4.93621300	-1.21271800	0.25938300
H	-5.92524500	-1.55191100	0.55340500
C	3.89413200	2.12613400	0.13653200
H	4.08504000	3.17698300	0.32907700
C	-0.39788000	1.90292600	2.81780800
H	-0.00178700	1.50626200	3.75541000
H	-1.17220200	2.63311000	3.07428300
H	0.40586700	2.42392400	2.29515000
C	-1.66744000	-4.02787300	0.31281800
H	-1.83895700	-3.84599700	1.37793800
H	-0.76753800	-4.64269300	0.21269200
H	-2.50785200	-4.61989700	-0.06471000
C	4.70952800	-0.13225400	-0.01178600
H	5.52946100	-0.84040200	0.06648200
C	3.86806400	-3.00889200	0.26649200
H	3.57743300	-4.04087300	0.04412900
H	3.57939300	-2.78857800	1.29937100
H	4.96051600	-2.96015800	0.20880900
C	2.07641600	-0.03239300	2.72911400
H	2.44642900	0.82625400	2.16905100
H	2.92683100	-0.69469600	2.91831300
H	1.70402000	0.30584400	3.69918400
C	4.93679000	1.21162300	0.25852400
H	5.92602000	1.55051700	0.55224200
C	-3.63336200	2.32820400	-2.16052100
H	-3.10965000	1.67141300	-2.86230800
H	-3.41946200	3.36638200	-2.43771700
H	-4.70828600	2.15662100	-2.28457600
C	-3.86905800	3.00825600	0.26524500
H	-4.96152500	2.95891900	0.20841000
H	-3.57913400	4.04027300	0.04213900
H	-3.57951300	2.78863200	1.29802800
C	1.37553400	3.02660800	-1.96591400

H	2.25693000	3.57144900	-2.31997400
H	0.48773900	3.63150200	-2.17784100
H	1.32264800	2.11452900	-2.57178700
C	0.39747600	-1.90011500	2.81944100
H	0.00139300	-1.50270000	3.75673500
H	1.17177700	-2.63013100	3.07645700
H	-0.40628700	-2.42141500	2.29712800
C	3.63098800	-2.32989700	-2.15943200
H	4.70591000	-2.15890100	-2.28429600
H	3.10715800	-1.67316500	-2.86119200
H	3.41638500	-3.36809700	-2.43599800
C	-2.07685400	0.03514200	2.72894300
H	-2.44701700	-0.82372100	2.16932100
H	-2.92712400	0.69769300	2.91791100
H	-1.70435500	-0.30265500	3.69912900
C	1.66938100	4.02820100	0.31122000
H	1.84113500	3.84655200	1.37634800
H	0.76968600	4.64333800	0.21116500
H	2.50991000	4.61982500	-0.06667900
H	-2.25472800	-3.57325500	-2.31822800
H	-0.48541700	-3.63038400	-2.17656500
H	-1.32311500	-2.11503900	-2.57099100
H	0.00003000	-0.00157500	-4.32092500

12

Benzene

C	-1.16481500	0.76562000	0.00000100
C	-1.24543600	-0.62598300	-0.00000600
C	-0.08065000	-1.39154700	0.00000900
C	1.16484300	-0.76557800	-0.00000100
C	1.24545800	0.62593800	-0.00000600
C	0.08060000	1.39155000	0.00000400
H	-2.07258400	1.36221800	-0.00000200
H	-2.21603900	-1.11373900	-0.00000700
H	-0.14341100	-2.47600000	0.00000000
H	2.07253600	-1.36229200	0.00000200
H	2.21600100	1.11381500	-0.00000400
H	0.14349700	2.47599400	0.00001400

94

LMgPh

Mg	-0.01022300	1.74091600	0.00121400
N	1.11344900	0.03614400	-0.13598800
N	-1.11266400	0.02213100	0.13545100
N	0.01363600	-2.09743600	-0.00206200
C	0.00499900	-0.72130600	-0.00089700
C	-2.63211500	-0.60730900	-1.68088500
C	2.39252200	-0.34559700	0.30875800
C	-2.38745900	-0.37476000	-0.30827300
C	3.44454400	-0.39001500	-0.62747500
C	-1.52581300	-0.38927600	-2.69705900
H	-0.58395700	-0.72819500	-2.25482600
C	-0.02455600	3.82062000	0.00118400
C	-3.43768700	-0.43341500	0.62919300
C	-1.38030200	1.10881400	-3.00078500
H	-2.25673300	1.47519900	-3.54546000
H	-0.48937700	1.30959100	-3.60461200
H	-1.31747100	1.71658100	-2.09052000
C	-3.92360600	-0.95934400	-2.07775900
H	-4.12860400	-1.15215100	-3.12594500
C	-1.00738000	-2.80298600	0.79585900
H	-1.52998400	-1.99738000	1.31041500

C	1.04354400	-2.78866200	-0.80107600
H	1.55599500	-1.97563200	-1.31417100
C	3.16788700	-0.02468100	-2.07206400
H	2.11174700	-0.25204900	-2.26062700
C	2.63833600	-0.57726700	1.68134400
C	-1.71263700	-1.16618800	-3.99976900
H	-1.88847100	-2.23012000	-3.81409900
H	-0.81933100	-1.07218000	-4.62511700
H	-2.55748900	-0.78410800	-4.58246500
C	1.15570900	4.57578400	0.15224700
H	2.11366700	4.06842900	0.27485600
C	-4.71460800	-0.78640700	0.18943700
H	-5.52956400	-0.84898400	0.90415100
C	-4.95749200	-1.06276400	-1.15147300
H	-5.95412600	-1.34467900	-1.47850000
C	4.72508200	-0.72832800	-0.18669100
H	5.54165400	-0.77987100	-0.90044000
C	3.93346000	-0.91444700	2.07925300
H	4.13948400	-1.10644300	3.12738900
C	-1.21565000	4.55852400	-0.15028200
H	-2.16614900	4.03725800	-0.27263700
C	1.52840100	-0.37369000	2.69654900
H	0.59114200	-0.72346800	2.25303700
C	4.96962100	-1.00400400	1.15406500
H	5.96916300	-1.27451800	1.48185200
C	2.10731200	-3.55661900	-0.01333700
H	2.46301600	-2.98724600	0.84530800
H	2.96547400	-3.73660300	-0.66809600
H	1.74544500	-4.52992400	0.32688000
C	-3.16366400	-0.06724100	2.07407600
H	-2.10458700	-0.28198000	2.26091800
C	-0.39495300	-3.68289200	1.88675400
H	0.01272300	-4.61364400	1.48560100
H	-1.17616500	-3.95225500	2.60476800
H	0.39862400	-3.15945800	2.42294100
C	1.15432800	5.97143400	0.15297700
H	2.08690200	6.51862800	0.27225000
C	1.36450200	1.12223700	3.00162500
H	2.23538600	1.49839100	3.54853700
H	0.46998400	1.31183200	3.60375200
H	1.29660200	1.73028800	2.09189800
C	-1.23451200	5.95404900	-0.15174800
H	-2.17492100	6.48760500	-0.27129900
C	0.44238400	-3.67432800	-1.89355000
H	0.04573800	-4.61043600	-1.49384700
H	1.22720500	-3.93326200	-2.61146500
H	-0.35715400	-3.15978600	-2.42950000
C	1.72315000	-1.14949600	3.99875200
H	1.91190800	-2.21107300	3.81232100
H	0.82811100	-1.06677600	4.62321400
H	2.56274600	-0.75784600	4.58269000
C	-2.06152800	-3.58284600	0.00689200
H	-2.42477300	-3.01617700	-0.85038200
H	-2.91707100	-3.77511800	0.66158600
H	-1.68742700	-4.55073100	-0.33553600
C	3.33438300	1.48786800	-2.26939100
H	2.68518300	2.05006600	-1.59080900
H	3.08515400	1.78063800	-3.29522300
H	4.36741400	1.79156500	-2.06797300
C	-3.99397500	-0.86471400	3.07850400
H	-5.05818600	-0.61206800	3.02212800
H	-3.66507700	-0.64643300	4.09960500

H	-3.89575500	-1.94216200	2.91083400
C	4.00920000	-0.81008200	-3.07685600
H	3.67886000	-0.59408700	-4.09797600
H	3.92412800	-1.88895700	-2.91120700
H	5.07013200	-0.54437300	-3.01874200
C	-3.34837500	1.44281100	2.27431100
H	-2.70687400	2.01409600	1.59598900
H	-3.10154300	1.73679100	3.30037700
H	-4.38525800	1.73423100	2.07461400
C	-0.04518000	6.66489800	0.00043300
H	-0.05305600	7.75186600	0.00013800

168

$(LMgH)_2$

Mg	1.32217500	-0.15936800	0.79722000
Mg	-1.34336500	0.12267700	0.80385200
N	-3.03441100	1.01980300	0.07442000
N	3.03858800	-1.02792200	0.08348000
N	2.83606800	1.15589800	0.50504200
N	4.76456300	0.46342900	-0.73520600
N	-2.89619100	-1.15217900	0.55852400
N	-4.82110600	-0.44320700	-0.67780300
C	3.59338000	0.19127800	-0.06176300
C	-3.27435700	2.20566200	-0.64367200
C	-3.63255300	-0.18383500	-0.03169900
C	4.28236300	2.27659600	2.14362000
C	3.29910100	-2.21031000	-0.63452000
C	-3.89188000	3.27135000	0.05824600
C	3.31390200	2.32400000	1.11218500
C	3.94275100	-3.26229900	0.05957600
C	4.77812400	0.93943400	2.66449800
H	4.92985500	0.27481900	1.80961100
C	-2.79463300	2.40450300	-1.95525300
C	-3.37124500	-2.34376800	1.11632100
C	2.71059300	3.55068400	0.75842200
C	-4.39476800	-2.35067500	2.09323000
C	3.70794600	0.29783900	3.55695900
H	3.54756100	0.90215500	4.45604900
H	4.00342600	-0.71061800	3.86443900
H	2.74697400	0.22599600	3.03912100
C	-4.32122200	3.05638100	1.49701300
H	-4.55781500	1.99188700	1.60505900
C	4.69390400	3.47575500	2.72845900
H	5.44595500	3.45836300	3.51071500
C	4.88571500	1.78241200	-1.39073000
H	3.90609900	2.23448300	-1.23544900
C	5.82721100	-0.55763700	-0.75221600
H	5.47023500	-1.31602300	-0.05667400
C	-4.91512000	-1.04032300	2.65519400
H	-5.02201100	-0.33480700	1.82832700
C	-5.81174500	0.63413900	-0.84117100
H	-5.34359800	1.50558700	-0.39244000
C	4.36184600	-3.05956600	1.50287600
H	4.48639600	-1.98214300	1.65267500
C	2.80942900	-2.42006400	-1.94165000
C	-2.98700900	3.65633600	-2.55322000
H	-2.61946100	3.81980100	-3.56273500
C	-2.71863200	-3.54660000	0.75802400
C	6.10970300	1.01124100	3.40992300
H	6.87786000	1.52226500	2.82075500
H	6.46764100	0.00113500	3.63311300

H	6.01287200	1.53955200	4.36445600
C	-1.99613600	1.35252600	-2.70395900
H	-2.02755100	0.42352600	-2.12459400
C	-4.81052200	-3.57746200	2.61398900
H	-5.60380900	-3.60165300	3.35436500
C	-4.06075200	4.49897600	-0.57662700
H	-4.54058700	5.31591500	-0.04648000
C	3.15056900	4.72461900	1.37110600
H	2.70300200	5.67366400	1.09135100
C	4.15196500	4.69618500	2.33509200
H	4.49374300	5.61811400	2.79667200
C	4.12983300	-4.48720100	-0.57771700
H	4.62982500	-5.29442700	-0.05077200
C	-1.52805600	-3.48957300	-0.17733200
H	-1.67295600	-2.60777900	-0.81183500
C	-3.61919400	4.69384500	-1.88330400
H	-3.75768500	5.65514200	-2.36972600
C	-4.21201700	-4.77162700	2.22226100
H	-4.55408900	-5.71476100	2.63864000
C	3.01410600	-3.66878100	-2.53990200
H	2.64094100	-3.83878200	-3.54623400
C	2.02579900	-1.36686200	-2.70375100
H	2.05230000	-0.43478800	-2.12914600
C	3.67553500	-4.69346300	-1.87713100
H	3.82624600	-5.65258900	-2.36419000
C	-3.15600800	-4.74720800	1.31787600
H	-2.66805000	-5.67621000	1.03845000
C	6.05948700	-1.26481700	-2.08800000
H	5.12049800	-1.58505900	-2.53712400
H	6.66013700	-2.16252400	-1.91018200
H	6.60353600	-0.63904900	-2.79903700
C	-3.88286800	-0.44755100	3.62372200
H	-3.75073100	-1.10143400	4.49236100
H	-4.19914800	0.53956000	3.97758500
H	-2.90705300	-0.33776800	3.14163600
C	1.54544100	3.54637300	-0.20994900
H	1.68516500	2.67995100	-0.86669300
C	-6.27975500	-1.14780400	3.33236100
H	-7.02027100	-1.61733700	2.67687800
H	-6.64487700	-0.15098300	3.60049700
H	-6.22976900	-1.73235600	4.25721500
C	5.07314300	1.67331500	-2.90336400
H	6.07688300	1.33615100	-3.17415700
H	4.93289500	2.66360800	-3.34897900
H	4.34307600	0.99359700	-3.34539900
C	-7.10567100	0.41501000	-0.04765700
H	-7.77997500	-0.30498900	-0.51375200
H	-7.63901100	1.36928000	0.01732400
H	-6.89396300	0.07978400	0.96851500
C	-4.95202400	-1.75543000	-1.34411600
H	-4.34343000	-2.44349400	-0.76199300
C	-3.14940400	3.34810400	2.44220900
H	-2.28132100	2.73282100	2.18949300
H	-3.42137900	3.14068200	3.48280100
H	-2.84639700	4.39805700	2.36801700
C	-2.55002100	1.06540500	-4.10270500
H	-3.60331800	0.77923200	-4.07534700
H	-1.99130800	0.24759600	-4.57024000
H	-2.45686300	1.93969400	-4.75544400
C	-6.10666900	1.01276500	-2.28864800
H	-5.18722900	1.27406700	-2.81432600
H	-6.75876800	1.89154200	-2.30209300

H	-6.61812600	0.21433100	-2.83320400
C	0.55965000	-1.79456800	-2.84100300
H	0.48574400	-2.76463400	-3.34383900
H	-0.00676100	-1.06702300	-3.42997900
H	0.07964200	-1.87007300	-1.86480900
C	-5.56179800	3.85362100	1.89608400
H	-5.36596000	4.93085800	1.92802500
H	-5.89338800	3.55678500	2.89633700
H	-6.38766500	3.68236300	1.19828000
C	-0.52870800	1.78747100	-2.80554600
H	-0.44638200	2.76019500	-3.30201300
H	0.05459500	1.06372800	-3.38233000
H	-0.07300800	1.86133600	-1.81784100
C	-0.24170700	-3.27138100	0.63439900
H	-0.33749600	-2.40290400	1.29903900
H	0.62733200	-3.13464700	-0.01977500
H	-0.04280900	-4.13472200	1.27834300
C	7.15387300	-0.05014600	-0.18426700
H	7.68584800	0.59032600	-0.89168400
H	7.79866500	-0.90977300	0.02464300
H	7.00971600	0.50356200	0.74480000
C	2.61727500	-1.08986900	-4.09017300
H	3.68564200	-0.86663700	-4.04083100
H	2.10996100	-0.23907900	-4.55748600
H	2.49159900	-1.95066000	-4.75551500
C	5.91719700	2.74401800	-0.79429400
H	5.89559000	2.73495300	0.29492800
H	5.67286300	3.76054400	-1.11777300
H	6.93226700	2.52657200	-1.13543700
C	3.23247600	-3.50516600	2.43928500
H	2.30830800	-2.96269100	2.21941800
H	3.49373900	-3.32244500	3.48742300
H	3.02784300	-4.57442300	2.31756300
C	-6.35759000	-2.33957500	-1.30867400
H	-7.06966500	-1.78276000	-1.92347800
H	-6.73143900	-2.39055900	-0.28351300
H	-6.31597000	-3.35993000	-1.70129500
C	1.44264400	4.79391100	-1.08423800
H	1.20971500	5.68800600	-0.49587000
H	0.63714400	4.67142100	-1.81498600
H	2.37565000	4.97997800	-1.62571500
C	5.68298300	-3.73969800	1.85948300
H	5.99208400	-3.45692900	2.87109100
H	6.47915200	-3.45015000	1.16593000
H	5.60280900	-4.83178700	1.83986300
C	0.23810200	3.32735300	0.56755700
H	0.30106600	2.42615900	1.19096100
H	-0.62268900	3.24077100	-0.10471300
H	0.05104900	4.16506500	1.24771300
C	-1.37996000	-4.70811100	-1.08580200
H	-1.14981900	-5.61637600	-0.51841700
H	-0.55601900	-4.55099100	-1.78857500
H	-2.29408200	-4.89041300	-1.65946100
C	-4.35710400	-1.74773500	-2.75272200
H	-4.32963000	-2.77018000	-3.14463300
H	-3.33124100	-1.37188100	-2.72777300
H	-4.93264300	-1.13546400	-3.45049400
H	-0.01721200	-0.05260500	-0.44740400
H	-0.00652600	0.01187500	2.13218300

156
13

Mg	2.26936500	0.25210900	0.24061400
N	3.82356300	-0.98434000	0.71983900
N	3.53990100	1.79590700	-0.18586900
C	3.43947100	-2.31939000	1.03151600
C	2.90494900	2.86022900	-0.87851200
C	5.10675800	-0.62855700	0.75311000
C	5.57145400	0.66932600	0.47011100
H	6.64091900	0.81263300	0.57194600
C	4.85686900	1.78610800	-0.00522700
C	3.09170000	-3.20242800	-0.00966100
C	3.03459700	2.94547700	-2.28264600
C	3.29540700	-2.70758000	2.38212100
C	6.14359000	-1.66936600	1.10219400
H	6.00937800	-2.02634200	2.12824700
H	7.15518500	-1.27454400	0.99901800
H	6.03304300	-2.54392200	0.45361300
C	3.50322200	-1.69765000	3.49527800
H	4.16792900	-0.91428500	3.11891100
C	2.03274900	3.72231500	-0.17870700
C	2.87225000	-4.00645800	2.66438200
H	2.76191600	-4.31959700	3.69816600
C	3.79218100	1.87752500	-3.05268800
H	4.53472000	1.43199000	-2.38538200
C	2.35617500	3.96274700	-2.95563200
H	2.45019200	4.04596700	-4.03416100
C	5.66562600	3.01213100	-0.36129700
H	5.85683800	3.04674000	-1.43930200
H	6.62937900	3.00589900	0.15140300
H	5.12451600	3.92655600	-0.10619400
C	2.67355200	-4.49574000	0.31863900
H	2.41091600	-5.18581200	-0.47853900
C	2.57867200	-4.90542000	1.64226700
H	2.25616200	-5.91504600	1.88051900
C	3.13749700	-2.77491300	-1.46378000
H	3.46767900	-1.73010200	-1.48801300
C	1.81535400	3.55664100	1.31425900
H	1.84719500	2.47617800	1.52201100
C	1.54829600	4.86237600	-2.26793900
H	1.03193800	5.65253000	-2.80561600
C	1.37840500	4.72825000	-0.89444900
H	0.71418400	5.40787600	-0.37113000
C	4.14862300	-2.28908800	4.74883800
H	3.48596200	-3.00025200	5.25328100
H	4.37570100	-1.49456400	5.46685300
H	5.08089600	-2.81126100	4.51099800
C	2.16319000	-1.02947200	3.82913900
H	1.70544000	-0.58437500	2.93742700
H	2.28936300	-0.23891200	4.57685800
H	1.45306500	-1.76604700	4.22084200
C	0.45011200	4.05250100	1.78951700
H	0.36361200	5.14222700	1.72184400
H	0.29951600	3.78312400	2.83802500
H	-0.37008100	3.61180100	1.21466800
C	2.82243900	0.75661700	-3.44903700
H	2.06147800	1.12998100	-4.14198000
H	2.29161500	0.36068500	-2.57501000
H	3.35123400	-0.07400100	-3.92911400
C	2.95000400	4.19861000	2.11954600
H	3.91582200	3.75528000	1.86461900
H	2.78690500	4.05945300	3.19388100
H	3.00265700	5.27454600	1.91939100
C	4.53839100	2.41318900	-4.27484000

H	5.15355400	1.62301900	-4.71713300
H	5.19398900	3.24856800	-4.00896900
H	3.85173500	2.76119900	-5.05369900
C	1.74584500	-2.84556300	-2.10159900
H	1.77586800	-2.48468100	-3.13417300
H	1.02725100	-2.22925900	-1.55144100
H	1.35199300	-3.86655600	-2.11321400
C	4.16003000	-3.58872700	-2.26294200
H	3.89006600	-4.65001500	-2.28830300
H	5.15977400	-3.50869300	-1.82465100
H	4.21097400	-3.23247500	-3.29747700
Mg	-0.44350300	0.13149700	0.06161400
N	-2.03150100	-0.84568700	-0.85248500
N	-2.30668400	0.61303500	0.81587000
N	-4.26165800	-0.50234400	-0.01671800
C	-2.90788700	-0.25379600	-0.02410400
C	-2.81958100	1.83945800	1.25891600
C	-2.82902200	2.09660100	2.64591000
C	-5.00828600	-0.25075000	1.22920900
H	-4.22515900	-0.04193000	1.95771400
C	-2.08813900	-2.13761100	-1.39478600
C	-3.17933200	2.85419600	0.33988700
C	-4.88902600	-1.00371700	-1.25169900
H	-4.09691100	-0.91970600	-1.99509300
C	-2.37386700	1.01605300	3.60550900
H	-2.48269000	0.06137800	3.07784000
C	-2.04432500	-3.28063400	-0.56507900
C	-2.03714400	-3.13838300	0.94607200
H	-2.53945800	-2.20030800	1.19952900
C	-2.02456900	-2.26752100	-2.79896800
C	-3.06588200	2.60920100	-1.15409500
H	-3.27609800	1.55320600	-1.34251000
C	-3.56341000	4.10078200	0.83722600
H	-3.84294500	4.88883800	0.14466800
C	-0.59893100	-3.05293200	1.47061000
H	-0.05397500	-3.98055500	1.27530700
H	-0.58147900	-2.86437000	2.54930100
H	-0.02297900	-2.25427800	0.98571100
C	-2.02610700	-1.01825500	-3.65833300
H	-2.54909500	-0.24207300	-3.08689900
C	-3.21358800	3.35922800	3.09891800
H	-3.22271500	3.56508100	4.16547000
C	-6.03150600	-0.11193600	-1.73728400
H	-6.93374000	-0.23302600	-1.13234800
H	-6.28790300	-0.39300500	-2.76378700
H	-5.74596900	0.94227100	-1.73071900
C	-3.58501100	4.35782500	2.20539000
H	-3.88488700	5.33557800	2.57139000
C	-5.93776600	0.96643500	1.21667700
H	-5.46388500	1.82529500	0.74122100
H	-6.16381900	1.24744400	2.25012300
H	-6.88661400	0.75876200	0.71628900
C	-2.79763000	-4.25514600	1.66397700
H	-2.28592200	-5.21921700	1.57521500
H	-3.80915700	-4.37535000	1.26331700
H	-2.87672700	-4.02962200	2.73255800
C	-3.20692200	0.95062300	4.88547700
H	-3.06911200	1.83968200	5.51032600
H	-2.91205400	0.08416700	5.48683200
H	-4.27472800	0.86325400	4.66013800
C	-1.93934800	-4.53726500	-1.16498500
H	-1.89817900	-5.42431900	-0.53988800

C	-1.92200800	-3.54230100	-3.35616900
H	-1.87320700	-3.65347500	-4.43535200
C	-1.63603900	2.88571100	-1.63339100
H	-1.39130000	3.94739600	-1.53541200
H	-1.50291300	2.59581800	-2.68065100
H	-0.88228500	2.34868100	-1.04393000
C	-4.06581800	3.41107200	-1.98762700
H	-5.08951400	3.27786300	-1.62355700
H	-4.02885800	3.08646800	-3.03256500
H	-3.84238600	4.48316300	-1.97482500
C	-5.74781300	-1.48967000	1.73356500
H	-6.64205300	-1.70506000	1.14299200
H	-6.07408200	-1.31186400	2.76338000
H	-5.10256100	-2.37092100	1.72492200
C	-0.88186300	1.17529900	3.91963800
H	-0.28549600	1.16837900	3.00088000
H	-0.52601000	0.36173300	4.56096500
H	-0.69373500	2.12422000	4.43442400
C	-1.87916800	-4.67452400	-2.54833500
H	-1.79777600	-5.66135300	-2.99517400
C	-5.31985500	-2.47272700	-1.23339100
H	-4.55433800	-3.10555600	-0.78464900
H	-5.46030100	-2.81106800	-2.26486200
H	-6.26412800	-2.62271400	-0.70494200
C	-2.75235700	-1.19506300	-4.99110800
H	-2.83273000	-0.23350500	-5.50879800
H	-3.76265000	-1.59086400	-4.84531800
H	-2.21833200	-1.87786300	-5.66083500
C	-0.59684300	-0.50794200	-3.86865100
H	-0.10046400	-0.32868700	-2.91029400
H	-0.59497200	0.43208800	-4.43181800
H	-0.00008400	-1.24086100	-4.42212000

166

TS2

Mg	-1.42535200	0.65583400	-1.00425000
N	-3.06742800	1.32407600	0.07384700
C	-3.69625500	0.14299000	-0.09086500
N	-4.93689800	-0.16205300	0.42573200
C	-3.67321900	2.57027100	0.29574800
C	-4.50093500	3.15936100	-0.68526500
C	-5.01053700	4.43785800	-0.45003300
H	-5.65393600	4.90325100	-1.19029100
C	-4.70920100	5.12741600	0.72076300
H	-5.11996000	6.11881700	0.88907500
C	-3.87374300	4.54725500	1.67012800
H	-3.63578700	5.09389400	2.57772400
C	-3.34104000	3.27261900	1.47313400
C	-4.76430000	2.42751600	-1.98813600
H	-4.80455300	1.35638700	-1.76943800
C	-3.60092400	2.65331200	-2.96210900
H	-3.54397600	3.70630700	-3.25779500
H	-3.71146000	2.04267000	-3.86443700
H	-2.63601800	2.40460400	-2.50114500
C	-6.09115400	2.79748200	-2.65011800
H	-6.92842600	2.69317600	-1.95278400
H	-6.27850400	2.14453300	-3.50859500
H	-6.08994600	3.82779600	-3.02141700
C	-2.39022000	2.63613900	2.46792100
H	-2.50976300	1.55033600	2.36968500
C	-2.67513600	3.01358800	3.92060900
H	-2.46475200	4.07023800	4.11842700

H	-2.04127800	2.42733500	4.59384000
H	-3.72070300	2.82429900	4.18432700
C	-0.93806500	2.95310200	2.09062700
H	-0.71427200	2.62622700	1.06927400
H	-0.23518500	2.45085000	2.76390400
H	-0.75298100	4.03170900	2.13979100
C	-5.38290900	0.51500200	1.65705400
H	-4.50334100	1.07221400	1.97807500
C	-5.71272400	-0.45852800	2.78974100
H	-6.67184400	-0.95871400	2.63452500
H	-5.78855100	0.10472100	3.72542200
H	-4.93911400	-1.21911600	2.90968300
C	-6.51537000	1.53160700	1.48750500
H	-6.37056900	2.14865300	0.60152600
H	-6.52184300	2.19972800	2.35431500
H	-7.49547800	1.05186700	1.43273000
N	-2.97728600	-0.71090000	-0.84092700
C	-2.87140800	-2.10040400	-0.66976900
C	-2.45054800	-2.66508800	0.55946300
C	-2.19082200	-4.03481600	0.61149300
H	-1.85101800	-4.47496900	1.54268900
C	-2.33943000	-4.84372400	-0.51183600
H	-2.12728400	-5.90728300	-0.44978600
C	-2.76039400	-4.28187200	-1.71171000
H	-2.87462400	-4.91386800	-2.58747000
C	-3.03222500	-2.91624000	-1.80936400
C	-2.22962900	-1.78625200	1.77629600
H	-2.97749900	-0.99031800	1.75051500
C	-0.84848600	-1.12857800	1.74685400
H	-0.06614300	-1.86616600	1.92800800
H	-0.77169500	-0.34254500	2.50308900
H	-0.62593700	-0.67655200	0.77174200
C	-2.40752700	-2.52008300	3.10619800
H	-3.35740700	-3.06297700	3.14671000
H	-2.38696200	-1.80285400	3.93339400
H	-1.59590200	-3.23412500	3.27895500
C	-3.44070400	-2.28615900	-3.12544800
H	-3.95967700	-1.35207000	-2.87974500
C	-4.39334300	-3.15735400	-3.94432800
H	-3.90285200	-4.06387500	-4.31495600
H	-4.75493600	-2.60621200	-4.81886900
H	-5.26017900	-3.46504000	-3.35066800
C	-2.20083000	-1.90125500	-3.94099200
H	-1.55637900	-1.22063700	-3.37619600
H	-2.48356600	-1.41019800	-4.87944900
H	-1.60542200	-2.78898300	-4.18068300
C	-5.74052000	-1.18116900	-0.27541300
H	-5.17208900	-1.37159900	-1.18609300
C	-7.10394300	-0.64884200	-0.71716000
H	-7.80126100	-0.55694500	0.11933400
H	-7.54437200	-1.35170600	-1.43169200
H	-7.01412000	0.32502800	-1.20383500
C	-5.89207400	-2.52690200	0.43952500
H	-4.94823200	-2.85868200	0.87210800
H	-6.19932800	-3.28032500	-0.29257500
H	-6.65445300	-2.49830700	1.22146600
Mg	1.56204600	-0.06724800	-1.00992500
N	3.30006100	1.00493600	-0.53674300
C	3.74094500	-0.05826700	0.18992100
N	5.02243200	-0.22194800	0.66329200
C	4.08978100	1.83103600	-1.35689100
C	4.63915800	1.33151200	-2.55791400

C	5.36886300	2.19354900	-3.37947400
H	5.80197600	1.81809800	-4.30188100
C	5.53842500	3.53148500	-3.03839900
H	6.11374000	4.18933700	-3.68353200
C	4.95150900	4.02926800	-1.87804200
H	5.07026800	5.07932000	-1.62868400
C	4.21871400	3.19902800	-1.02967200
C	4.30686100	-0.08270400	-2.98928400
H	4.18005000	-0.68706400	-2.08683000
C	2.96443000	-0.08073300	-3.73864500
H	3.07203300	0.41383300	-4.70975400
H	2.58799800	-1.09466800	-3.90397400
H	2.18952900	0.50150400	-3.21483400
C	5.38473100	-0.76122500	-3.83091000
H	6.35191700	-0.74500100	-3.32027800
H	5.11643800	-1.80593500	-4.01838300
H	5.50694100	-0.27711800	-4.80544200
C	3.53528800	3.71876200	0.21878400
H	3.51929700	2.89035000	0.93758500
C	4.25185000	4.90096900	0.86821100
H	4.20104200	5.80190700	0.24694600
H	3.78322200	5.14457000	1.82707800
H	5.30811700	4.67844900	1.05170800
C	2.07220500	4.05977800	-0.09170900
H	1.54635500	3.18458900	-0.48640500
H	1.54795300	4.39149400	0.81062400
H	2.01186700	4.85943000	-0.83830900
C	5.90272300	0.94850100	0.80718100
H	5.32736100	1.77332900	0.40095200
C	6.17468900	1.33569500	2.26482800
H	6.87933600	0.67352500	2.76998800
H	6.59856200	2.34520200	2.28552600
H	5.24269800	1.35351700	2.83634700
C	7.17502300	0.88073500	-0.03179900
H	6.92772300	0.70851400	-1.08192000
H	7.69853800	1.83937600	0.03471500
H	7.86564000	0.10306000	0.30490600
N	2.76522500	-0.95934600	0.33116900
C	2.44424200	-2.03626200	1.14343200
C	2.51360600	-2.01306100	2.55503500
C	1.97538100	-3.09208500	3.26119400
H	2.01670500	-3.09295500	4.34574500
C	1.36652200	-4.15613400	2.60220900
H	0.94686500	-4.98009800	3.17254300
C	1.30104700	-4.16536100	1.20973000
H	0.82025500	-4.99505700	0.70368800
C	1.83653600	-3.12123400	0.45844200
C	3.07607900	-0.79432000	3.26033300
H	3.96353100	-0.48000800	2.70529000
C	2.08376500	0.37606600	3.20635300
H	1.18763500	0.14318100	3.79092900
H	2.53417800	1.28533100	3.62074000
H	1.77637100	0.58928200	2.17944200
C	3.51595700	-1.05625200	4.69875800
H	4.19811200	-1.91015000	4.76259800
H	4.03188600	-0.17721000	5.09873200
H	2.66249000	-1.25635400	5.35591500
C	1.78964700	-3.10466600	-1.06144200
H	2.77614700	-2.74181800	-1.38724400
C	1.60187500	-4.50535400	-1.65842000
H	0.58994900	-4.87878600	-1.46840800
H	1.73123600	-4.46163600	-2.74430500

H	2.32366700	-5.22728700	-1.25521100
C	0.70954800	-2.15394700	-1.64571300
H	-0.00810300	-0.56527100	-1.54000700
H	0.72665000	-2.21733300	-2.74264100
H	-0.27388900	-2.51280000	-1.32442500
C	5.49515900	-1.60666400	0.87391000
H	4.61404900	-2.18531100	1.15065800
C	6.03481400	-2.22622000	-0.41671800
H	6.92853200	-1.71340500	-0.77965000
H	6.28940300	-3.27720800	-0.24445100
H	5.27556800	-2.19447900	-1.20106000
C	6.47235300	-1.77660800	2.03192300
H	6.05737200	-1.39818300	2.96816400
H	6.66207300	-2.84609100	2.16163200
H	7.43656900	-1.29415900	1.85391300

84

TS3

Mg	0.03430100	1.13779200	-2.13062300
N	1.11360400	-0.07067900	-0.88534400
N	-1.11668900	0.17679200	-0.78558200
N	0.02873500	-0.46153000	1.22727800
C	-2.40919000	-0.27675600	-0.48821400
C	-2.71528800	-1.65572300	-0.49041700
C	2.35523000	0.40542000	-0.40486600
C	-4.03107700	-2.04266300	-0.22771600
H	-4.28588600	-3.09738900	-0.21645800
C	2.46945000	1.75883800	0.00075900
C	-4.72041600	0.25485900	-0.02032900
H	-5.50339900	0.98516700	0.15900100
C	0.01173600	-0.15072000	-0.11246100
C	-3.41842100	0.68623800	-0.27550800
C	-0.97117600	0.17773600	2.10394800
H	-1.53738600	0.81552900	1.42720800
C	-1.64664100	-2.67162100	-0.85117200
H	-0.71208600	-2.36171800	-0.37460700
C	-3.06005300	2.15641700	-0.36649600
H	-2.02504700	2.25526200	-0.01505400
C	1.26657700	2.69079600	-0.07045700
H	0.40582900	2.14155500	0.33731500
C	3.48688900	-0.42754000	-0.46751200
C	1.10466300	-1.33831800	1.72447500
H	1.61007500	-1.65620700	0.81326400
C	-1.40703600	-2.66237600	-2.36630200
C	3.37504700	-1.82713200	-1.03879200
H	2.30595700	-2.05822600	-1.10511200
C	-5.02678600	-1.10119200	0.01592700
H	-6.04235400	-1.42538400	0.22372700
C	3.72034900	2.21860600	0.41667500
H	3.83107800	3.24632500	0.74588900
C	-0.34285900	1.11229500	3.13980300
H	0.09963400	0.56752100	3.97666700
H	-1.12426600	1.76053200	3.54946100
H	0.42665700	1.74414200	2.69102300
C	-1.94637000	-4.08767100	-0.36331200
H	-2.17006000	-4.10760400	0.70830700
H	-1.08254600	-4.73539800	-0.54244300
H	-2.79723100	-4.52960700	-0.89295600
C	4.71947500	0.07765700	-0.04685900
H	5.59839100	-0.56035300	-0.08079100
C	4.04714000	-2.88896500	-0.16607300

H	3.87441100	-3.88873700	-0.57821700
H	3.65890600	-2.87217300	0.85715700
H	5.13092800	-2.74180500	-0.11057000
C	2.15739200	-0.67252700	2.61446800
H	2.45461500	0.30283800	2.22860600
H	3.05053300	-1.30429900	2.63638100
H	1.81172800	-0.56128000	3.64512800
C	4.83655400	1.38467400	0.40859100
H	5.79888600	1.76337700	0.74108600
C	-3.08270500	2.61450100	-1.83137000
H	-2.48868300	1.95005500	-2.47184100
H	-2.69481600	3.63262000	-1.93918500
H	-4.10402300	2.58911100	-2.22586100
C	-3.93077800	3.06633100	0.49724500
H	-4.96461300	3.10484000	0.13771600
H	-3.54310300	4.08981200	0.47777000
H	-3.94798600	2.72732200	1.53764500
C	0.92808500	3.11124700	-1.53653500
H	1.80668300	3.63408800	-1.93937800
H	0.12267800	3.86245900	-1.49440800
H	0.70106300	2.49109200	-3.00772600
C	0.56949300	-2.61023200	2.38516300
H	0.17828700	-2.42150400	3.38756000
H	1.38960300	-3.32845700	2.48624300
H	-0.21829500	-3.07241300	1.78662500
C	3.92988000	-1.85304200	-2.46817100
H	4.99608000	-1.60079300	-2.47683100
H	3.40973600	-1.12565900	-3.09875900
H	3.81125300	-2.84548000	-2.91717400
C	-1.97822900	-0.76769200	2.75904500
H	-2.35983300	-1.49844700	2.04542600
H	-2.82927400	-0.18111000	3.11872000
H	-1.55505200	-1.29241000	3.61891600
C	1.41239600	3.93009900	0.81862000
H	1.67985300	3.67128800	1.85018900
H	0.46586800	4.47856900	0.83936700
H	2.17377200	4.61372500	0.42873800
H	-2.30856700	-2.98288100	-2.89929000
H	-0.58670600	-3.33560700	-2.63639700
H	-1.14822800	-1.65732800	-2.71095900
H	0.42578100	1.98020000	-3.80104700

82

Int 6

Mg	0.04029400	1.22956200	-2.12789800
N	-1.12567900	0.18445700	-0.83181500
N	1.10431300	-0.06264100	-0.91147400
N	0.01147500	-0.33127300	1.21952400
C	2.35975300	0.39811000	-0.45260400
C	2.52901400	1.76734500	-0.11792800
C	-2.39909800	-0.30118500	-0.51085000
C	3.80087700	2.19362000	0.27044000
H	3.95574900	3.23131500	0.54533600
C	-2.64827400	-1.69242900	-0.45317200
C	4.71301100	-0.00558200	-0.08087800
H	5.56498900	-0.68011800	-0.08270400
C	0.00423700	-0.10316300	-0.14004800
C	3.45921700	-0.48018500	-0.47290100
C	1.06752500	-1.19613300	1.77301400
H	1.56710900	-1.58106500	0.88460100
C	1.36905800	2.75498900	-0.22768300
H	0.50658000	2.28386100	0.27142100

C	3.29122700	-1.90307200	-0.96925800
H	2.21389400	-2.09469900	-1.02564500
C	-1.54261600	-2.67887100	-0.78536900
H	-0.62349500	-2.33268900	-0.30327100
C	-3.44779400	0.62452600	-0.32488800
C	-0.97938400	0.37817400	2.05037300
H	-1.52474800	0.98952300	1.33289100
C	0.98367000	3.04772200	-1.70984700
C	-3.16298100	2.10469100	-0.48084300
H	-2.11445000	2.26070500	-0.19675700
C	4.88366100	1.31730500	0.30476400
H	5.86117600	1.67509300	0.61561100
C	-3.94286800	-2.12309000	-0.15720800
H	-4.15252300	-3.18615800	-0.09940500
C	0.50560200	-2.41310500	2.51136000
H	0.12680400	-2.15518300	3.50325600
H	1.30758900	-3.14537100	2.65064300
H	-0.29853200	-2.88890300	1.94627500
C	1.61324400	4.05740200	0.54101000
H	1.90049600	3.87808900	1.58442100
H	0.69782800	4.65676400	0.53871600
H	2.39541000	4.65923800	0.06640500
C	-4.72750600	0.14879500	-0.03659200
H	-5.53879700	0.85278800	0.12197800
C	-4.02170300	3.00150800	0.40889600
H	-3.68231900	4.03994600	0.33934200
H	-3.96242100	2.69206200	1.45695200
H	-5.07577000	2.98475000	0.11190900
C	-2.01260400	-0.50554400	2.75013900
H	-2.41048500	-1.26355300	2.07486500
H	-2.84910000	0.12033300	3.07640600
H	-1.60419700	-0.99467300	3.63777600
C	-4.97593300	-1.21556800	0.06043700
H	-5.97422000	-1.57354800	0.29489000
C	3.84427800	-2.02804700	-2.39393800
H	3.35228100	-1.31554000	-3.06304800
H	3.68699300	-3.03782200	-2.78945300
H	4.91943300	-1.81802500	-2.41457100
C	3.92079100	-2.94314500	-0.04026200
H	5.00972600	-2.83722300	0.00842800
H	3.70709000	-3.95588700	-0.39814600
H	3.53504000	-2.85571500	0.98028600
C	-1.28236300	-2.68641200	-2.29702400
H	-2.16717700	-3.04092800	-2.83667500
H	-0.43974300	-3.34073500	-2.54424500
H	-1.04518200	-1.67907000	-2.64936600
C	-0.33747800	1.35451600	3.03868400
H	0.08743400	0.84595500	3.90706300
H	-1.10717500	2.04040000	3.40729200
H	0.44891300	1.94485500	2.56291000
C	-3.29325900	2.51054300	-1.95492000
H	-4.33025900	2.40674900	-2.29203300
H	-2.68560400	1.86323400	-2.59955100
H	-2.97814900	3.54725100	-2.11376300
C	2.13541400	-0.50084200	2.62177300
H	2.45505700	0.44156600	2.17675100
H	3.01394500	-1.15032900	2.68419500
H	1.79213500	-0.31773200	3.64294600
C	-1.80147900	-4.09687900	-0.28037600
H	-2.03602700	-4.11033100	0.78893400
H	-0.91465200	-4.71774600	-0.44109700
H	-2.63171500	-4.57334700	-0.81303600

H	1.88285600	3.31182400	-2.27716100
H	0.31248900	3.91556700	-1.73727000

164
12

Mg	-1.22062100	0.38041400	-0.27349800
Mg	1.22061300	-0.38039700	0.27382800
N	-3.19929500	0.82878500	-0.09796800
N	-2.48136000	-1.28409900	-0.23803800
N	3.19925500	-0.82873800	0.09803900
N	2.48132700	1.28414100	0.23822300
N	4.59002600	0.88659700	-0.84995900
N	-4.59005500	-0.88650700	0.85012400
C	3.46295800	0.47005400	-0.17443900
C	-2.02374200	-2.44026700	0.41310800
C	-1.42106800	-2.33626600	1.69417300
C	4.10479800	-3.02128500	-0.32145100
C	4.15475300	-1.80748000	0.39609600
C	-4.15479300	1.80745800	-0.39622200
C	-0.83653500	-3.47524400	2.25062600
H	-0.37834800	-3.41690700	3.23299900
C	-5.07979900	1.63300200	-1.45153600
C	1.41170600	0.99228200	-2.40432500
H	2.39133700	0.53661100	-2.22380600
C	-1.42271300	-4.77775400	0.31147600
H	-1.41326300	-5.72548200	-0.22022100
C	-3.46300600	-0.47001100	0.17455100
C	-2.01584700	-3.66239100	-0.28561900
C	1.42121400	2.33621000	-1.69413200
C	-5.04948600	-2.27042100	0.64303800
H	-4.44489000	-2.61434300	-0.19612900
C	-1.41170200	-0.99241500	2.40451200
H	-2.39138200	-0.53683400	2.22402400
C	3.01397900	-3.20573400	-1.35502400
H	2.82882300	-2.21995500	-1.80154700
C	5.22525000	-0.04724500	-1.79880600
H	4.55111300	-0.90407200	-1.80635700
C	5.07975200	-1.63322200	1.45144700
C	-2.62464200	-3.74340200	-1.67181200
H	-3.19711400	-2.82042400	-1.81507900
C	-5.03081900	0.38066900	-2.30898700
H	-4.87088400	-0.47627100	-1.64842700
C	5.02121100	-4.02759400	-0.01661100
H	5.00072500	-4.95931600	-0.57370600
C	-4.10484600	3.02140100	0.32110100
C	1.27716800	1.10580200	-3.92349700
H	0.28548600	1.47141400	-4.21355500
H	1.40067500	0.11780200	-4.37804600
H	2.02554500	1.77998300	-4.35583300
C	2.02378500	2.44028000	-0.41302900
C	5.04953900	2.27045300	-0.64265400
H	4.44489100	2.61430600	0.19650200
C	-5.22535600	0.04745400	1.79879900
H	-4.55123100	0.90429400	1.80627800
C	-0.35145900	-0.03460400	1.80500500
C	-3.01401400	3.20603400	1.35462700
H	-2.82889800	2.22034200	1.80136400
C	2.01595100	3.66247000	0.28558600
C	-0.83617300	-4.69146900	1.56847500
H	-0.37544700	-5.56612900	2.01831100

C	0.83689800	3.47521300	-2.25076800
H	0.37879300	3.41683300	-3.23317600
C	5.97964700	-2.66632700	1.72189000
H	6.70472600	-2.54778600	2.52055000
C	6.50648500	2.34552400	-0.18473800
H	6.71182000	1.63370400	0.61794700
H	6.70904800	3.35422500	0.18945600
H	7.20483800	2.15718300	-1.00409800
C	0.35136900	0.03463200	-1.80472300
C	2.62455600	3.74350500	1.67186000
H	3.19709700	2.82057800	1.81518300
C	4.79939300	3.24303000	-1.79978000
H	5.55040100	3.14919000	-2.58767700
H	4.85014100	4.26743300	-1.41760000
H	3.80900200	3.10515900	-2.23391300
C	-5.97969800	2.66605600	-1.72216100
H	-6.70478300	2.54736600	-2.52079400
C	-6.50647000	-2.34566900	0.18527800
H	-7.20475300	-2.15719200	1.00466500
H	-6.70901400	-3.35446100	-0.18867900
H	-6.71192400	-1.63403400	-0.61754100
C	0.83663900	4.69151100	-1.56875200
H	0.37609300	5.56618900	-2.01873800
C	-1.27717600	-1.10609000	3.92367200
H	-2.02550100	-1.78038800	4.35591500
H	-1.40077600	-0.11815300	4.37833100
H	-0.28546700	-1.47164900	4.21370600
C	6.60470400	-0.57247900	-1.39378500
H	7.39800100	0.15143500	-1.59468200
H	6.82411100	-1.47234900	-1.97682800
H	6.63261800	-0.84986300	-0.34033000
C	5.03076400	-0.38104900	2.30912700
H	4.87072200	0.47600300	1.64873800
C	-5.02126700	4.02764800	0.01608500
H	-5.00078500	4.95947000	0.57301200
C	1.42303700	4.77785100	-0.31169000
H	1.41365100	5.72563700	0.21990200
C	-3.37135200	4.16984600	2.48367900
H	-2.58357900	4.16801100	3.24401200
H	-4.31304100	3.88839700	2.96554000
H	-3.47044600	5.19982600	2.12410000
C	-6.60479800	0.57260100	1.39362300
H	-6.63265800	0.84983900	0.34012900
H	-6.82425700	1.47254900	1.97652700
H	-7.39809300	-0.15130100	1.59457500
C	-5.96506300	3.84991200	-0.98997700
H	-6.67996000	4.63663100	-1.21344500
C	5.96500400	-3.85004700	0.98948600
H	6.67989200	-4.63681500	1.21281200
C	6.31875300	-0.10459700	3.08210800
H	6.49480400	-0.85238700	3.86298200
H	6.25536000	0.87017600	3.57603200
H	7.19217200	-0.09512300	2.42174700
C	-1.53625200	-3.75381200	-2.74992800
H	-0.90974100	-2.86026900	-2.67220600
H	-1.97927500	-3.77127400	-3.75202900
H	-0.89214500	-4.63496700	-2.65060900
C	-3.57497200	-4.93205800	-1.82870900
H	-3.04540000	-5.88809000	-1.75629300
H	-4.06655200	-4.90394300	-2.80695800
H	-4.35113700	-4.92518500	-1.05678500
C	3.83128100	-0.44524300	3.26301000

H	2.90399700	-0.59675800	2.70513400
H	3.73378400	0.48508600	3.83273200
H	3.94538500	-1.27542500	3.96850600
C	-3.83125100	0.44460000	-3.26278000
H	-3.94523500	1.27466100	-3.96843800
H	-3.73378000	-0.48584100	-3.83232400
H	-2.90400500	0.59613400	-2.70484900
C	1.53600000	3.75374400	2.74981500
H	0.89182800	4.63484600	2.65044100
H	1.97886700	3.77119100	3.75198600
H	0.90958900	2.86014500	2.67194300
C	1.71836100	-3.64482800	-0.66172000
H	1.82611000	-4.65124000	-0.24541600
H	0.87002800	-3.65270600	-1.34925800
H	1.45904000	-2.99974000	0.18655900
C	-5.25672000	-0.49109100	3.23013000
H	-6.02039500	-1.26137200	3.36362700
H	-5.50255000	0.33087200	3.91024000
H	-4.29026000	-0.90443000	3.52727900
C	-1.71838600	3.64493200	0.66121000
H	-1.82604900	4.65132200	0.24483300
H	-1.45920000	2.99976300	-0.18704800
H	-0.87000900	3.65278500	1.34869300
C	-4.79915100	-3.24281100	1.80028200
H	-3.80873200	-3.10479200	2.23430500
H	-4.84983900	-4.26727600	1.41826000
H	-5.55009300	-3.14891400	2.58823600
C	5.25653000	0.49145900	-3.23008000
H	4.29008800	0.90494300	-3.52708000
H	5.50219100	-0.33045800	-3.91030700
H	6.02028100	1.26166500	-3.36357500
C	3.57474400	4.93224200	1.82897000
H	3.04508500	5.88823300	1.75663200
H	4.35097500	4.92553300	1.05711100
H	4.06624000	4.90406200	2.80725900
C	-6.31876200	0.10413700	-3.08201600
H	-7.19223300	0.09482100	-2.42171900
H	-6.25537300	-0.87073000	-3.57575500
H	-6.49471800	0.85178900	-3.86304100
C	3.37138500	-4.16928600	-2.48427900
H	3.47046900	-5.19934800	-2.12493100
H	4.31309700	-3.88771900	-2.96602400
H	2.58365200	-4.16728200	-3.24465300
H	0.58571800	-0.23236400	2.36588700
H	-0.58581400	0.23255600	-2.36553000
H	-0.57405500	0.99367500	2.13862300
H	0.57378500	-0.99367700	-2.13836600

5. References

[1] S. P. Green, C. Jones, A. Stasch, *Science* **2007**, *318*, 1754.

[2] J. Hicks, M. Juckel, A. Paparo, D. Dange, C. Jones, *Organometallics* **2018**, *37*, 4810.

[3] S. P. Green , C. Jones , P. C. Junk , K.-A. Lippert, A. Stasch, *Chem. Commun.* **2006**, 3978.

[4] G. J. P. Perry, J. M. Quibell, A. Panigrahi, I. Larrosa, *J. Am. Chem. Soc.* **2017**, *139*, 11527.

[5] A. Castelló-Micó, S. A. Herbert, T. León, T. Bein, P. Knochel, *Angew. Chem. Int. Ed.* **2016**, *55*, 401.

[6] O. B. Bondarenko, G. L. Karetnikov, A. I. Komarov, A. I. Pavlov, S. N. Nikolaeva, *J. Org. Chem.* **2021**, *86*, 322.

[7] M. Bergström, G. Suresh, V. R. Naidu, C. R. Unelius, *Eur. J. Org. Chem.* **2017**, *2017*, 3234.

[8] P. Klein, V. D. Lechner, T. Schimmel, L. Hintermann, *Chem. Eur. J.* **2020**, *26*, 176.

[9] G. M. Sheldrick, *SHELX-16*, University of Göttingen, **2016**.

[10] S. S. A. Stoll, *J. Magn. Reson.* **2006**, *178*, 42.

[11] Gaussian 16, Revision A.03, M. J. Frisch, G. W. Trucks, H. B. Schlegel, G. E. Scuseria, M. A. Robb, J. R. Cheeseman, G. Scalmani, V. Barone, B. Mennucci, G. A. Petersson, H. Nakatsuji, M. Caricato, X. Li, H. P. Hratchian, A. F. Izmaylov, J. Bloino, G. Zheng, J. L. Sonnenberg, M. Hada, M. Ehara, K. Toyota, R. Fukuda, J. Hasegawa, M. Ishida, T. Nakajima, Y. Honda, O. Kitao, H. Nakai, T. Vreven, J. A. Montgomery, Jr., J. E. Peralta, F. Ogliaro, M. Bearpark, J. J. Heyd, E. Brothers, K. N. Kudin, V. N. Staroverov, R. Kobayashi, J. Normand, K. Raghavachari, A. Rendell, J. C. Burant, S. S. Iyengar, J. Tomasi, M. Cossi, N. Rega, J. M. Millam, M. Klene, J. E. Knox, J. B. Cross, V. Bakken, C. Adamo, J. Jaramillo, R. Gomperts, R. E. Stratmann, O. Yazyev, A. J. Austin, R. Cammi, C. Pomelli, J. W. Ochterski, R. L. Martin, K. Morokuma, V. G. Zakrzewski, G. A. Voth, P. Salvador, J. J. Dannenberg, S. Dapprich, A. D. Daniels, Ö. Farkas, J. B. Foresman, J. V. Ortiz, J. Cioslowski, and D. J. Fox, Gaussian, Inc., Wallingford CT, 2016.

[12] A. D. Becke, *J. Chem. Phys.*, 1993, **98**, 5648.

[13] K. Burke, J. P. Perdew, Y. Wang, in *Electronic Density Functional Theory: Recent Progress and New Directions*; J. F. Dobson, G. Vignale, M. P. Das, Eds.; Plenum: New York, 1998.

[14] (a) A. D. McLean, G. S. Chandler, *J. Chem. Phys.* **1980**, *72*, 5639; (b) W. J. Hehre, R. Ditchfield, J. A. Pople, *J. Chem. Phys.* **1972**, *56*, 2257.

[15] (a) C. Gonzalez, H. B. Schlegel, *J. Chem. Phys.***1989**, *90*, 2154; (b) C. Gonzalez, H. B. Schlegel, *J. Phys. Chem*. **1990**, *94*, 5523.

[16] T. Lu, F. Chen, *J. Comput. Chem*. **2012**, *33*, 580.

[17] W. Humphrey, A. Dalke, K. Schulten, *J. Molec. Graph.* **1996**, *14.1*, 33.
